# Longitudinal single-cell multiomic atlas of high-risk neuroblastoma reveals chemotherapy-induced tumor microenvironment rewiring

**DOI:** 10.1038/s41588-025-02158-6

**Published:** 2025-04-14

**Authors:** Wenbao Yu, Rumeysa Biyik-Sit, Yasin Uzun, Chia-Hui Chen, Anusha Thadi, Jonathan H. Sussman, Minxing Pang, Chi-Yun Wu, Liron D. Grossmann, Peng Gao, David W. Wu, Aliza Yousey, Mei Zhang, Christina S. Turn, Zhan Zhang, Shovik Bandyopadhyay, Jeffrey Huang, Tasleema Patel, Changya Chen, Daniel Martinez, Lea F. Surrey, Michael D. Hogarty, Kathrin Bernt, Nancy R. Zhang, John M. Maris, Kai Tan

**Affiliations:** 1 Center for Childhood Cancer Research, Children’s Hospital of Philadelphia, Philadelphia, PA USA; 2 Department of Pediatrics, University of Pennsylvania Perelman School of Medicine, Philadelphia, PA USA; 3 Department of Pediatrics, Pennsylvania State University College of Medicine, Hershey, PA USA; 4 Medical Scientist Training Program, University of Pennsylvania Perelman School of Medicine, Philadelphia, PA USA; 5 Graduate Group in Genomics and Computational Biology, University of Pennsylvania Perelman School of Medicine, Philadelphia, PA USA; 6 Applied Mathematics and Computational Science Graduate Group, University of Pennsylvania, Philadelphia, PA USA; 7 Department of Statistics and Data Science, University of Pennsylvania, Philadelphia, PA USA; 8 Hemato-Oncology Division, Edmond and Lily Safra Children’s Hospital, Sheba Medical Center, Tel HaShomer, Israel; 9 Cancer Research Center, Sheba Medical Center, Tel HaShomer, Israel; 10 Department of Hematology, The First Affiliated Hospital of Xi’an Jiaotong University, Xi’an, China; 11 Genome Institute, The First Affiliated Hospital of Xi’an Jiaotong University, Xi’an, China; 12 Center for Single Cell Biology, Children’s Hospital of Philadelphia, Philadelphia, PA USA; 13 Department of Bioengineering, University of Pennsylvania, Philadelphia, PA USA; 14 Cell and Molecular Biology Graduate Group, University of Pennsylvania Perelman School of Medicine, Philadelphia, PA USA; 15 Department of Pathology and Laboratory Medicine, University of Pennsylvania Perelman School of Medicine, Philadelphia, PA USA; 16 Present Address: State Key Laboratory of Experimental Hematology, Institute of Hematology and Blood Diseases Hospital, Chinese Academy of Medical Sciences and Peking Union Medical College, Tianjin, China

**Keywords:** Cancer microenvironment, Computational biology and bioinformatics, Epigenomics, Transcriptomics, CNS cancer

## Abstract

High-risk neuroblastoma, a leading cause of pediatric cancer mortality, exhibits substantial intratumoral heterogeneity, contributing to therapeutic resistance. To understand tumor microenvironment evolution during therapy, we longitudinally profiled 22 patients with high-risk neuroblastoma before and after induction chemotherapy using single-nucleus RNA and ATAC sequencing and whole-genome sequencing. This revealed profound shifts in tumor and immune cell subpopulations after therapy and identified enhancer-driven transcriptional regulators of neuroblastoma neoplastic states. Poor outcome correlated with proliferative and metabolically active neoplastic states, whereas more differentiated neuronal-like states predicted better prognosis. Proportions of mesenchymal neoplastic cells increased after therapy and a high proportion correlated with a poorer chemotherapy response. Macrophages significantly expanded towards pro-angiogenic, immunosuppressive and metabolic phenotypes. We identified paracrine signaling networks and validated the HB-EGF–ERBB4 axis between macrophage and neoplastic subsets, which promoted tumor growth through the induction of ERK signaling. These findings collectively reveal intrinsic and extrinsic regulators of therapy response in high-risk neuroblastoma.

## Abstract (web summary)

Analysis of pre- and post-chemotherapy samples collected from 22 patients with high-risk neuroblastoma using single-nucleus RNA and ATAC sequencing and spatial omics characterizes therapy-related changes in cell composition and the tumor microenvironment.

## Main

Neuroblastoma is a cancer of the sympathetic nervous system that mainly arises from the adrenal glands and sympathetic ganglia. It is the most common extracranial solid tumor in children and accounts for about one in six pediatric cancer deaths^1,2^. Neuroblastoma is stratified into low-, intermediate- and high-risk categories^3^. Patients in the non-high-risk categories have an excellent prognosis with minimal therapy^4^. Despite substantial advances in the standard of care, the 5-year survival of high-risk neuroblastoma remains <50%^5^. Clinical and genetic features, such as older age and *MYCN* amplification are associated with worse prognosis. The poor prognosis of treatment-refractory and recurrent neuroblastoma stems from acquired treatment resistance in a heterogeneous tumor microenvironment^6^.

Recent advances in single-cell transcriptomic and epigenomic profiling have significantly advanced our understanding of the intratumoral heterogeneity of neuroblastoma^7–14^. Neoplastic neuroblastoma cells transcriptionally resemble normal fetal adrenal neuroblasts and recapitulate their developmental trajectory^7,8^. Additionally, epigenetic profiling has characterized two core neoplastic states—adrenergic-like (ADRN-like) and mesenchymal-like (MES-like)^15–17^—which has subsequently been supported by transcriptomic profiling in both neuroblastoma preclinical models^18–21^ and primary tumors^9,20,22^. Furthermore, single-cell profiling of the neuroblastoma tumor microenvironment has uncovered distinct immune subsets^10–12,23^. However, the interplay between neoplastic and immune subtypes and the role of chemotherapy in rewiring the tumor-immune microenvironment to promote treatment resistance remain unclear due to the lack of study of matched pre- and post-therapy patient samples.

We present an integrated single-cell multimodal analysis of paired newly diagnosed and post-induction chemotherapy high-risk neuroblastoma using single-nucleus RNA sequencing (snRNA-seq), single-nucleus assay for transposase-accessible chromatin with sequencing (snATAC-seq) and whole-genome sequencing (WGS). We identify tumor cell intrinsic and extrinsic therapy-induced shifts in the neuroblastoma microenvironment and a pro-tumorigenic axis between tumor-associated macrophages (TAMs) and neoplastic cells, revealing new therapeutic strategies for high-risk neuroblastoma.

## Results

### Single-cell longitudinal profiling reveals microenvironmental shifts

**Fig. 1:**
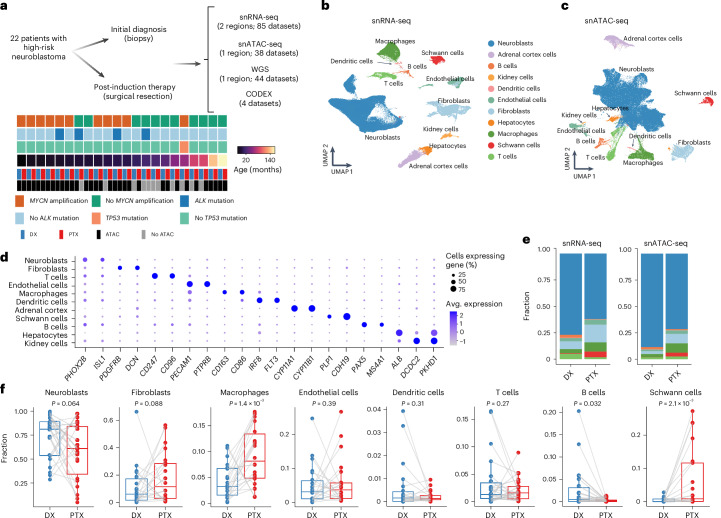
Longitudinal single-cell RNA and ATAC atlas of high-risk neuroblastoma. **a**, Overview of the multiomics studies on patient-matched longitudinal neuroblastoma specimens. **b**,**c**, UMAPs of the snRNA-seq data (**b**; *n* = 372,619 cells) and snATAC-seq data (**c**; *n* = 144,366 cells) annotated by major cell type category. **d**, Dotplot showing the mean expression of marker genes and the percentage of cells expressing them for each annotated cell type. **e**, Stacked barplots of cell type proportions across the snRNA-seq (left) and snATAC-seq (right) datasets. The cell types are colored as in **b**. **f**, Shifts in cell type proportions for each patient between initial diagnosis and post-therapy time points in the snRNA-seq data. Central lines indicate median values, the box edges mark the 25th and 75th percentiles and the whiskers extend 1.5 times the interquartile range. Samples from the same patient between time points are connected by a gray line (*n* = 22 pairs). Statistical significance was assessed using a one-sided Wilcoxon signed-rank test. Avg., average; DX, diagnosis; PTX, post-induction chemotherapy. Source data

**Extended Data Fig. 1:**
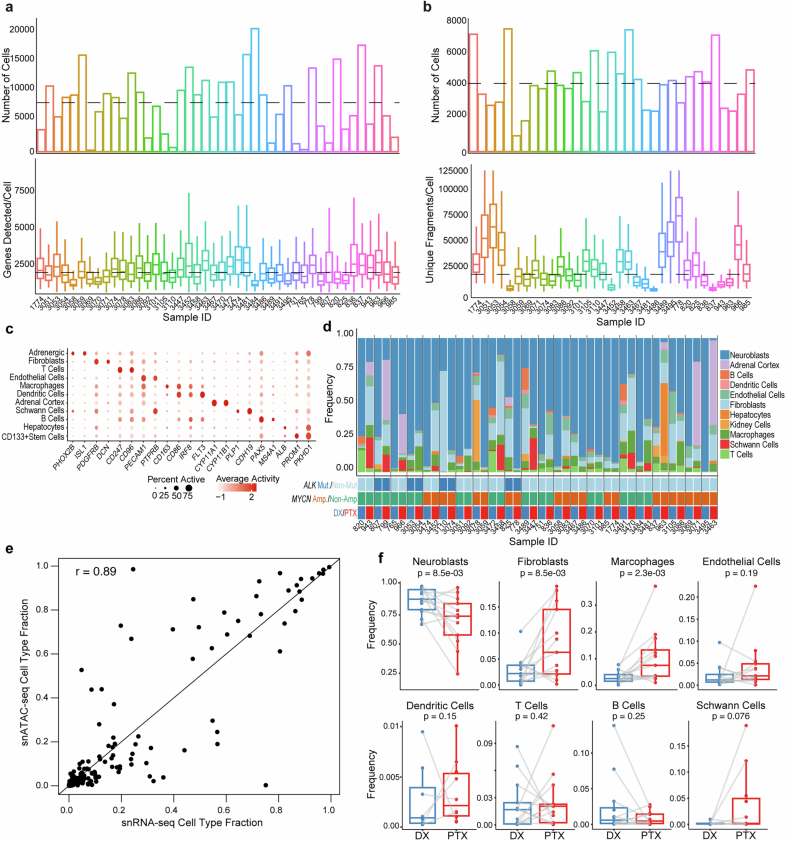
Quality assessment of snRNA-seq and snATAC-seq data. **a)** Bar plots showing the number of cells sequenced (top) and the number of genes detected per cell (bottom) in each snRNA-seq sample after quality control filtering. **b**) Bar plots showing the number of cells sequenced (top) and the number of unique chromatin fragments detected per cell (bottom) in each snATAC-seq sample after quality control filtering. **c**) Dot plot showing the average gene activity and percentage of cells with gene activity for cell type marker genes in the snATAC-seq data. **d**) Cell type composition of each sample based on snRNA-seq data. **e**) Scatter plot of cell type proportions in snRNA-seq and snATAC-seq data. Each dot represents a sample. Pearson correlation coefficient was indicated in the plot. **f**) Shifts in cell type proportion between diagnosis and post-therapy samples in snATAC-seq data. Samples from the same patient between time points were connected by a grey line (n = 22 pairs). Statistical significance was assessed using a one-sided Wilcoxon signed-rank test. Source data

We profiled neuroblastoma samples from 22 patients with high-risk neuroblastoma obtained through an initial diagnostic biopsy followed by a surgical resection after three to four cycles of induction chemotherapy using snRNA-seq (22 pairs), snATAC-seq (13 pairs and seven unpaired) and WGS (22 pairs) (Fig. 1a). Patients were aged 6 months to 13 years at the time of diagnosis, 14 of whom were female and eight of whom were male. *MYCN* amplification and *ALK* and *TP53* mutations were observed at diagnosis in 11, four and one patient(s), respectively (Fig. 1a and Supplementary Tables 1 and 2). Patient response to induction chemotherapy was evaluated by ^123^I-metaiodobenzylguanidine imaging plus anatomic imaging using computed tomography or magnetic resonance imaging, bone marrow aspirate and biopsy^24^ (Supplementary Tables 1 and 2). After quality control, we obtained 372,619 and 144,366 high-quality cells from snRNA-seq and snATAC-seq, respectively (Extended Data Fig. 1a,b).

We identified eight major cell populations, including neuroblasts, fibroblasts, Schwann cells, endothelial cells, macrophages, dendritic cells, T cells and B cells (Fig. 1b–d, Extended Data Fig. 1c and Supplementary Table 3), consistent with the results from recent single-cell studies of patients with neuroblastoma^7,8^. We also detected three tissue-specific cell populations (that is, hepatocytes, adrenal cortex cells and kidney cells) in a few patients (Extended Data Fig. 1d)—probably from adjacent normal tissue—and excluded them from subsequent analysis. The cell type compositions were largely concordant between snRNA-seq and snATAC-seq data (Fig. 1e and Extended Data Fig. 1e). Interestingly, we found several notable changes in the tumor microenvironment due to chemotherapy. The proportion of macrophages was significantly and consistently expanded after therapy in both snRNA-seq and snATAC-seq data. Schwann cells—and fibroblasts to a lesser extent—also expanded after therapy, with large shifts noted in a subset of patients (Fig. 1f and Extended Data Fig. 1f). Taken together, these results demonstrate that neuroblastoma therapy results in large-scale alteration in the composition of the tumor-immune microenvironment.

### Chemotherapy alters neoplastic cell state composition

**Extended Data Fig. 2:**
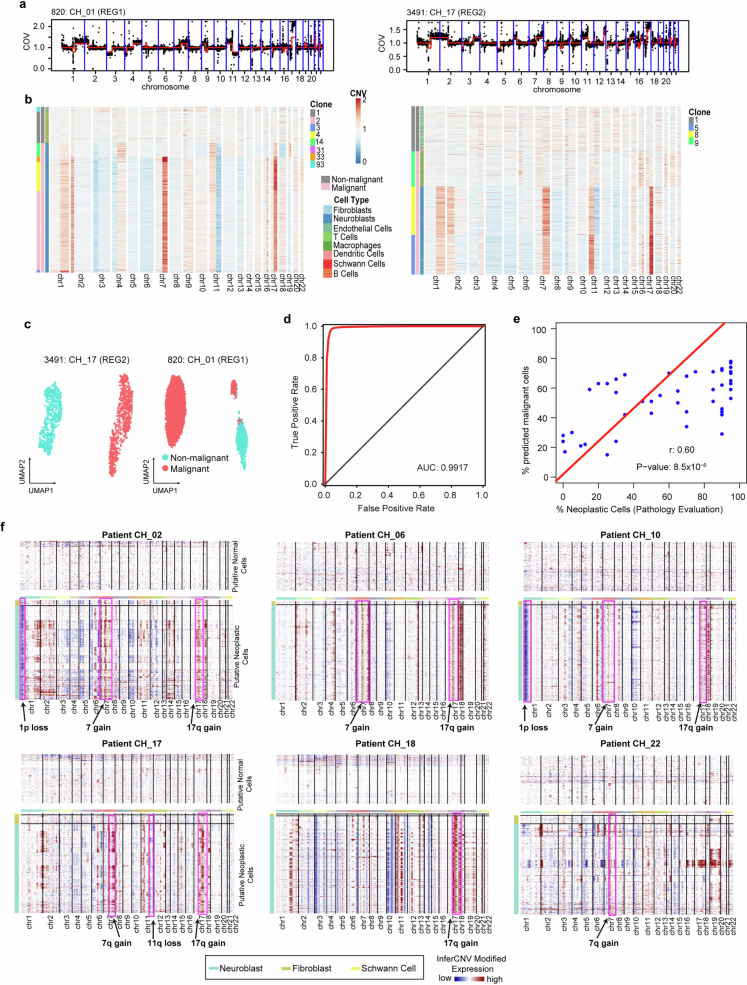
Malignant cell calling by using whole genome sequencing (WGS) and snRNA-seq data. **a)** Genome segmentation for two representative patients using whole genome sequencing (WGS). Black points are equal sized bins. Red line shows the Hidden Markov Model (HMM) copy number states. **b)** Heatmaps of copy number alteration profiles from samples in **(a)** using snRNA-seq data derived from Clonalscope. **c)** UMAP representation of cells from snRNA-seq data from samples in **(a)** colored by predicted malignant or non-malignant prediction. **d)** Receiver Operating Characteristics (ROC) curve for supervised classification by artificial neural networks. Value of the Area Under the Curve (AUC) is shown inside the plot. **e)** Correlation between histologic estimation of neoplastic cell percentage by pathologists (x-axis) and predicted malignant cell proportions (y-axis). Each dot represents an individual sample. Linear regression line is shown in red. Pearson correlation coefficient (r) is shown. Significance was assessed using a two-sided test for association between paired samples. **f)** Heatmaps of inferCNV results of representative patient samples after inference of putative normal cells (top panels) and neoplastic cells (bottom panels). Known recurrent neuroblastoma copy number variations (CNVs) are highlighted with a magenta box. Source data

We sought to dissect the intratumoral heterogeneity by first characterizing neoplastic cell states. We identified neoplastic cells by combining the copy number variation (CNV) profiles derived from WGS data with inferred CNVs from the snRNA-seq data (Extended Data Fig. 2a–e and Supplementary Methods). We restricted our neoplastic cell call to cells derived from the neural crest lineage including neuroblasts, fibroblasts and Schwann cells, as these populations have been suggested to include neoplastic cells^16,21,25^. Of note, the fibroblast population is heterogeneous and may include neural crest-derived endoneurial fibroblasts, which could harbor the same mutations as neoplastic neuroblasts due to their shared precursors during differentiation^26–28^. Overall, we identified 205,253 neoplastic cells and the proportion of these cells was correlated with a pathologist’s manual estimates based on histology (*r* = 0.6; *P* = 8.5 × 10^-6^; Extended Data Fig. 2e). We further validated our neoplastic cell call by confirming the presence of known neuroblastoma CNVs, such as 17q, 7q, 17 and 7 gains and 1p and 11q losses^29^ through analysis with inferCNV^30^ (Extended Data Fig. 2f).

**Fig. 2:**
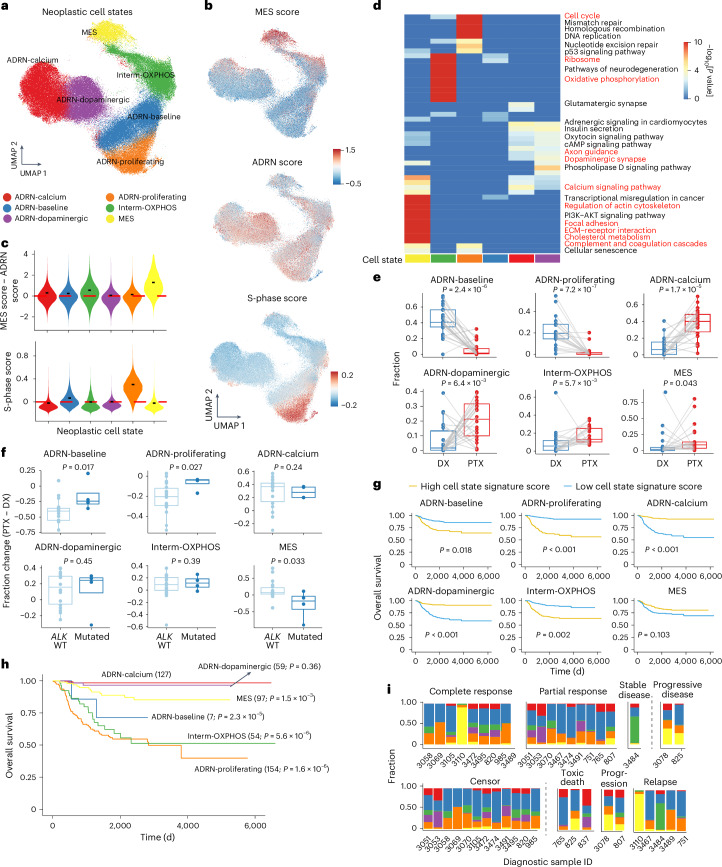
Therapy-induced neoplastic cell state shifts. **a**, UMAP of inferred neoplastic cells from snRNA-seq data after integration and annotation of cell states. **b**, UMAPs showing the MES, ADRN and cell cycle S-phase signature scores of neoplastic cells. **c**, Violin plots of MES − ADRN signature score difference (top) and cell cycle S-phase score (bottom) across different cell states. The short black bars represent the median value in each group and the red dashed lines indicate *y* = 0. **d**, Heatmap of the top 15 enriched Kyoto Encyclopedia of Genes and Genomes pathways for each neoplastic cell state. The enrichment was conducted based on Fisher’s exact test using enrichR without multiple comparison adjustment. We highlighted in red some labels that are closely related to the naming of each cell state. **e**, Shifts in cell state proportion between diagnosis and post-therapy samples. A one-sided Wilcoxon signed-rank test was used to calculate statistical significance. Samples from the same patient between time points are connected by a gray line (*n* = 22 pairs). **f**, Differences in cell state frequencies between paired post-therapy and diagnostic samples stratified by *ALK* mutation status. A one-sided Wilcoxon rank-sum test was used to calculate significance (*n* = 18 *ALK* wild type (WT) and 4 *ALK* mutated). In **e** and **f**, central lines indicate median values, the box edges mark the 25th and 75th percentiles and the whiskers extend 1.5 times the interquartile range. **g**, Kaplan–Meier curves of overall survival based on neoplastic cell state using the Sequencing Quality Control project dataset. Patients were stratified into high and low groups based on the median value of the cell state signature score. *P* values were calculated based on the Cox proportional hazards model and adjusted by age, sex and *MYCN* amplification status. **h**, Kaplan–Meier curves of overall survival, with patients grouped into different neoplastic cell states based on maximum cell state signature scores. The numbers of samples per group are indicated. *P* values were calculated based on the Cox proportional hazards model and adjusted by age, sex and *MYCN* amplification status. The ADRN-calcium state was chosen as the baseline state. **i**, Proportions of neoplastic cell states in the initial diagnostic samples. Patients are grouped according to their responses to induction chemotherapy (top) and clinical events (bottom). A one-sided *t*-test was performed to compare the MES state proportion between two patient groups, as indicated by the vertical dashed line (*P* = 0.02 (top) and 0.05 (bottom)). cAMP, cyclic AMP; ECM, extracellular matrix. Source data

**Extended Data Fig. 3:**
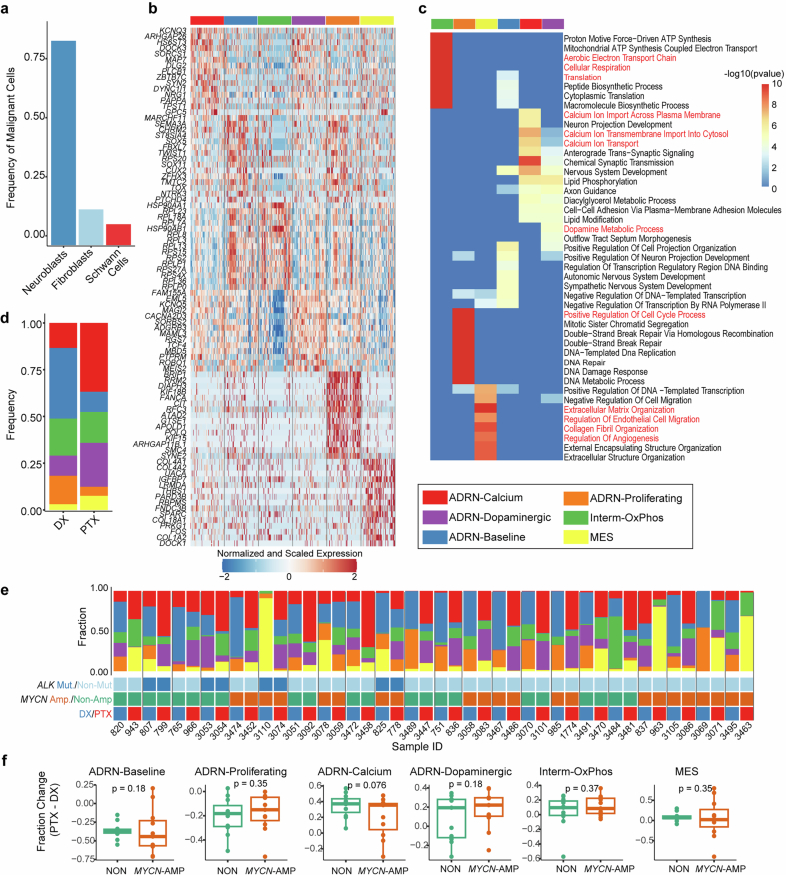
Malignant cell annotation and survival analysis. **a)** Barplot of fraction of malignant cells in each cell type. **b**) Heatmap showing normalized expression of top 15 up-regulated genes in each neoplastic cell state, down sampled to 100 cells per state for illustration purpose. **c**) Heatmap of top 15 enriched Gene Ontology Biological Process terms for each neoplastic cell state. **d**) Stacked barplots of neoplastic cell state proportions between diagnosis and post-therapy timepoints. **e**) Malignant cell state proportions in snRNA-seq data of each patient across timepoints, *ALK* mutation, and *MYCN* amplification status. **f**) The changes in cell state proportions between diagnosis and post-therapy samples stratified by *MYCN* amplification status. (n = 11 *MYCN-*amplified, 11 *MYCN-*non-amplified). The one-sided Wilcoxon rank-sum test was used to calculate significance. Source data

Most putative neoplastic cells had a neuroblastic phenotype (Extended Data Fig. 3a). By reintegrating and clustering the neoplastic cells from all samples, we found six distinct neoplastic cell states (Fig. 2a and Supplementary Table 3). We annotated these populations by their ADRN and MES signature scores, differential gene expression and enriched transcriptional pathways. We identified one MES-high cluster, four ADRN-high clusters and one intermediate cluster with moderate ADRN and MES signatures (Fig. 2b,c). Furthermore, we examined the enrichment of Kyoto Encyclopedia of Genes and Genomes pathways and Gene Ontology biological process terms of the differentially upregulated genes. ADRN-like cells exhibited four states, three of which were enriched in neurodevelopmental pathways (ADRN-calcium (calcium/synaptic signaling), ADRN-dopaminergic (dopamine metabolism) and ADRN-baseline (few differentially expressed genes)) and one of which was enriched for proliferating cells (ADRN-proliferating) (Fig. 2d, Extended Data Fig. 3b and Supplementary Table 4). The intermediate state highly expressed many ribosomal genes and was uniquely enriched in the oxidative phosphorylation pathway (Fig. 2a). It was therefore annotated as Interm-OXPHOS. The MES state differentially expressed extracellular matrix-related pathways (Fig. 2b–d and Extended Data Fig. 3b,c).

Next, we sought to determine how these newly defined populations shifted during therapy and found significant changes (Fig. 2e and Extended Data Fig. 3d). As expected, the abundance of ADRN-baseline and ADRN-proliferating populations decreased after therapy. Conversely, the ADRN-calcium, ADRN-dopaminergic and Interm-OXPHOS populations exhibited significant increases after therapy (Fig. 2e). MES cells made up <10% of neoplastic cells in most samples, but some samples contained a high frequency of MES cells with significant post-therapy changes (Fig. 2e and Extended Data Fig. 3e). Patients with mutated *ALK* demonstrated a significantly smaller decrease in the ADRN-baseline and ADRN-proliferating populations and a notable decrease in the MES state after therapy (Fig. 2f), in contrast with the overall trend (Fig. 2e). *MYCN* amplification did not affect neoplastic state shifts (Extended Data Fig. 3f).

**Extended Data Fig. 4:**
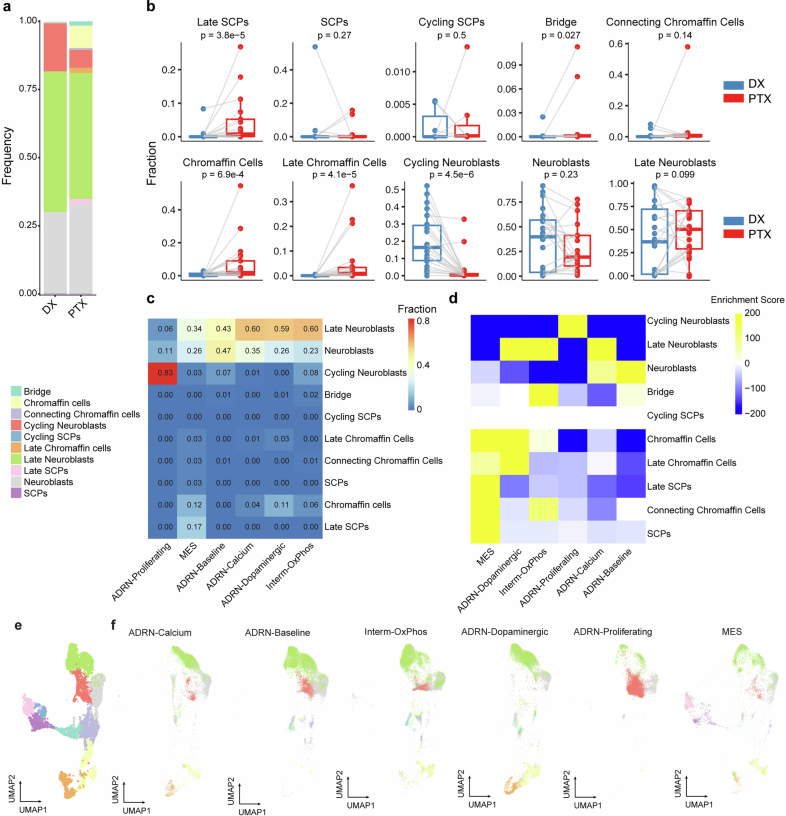
Projection of neoplastic cells onto normal adrenal medullary developmental trajectories. **a**) Stacked barplots of projected cell type proportions across initial diagnosis and post-therapy samples. **b**) Shifts in projected cell type proportions for each patient between initial diagnosis and post-therapy time points. Samples from the same patient at different time points were connected by a grey line (n = 22 pairs). Significance was assessed using a one-sided Wilcoxon signed-rank test. SCPs, Schwann cell precursors. **c**) Heatmap showing projected cell type fractions for each neoplastic cell state. **d)** Heatmap showing the enrichment of projected cell types for neoplastic cell states. Pearson residuals were first calculated based on the contingency table to test the independence of the projected cell type from the neoplastic cell states. The p-value for each cell in the heatmap was calculated based on the assumption that the Pearson residual follows a normal distribution. The enrichment score was defined as the -log10(p-value) multiplied by the sign of the Pearson residual. **e)** The reference UMAP of normal adrenal medullary developmental trajectories. **f**) Visualization of neoplastic cells on the reference UMAP stratified by cell state. Source data

To better understand these cell states, we projected all inferred neoplastic cells onto a single-cell transcriptomic atlas of normal adrenal medullary development^7^. Consistent with previous studies^7,8^, neoplastic cells mostly recapitulated neuroblasts and late neuroblasts, indicating a developmentally arrested state. Interestingly, we found a significant increase in additional developmentally arrested phenotypes in post-therapy samples, including late Schwann cell precursors, a bridge cell population, chromaffin cells, late chromaffin cells and cycling neuroblasts (Extended Data Fig. 4a–c). Moreover, the cell states we identified were associated with different developmental states (Extended Data Fig. 4c–f). The MES state was enriched in non-neuroblastic phenotypes. In contrast, the ADRN-calcium, ADRN-baseline and ADRN-proliferating states were almost entirely enriched in the neuroblast lineage, resembling late neuroblasts, neuroblasts and cycling neuroblasts, respectively. The ADRN-dopaminergic state projected mostly onto neuroblasts and late neuroblasts, but partially resembled determined chromaffin cells, supporting its enrichment of dopaminergic pathways.

**Extended Data Fig. 5:**
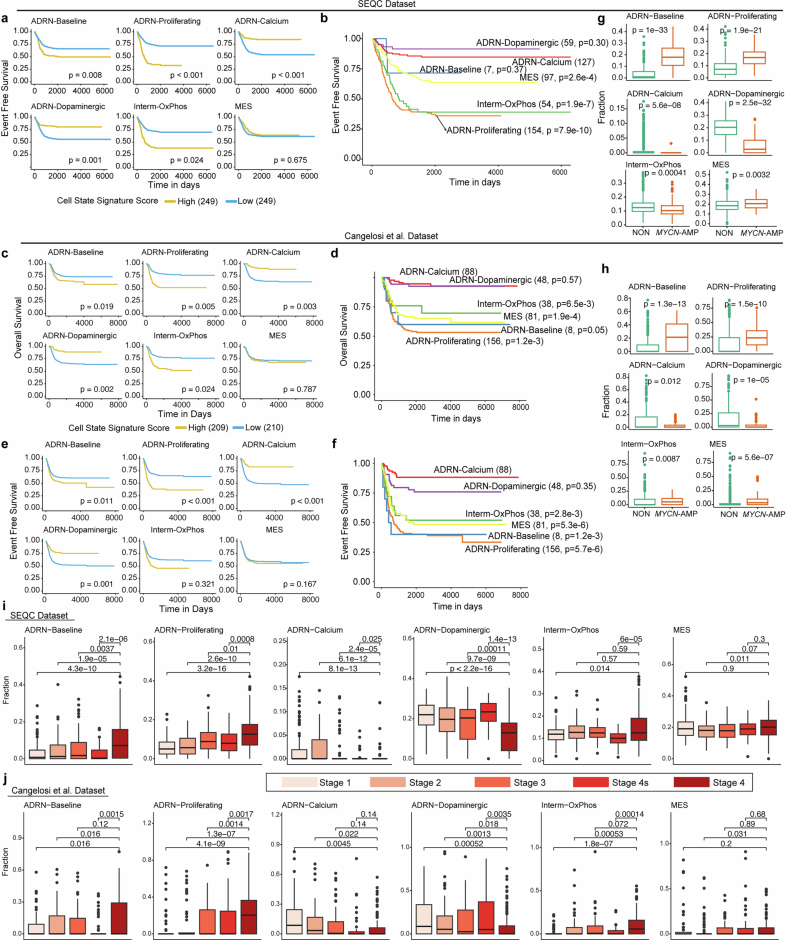
Survival analysis of neoplastic cell state signatures. **a**) Kaplan-Meier curves of event free survival based on neoplastic cell states using the Sequencing Quality Control project (SEQC) neuroblastoma RNA-seq dataset. Patients were stratified into high and low groups based on the median value of the cell state signature score. **b**) Kaplan-Meier curves of event free survival based on the maximum cell state signature scores in the SEQC dataset. Patients were grouped based on the cell state with the maximum signature score. The number of samples and p-value for each group are indicated in the parentheses. The ADRN-Calcium state was chosen as the baseline. **c-f**) Kaplan-Meier curves of overall survival **(c-d)** and event free survival **(e-f)** using the Cangelosi et al. RNA-seq dataset based on patient stratification **(c, e)** and maximum cell state signature score **(d, f)** as in **(a)** and **(b)** respectively. P-values in **a-f** were calculated using the Cox proportional hazards model and adjusted by age, sex and *MYCN* amplification status with no multiple comparison adjustment. **g-h**) Comparison of proportions of neoplastic cell states based on the deconvolution of SEQC (**g**) and Cangelosi et al. (**h**) datasets between *MYCN*-amplified and *MYCN* non-amplified samples. *MYCN*-amplified, non-amplified: n = 96 and 401 (**g**); n = 84 and 333 (**h**). **i-j**) Comparison of proportions of neoplastic cell states based on the deconvolution of SEQC (**i**) and Cangelosi et al. (**j**) datasets between disease stages. Stage1, 2, 3, 4s, 4: n = 120, 78, 62, 52, 181 (**i**); n = 59, 70, 56, 49, 184 (**j**).The one-sided Wilcoxon rank-sum test was used to calculate statistical significance (**g-j**). Source data

Finally, we assessed the clinical implication of these cell states. We examined the gene signature of each state in 498 diagnostic neuroblastoma bulk transcriptomes (the Sequencing Quality Control project cohort)^31^. Interestingly, the samples with a higher gene signature for the ADRN-proliferating, ADRN-baseline or Interm-OXPHOS state showed lower overall survival and event-free survival independent of age, sex and *MYCN* amplification status (Fig. 2g and Extended Data Fig. 5a). In contrast, higher scores of the two states resembling more differentiated stages of the neuroblast and chromaffin lineage (ADRN-calcium and ADRN-dopaminergic) were associated with better prognoses (Fig. 2g and Extended Data Fig. 5b). The MES signature was not significantly associated with prognosis in the diagnostic samples. Orthogonally, we utilized these signatures to stratify patients based on their highest neoplastic cell state signature score. Consistently, patients assigned to the ADRN-proliferating and Interm-OXPHOS groups had the lowest survival, whereas those assigned to the ADRN-calcium and ADRN-dopaminergic groups had the highest survival. Although the MES state was only associated with an intermediate prognosis, a high percentage of the MES state in diagnostic samples correlated with a worse response to chemotherapy and adverse clinical events (toxic death, progression or relapse) (Fig. 2h,i and Extended Data Fig. 5b). We validated these two orthogonal analyses in an additional cohort of 419 bulk transcriptomes^32^, largely replicating these findings (Extended Data Fig. 5c–f). Additionally, deconvolution of the bulk expression data revealed that ADRN-proliferating and ADRN-baseline states were more common in patients with *MYCN* amplification and advanced-stage disease. The MES state is enriched in patients with *MYCN* amplification. Conversely, prognostically favorable states (ADRN-calcium and ADRN-dopaminergic) are less prevalent in patients with *MYCN* amplification and advanced-stage disease (Extended Data Fig. 5g–j). These results suggest that the presence of proliferative, metabolically active and developmentally arrested neoplastic cells at diagnosis portends a less favorable clinical outcome, whereas a more differentiated state with neuronal expression patterns predicts a more favorable outcome. Taken together, neuroblastoma neoplastic cells exhibit multiple distinct transcriptomic states that recapitulate developmental processes and can predict clinical outcomes.

### Cooperative epigenetic regulation governs neoplastic cell states

**Fig. 3:**
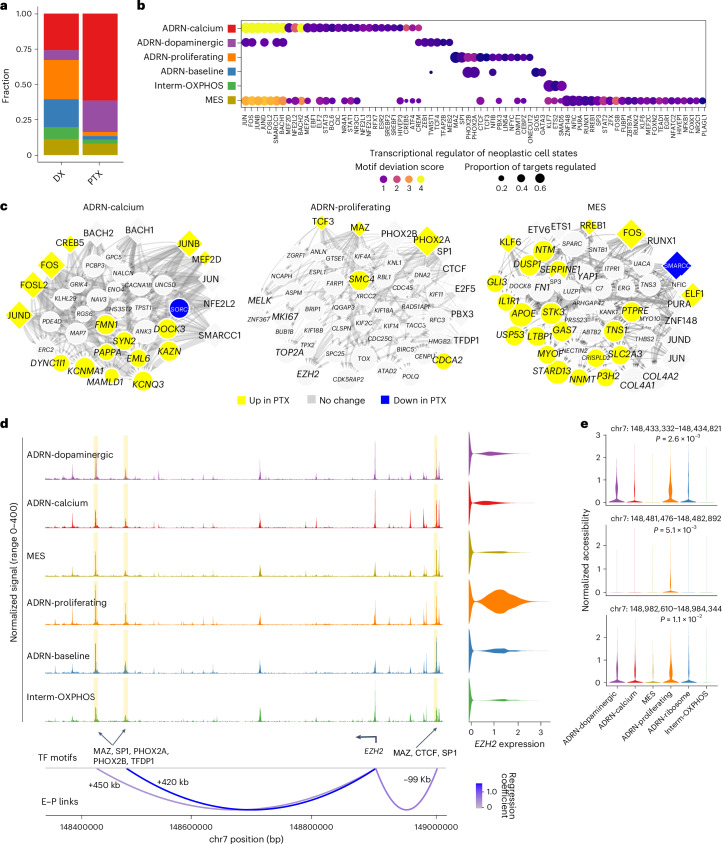
Transcriptional regulation of neoplastic cell states. **a**, Stacked barplots of neoplastic cell state proportions in the snATAC-seq data at the diagnosis and post-therapy time points. The colors are as in **b**. **b**, Dotplot showing the top 15 transcription factors of the transcriptional regulatory network for each neoplastic cell state. The size of each dot represents the fraction of gene targets in the transcriptional regulatory network regulated by each transcription factor. The color represents the chromVAR deviation *z* score. **c**, Transcriptional regulatory networks for the ADRN-calcium, ADRN-proliferating and MES cell states. Diamonds represent transcription factors and circles represent target genes. The size of a transcription factor node is proportional to the average difference in the motif chromatin accessibility *z* score between a given cell state and the rest of the cell states. The size of a target gene node is proportional to the average fold-change of gene expression between a given cell state and the rest of the cell states. The node color indicates the direction of gene expression change between diagnosis and post-therapy samples in each cell state. The edge weight is proportional to the linear regression coefficient for the predicted enhancer–promoter interaction and the fraction of cells that are accessible at the enhancer peak. **d**, Coverage plot showing normalized chromatin accessibility for neoplastic cell states at the *EZH2* locus. The E–P link track represents the predicted enhancer–promoter links colored by the regression coefficient. The transcription factor (TF) motifs present at the enhancer peaks are indicated. Differentially accessible peaks for the ADRN-proliferating state are highlighted. **e**, Normalized chromatin accessibility of putative *EZH2* enhancers across neoplastic cell states. *P* values were calculated using edgeR on pseudo-bulk data without multiple comparison adjustment (Supplementary Methods). kb, kilobases. Source data

**Extended Data Fig. 6:**
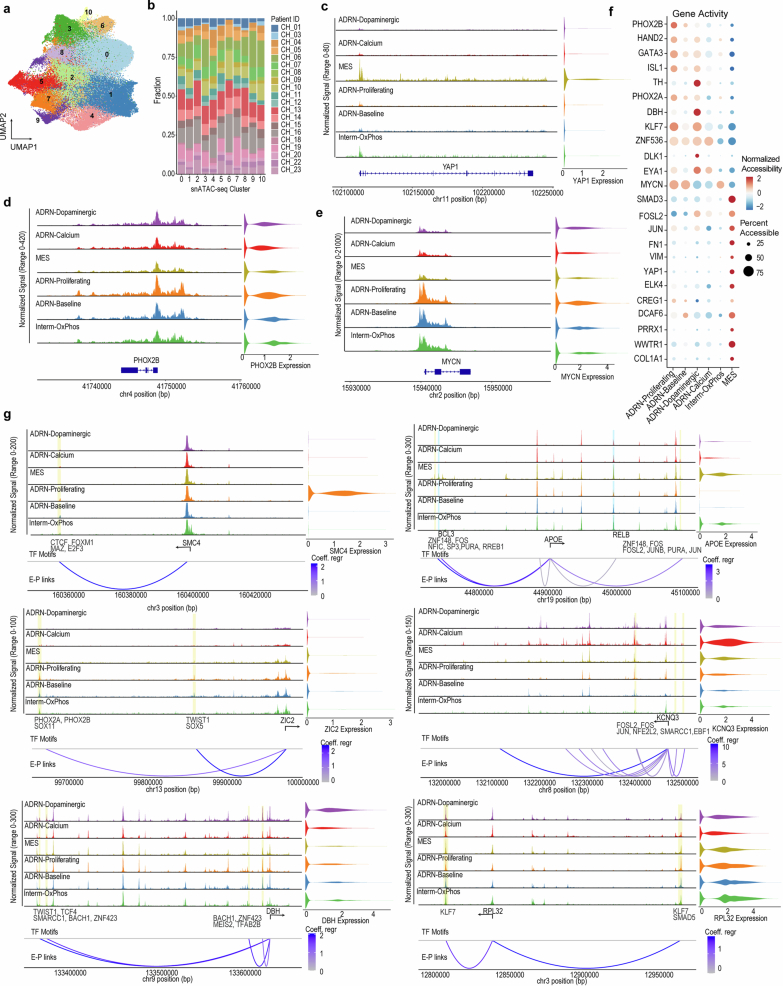
Neoplastic cell states in snATAC-seq data. **a**) UMAP of integrated snATAC-seq data (93,261 cells) annotated by neoplastic cell clusters. **b**) Patient proportions in each of the snATAC-seq clusters. **c-e**) Coverage plots showing normalized chromatic accessibility for each neoplastic cell state at the *YAP1* (**c**), *PHOX2B* (**d**), and *MYCN* (**e**) loci. **f**) Dot plot of the average gene activity and percentage of accessible cells of the ADRN and MES genes for each predicted neoplastic population. **g**) Coverage plots for differentially expressed genes and associated chromatin peaks in each neoplastic cell state: *SMC4* (ADRN-Proliferating)*, APOE* (MES), *ZIC2* (ADRN-Baseline), *KCNQ3* (ADRN-Calcium), *DBH* (ADRN-Dopaminergic), *RPL32* (Interm-OxPhos) loci. The E-P link track represents the predicted enhancer-promoter links colored by the regression coefficient, and the TF motifs present at the enhancer peaks are indicated. The differential accessible peaks (DAPs) for the corresponding cell state are highlighted in blue and yellow for promoter and enhancer peaks, respectively. Source data

**Extended Data Fig. 7:**
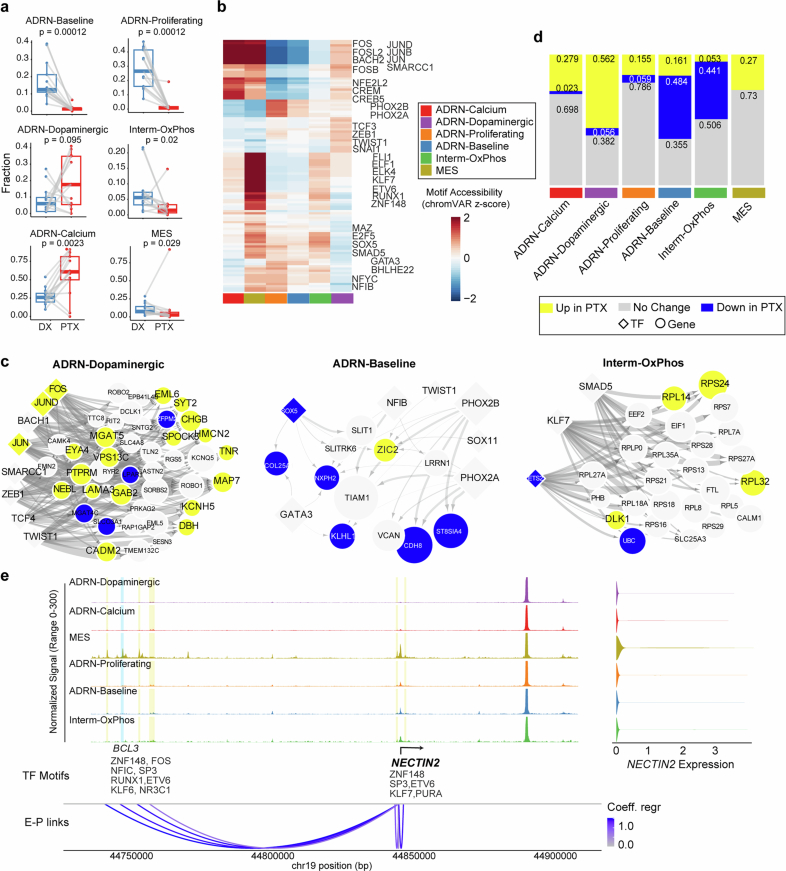
Epigenetic regulation of neoplastic cell states. **a)** Shifts in cell state proportions in snATAC-seq between diagnosis and post-therapy samples. Samples from the same patient at different time points were connected by a grey line (n = 13 pairs). Significance was assessed using a one-sided Wilcoxon signed-rank test. **b)** Heatmap of top 20 transcription factors (TFs) with differential motif chromatin accessibility in each cell state. **c**) Transcriptional regulatory networks (TRNs) for ADRN-Dopaminergic, ADRN-Baseline and Interm-OxPhos cell states. Diamond represents TF and circle represents target gene. The size of a TF node is proportional to the average difference in motif chromatin accessibility z-score between a given cell state and the rest of cell states. The size of a target gene node is proportional to the average fold change of gene expression between a given cell state and the rest of cell states. Node color indicates direction of gene expression change between diagnosis and post-therapy samples in each cell state. The edge weight is proportional to the linear regression coefficient for the predicted enhancer-promoter interaction and the fraction of cells that are accessible at the enhancer peak. **d**) Fractions of state-specific genes in each TRN that were upregulated, downregulated, or non-significantly changed post-therapy. **e**) Coverage plot showing normalized chromatin accessibility and gene expression for the MES state-specific gene *NECTIN2*. The E-P link track represents the predicted enhancer-promoter links colored by the regression coefficient, and the TF motifs present at the enhancer peaks are indicated. The differential accessible peaks (DAPs) for the MES state are highlighted in blue and yellow for promoter and enhancer peaks, respectively. Source data

After defining and characterizing neoplastic cell states, we sought to determine how these cell states are transcriptionally regulated. We identified 11 distinct epigenetic clusters in putative neoplastic cells in the snATAC-seq data (Extended Data Fig. 6a,b). The cells were then computationally mapped onto the transcriptional states to generate high-confidence epigenetic profiles for each neoplastic state. We confirmed that canonical MES and ADRN marker genes, including *YAP1* and *PHOX2B*, were more accessible in their respective states and that the *MYCN* gene was more accessible in the clinically unfavorable states (Extended Data Fig. 6c–g). Consistent with the snRNA-seq data, the ADRN-calcium and ADRN-dopaminergic states expanded after therapy, whereas the ADRN-proliferating and ADRN-baseline states retracted (Fig. 3a and Extended Data Fig. 7a).

We examined the differentially accessible transcription factor motifs for each state using chromVAR^33^ (Extended Data Fig. 7b and Methods). We found that activator protein-1 (AP-1) motifs (for example, FOS, BACH2 and JUN) and CREB motifs (for example, CREM and CREB5) were differentially accessible in the ADRN-calcium, ADRN-dopaminergic and MES states. Canonical adrenergic transcription factor motifs^15,16^ (PHOX2A, PHOX2B and GATA3) were differentially accessible specifically in the ADRN-proliferating and ADRN-baseline states. Likewise, known MES state transcription factor motifs^15,16^ (ETS2, ETV6, ELF1, KLF7 and RUNX1) were most accessible in the MES state and modestly accessible in the Interm-OXPHOS state. Transcription factor motifs associated with epithelial-to-mesenchymal transition^34^ (TWIST1, ZEB1 and SNAI1) were enriched in the ADRN-dopaminergic state.

Next, we constructed a transcriptional regulatory network for each state by integrating snRNA-seq and snATAC-seq data (Supplementary Tables 5–8). We confirmed the significance of AP-1 transcription factors in the MES, ADRN-calcium and ADRN-dopaminergic states (Fig. 3b), as previously reported^15–17^. Interestingly, both AP-1 transcription factor-encoding genes and many of their targets were upregulated after therapy, suggesting a strengthened MES phenotype in response to therapy (Fig. 3c and Extended Data Fig. 7c). Our analysis also nominated new transcription factors involved in regulating each state. For example, ZNF148 and MAZ were predicted regulators of the MES and ADRN-proliferating states, respectively (Fig. 3b,c). Strikingly, despite a significant retraction in the ADRN-proliferating population after therapy, the expression of many state-specific genes (for example, *EZH2*, *TOP2A* and *MKI67*) was unchanged or increased (Fig. 3c, Extended Data Fig. 7d and Supplementary Table 6). These results indicate that this clinically unfavorable population retains its phenotype during therapy, raising the possibility that these proliferating adrenergic cells are persistent and implicated in treatment resistance.

Beyond global transcription factors, we also identified *cis*-regulatory enhancer–promoter interactions associated with key state-specific genes (Fig. 3d,e and Extended Data Figs. 6g and 7e). Focusing on the ADRN-proliferating state, we identified three *EZH2* enhancers correlated with the *EZH2* expression (Fig. 3d). *EZH2*, the catalytic subunit of Polycomb repressive complex 2 (PRC2), is a promising target in high-risk neuroblastoma^19,35,36^. It was highly expressed in this state, with the three enhancer peaks showing elevated accessibility and MAZ, CTCF and PHOX2A motifs, indicating state-specific *EZH2* activation. We also identified ADRN-proliferating-specific *SMC4* enhancers with MAZ and CTCF motifs (Extended Data Fig. 6g). *SMC4* is a core condensin subunit involved in genome organization and tumorigenesis^37,38^. These findings suggest that regulation of chromatin structure may help to maintain the proliferative state. Lastly, we found that *NECTIN2*, recently implicated in T cell dysfunction in high-risk neuroblastoma^11^, was upregulated in the MES state and associated with multiple enhancers (Extended Data Fig. 7e). Overall, this analysis uncovered extensive transcription factor cooperativity driving state-specific gene expression and enhancer-driven mechanisms supporting high-risk neoplastic subsets.

### Pro-tumorigenic macrophages are enriched after therapy

**Fig. 4:**
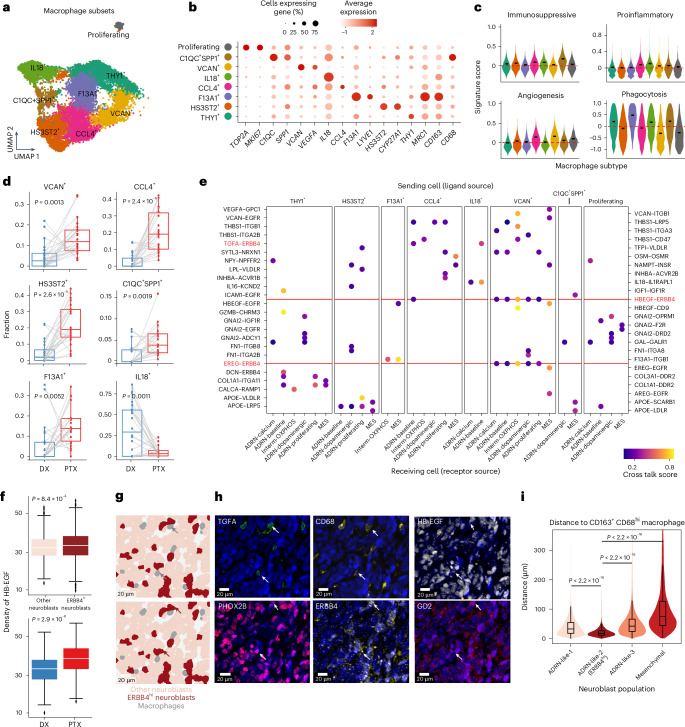
TAMs in the neuroblastoma microenvironment. **a**, UMAP of macrophage subsets (14,866 cells) from snRNA-seq data after integration and annotation. The colors are as in **b**. **b**, Dotplot of the average expression of marker genes and the percentage of cells expressing them for each annotated macrophage subset. **c**, Violin plots of signature scores for immunosuppressive, pro-inflammatory, angiogenesis and phagocytosis macrophages in our macrophage subsets. The short black bars represent the median value in each group and the red dashed lines indicate *y* = 0. **d**, Shifts in macrophage subset proportions between diagnosis and post-therapy samples. A one-sided Wilcoxon signed-rank test was used to calculate significance. Samples from the same patient between time points are connected by a gray line (*n* = 22 pairs). **e**, Dotplot showing predicted ligand–receptor interactions between neoplastic cells and macrophage subsets. The ligands are from macrophage subsets (top labels) and are listed first in each pair. The receptors are from neoplastic populations (bottom labels) and are listed second in each pair. Both *y* axes have been used for labeling due to space constraints. Important interactions involving ERBB4 are highlighted in red; the red dashed line indicates recurrent interactions. **f**, Comparison of the density of HB-EGF protein quantified by CODEX between neighbors of ADRN-like-2 (ERBB4^hi^) neuroblasts and neighbors of other neuroblasts (top), as well as between diagnosis and post-therapy samples (bottom). The density was defined as the mean expression of HB-EGF on cells within a 40-µm window, excluding the marker within the center cell. Significance was assessed using a two-sided Wilcoxon rank-sum test. The numbers of cells are: *n* = 655,573 (other neuroblasts), 17,532 (ERBB4^hi^ neuroblasts), 221,677 (DX) and 451,428 (PTX). **g**,**h**, Representative cell type mask (**g**) and CODEX images (**h**). Arrows indicate macrophages (top) and neuroblasts (bottom). **i**, Distance from each neuroblast cell to the nearest CD163^+^CDCD68^hi^ macrophage across samples, stratified by neuroblast population. Numbers of cells in each group (from left to right): *n* = 396,277, 17,532, 241,995 and 17,301. Significance was assessed using a two-sided Wilcoxon rank-sum test. Outliers were truncated for visualization purposes. In **d**, **f** and **i**, central lines indicate median values, the box edges mark the 25th and 75th percentiles and the whiskers extend 1.5 times the interquartile range. Source data

**Extended Data Fig. 8:**
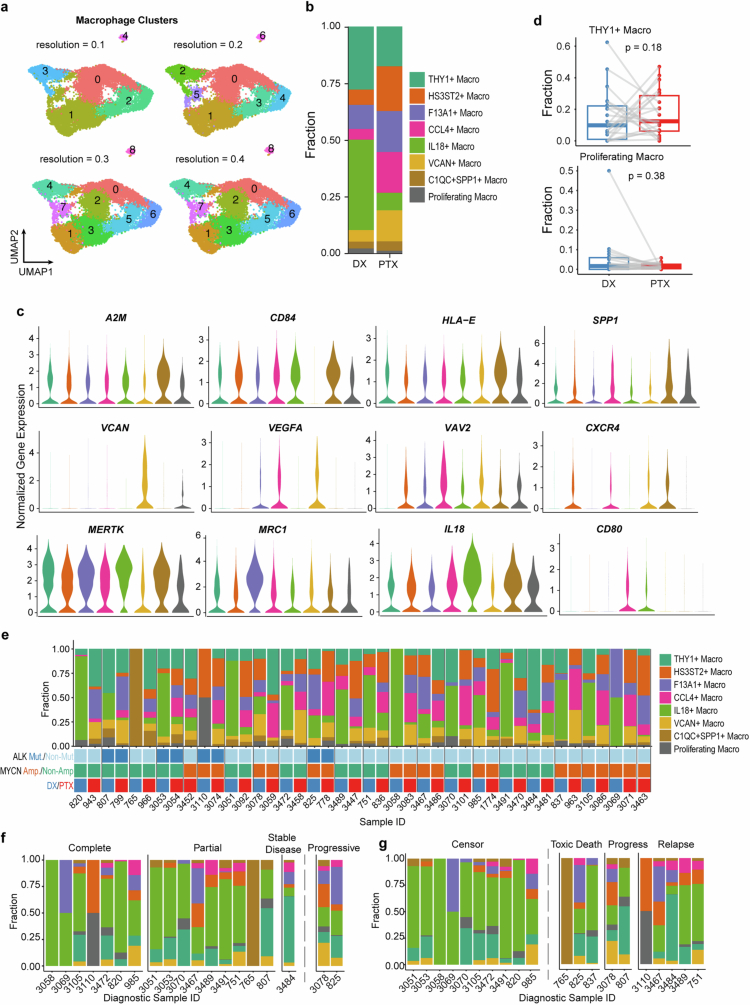
Therapy-induced shifts in macrophage populations. **a**) UMAP plots of macrophages from snRNA-seq data clustered at different resolutions. **b**) Stacked bar plots of macrophage subset proportions in snRNA-seq data at diagnosis and post-therapy timepoints. **c)** Representative signature genes for immunosuppressive (*A2M, CD84, HLA-E, SPP1*), angiogenic (*VCAN, VEGFA, VAV2, CXCR4*), phagocytic (*MERTK, MRC1*) and proinflammatory (*IL18, CD80*) macrophages. **d**) Proportions of *THY1*^+^ and proliferating macrophages in diagnosis and post-therapy samples. Significance was assessed using a one-sided Wilcoxon signed-rank test (n = 22 pairs). **e**) Macrophage subset proportions in snRNA-seq data of each patient across timepoints, *ALK* mutation, and *MYCN* amplification status. **f-g**) Proportions of macrophage subsets in initial diagnostic samples. Patients were grouped according to their responses to induction chemotherapy (**f**) and adverse clinical events (**g**). A one-sided t-test was performed to compare the proportion of *IL18*^*+*^ macrophages between two patient groups, as indicated by the vertical dashed line; p = 0.005 (**f**) and 0.02 (**g**). Source data

Macrophages were the largest immune component in the neuroblastoma microenvironment and expanded significantly after therapy (Fig. 1b,f). TAMs have been shown to contribute to tumor proliferation and therapy resistance in high-risk neuroblastoma^39,40^. Therefore, we sought to delineate the effect of therapy on TAM subtypes. After reintegration and clustering, we identified eight macrophage subsets and annotated them by the top differentially expressed genes (Fig. 4a, Extended Data Fig. 8a,b and Supplementary Table 9). Namely, we identified a proliferating state (*MKI67* and *TOP2A*), a pro-inflammatory state (*IL18*), two pro-angiogenic states (*CCL4* and *VCAN*), an immunosuppressive state (*C1QC* and *SPP1*), a tissue-resident state with the highest expression of a phagocytosis gene (*F13A1*), a lipid-associated state (*HS3ST2*) and an undefined state expressing *THY1* (Fig. 4b,c, Extended Data Fig. 8c and Supplementary Table 10). Notably, although they were previously described as distinct phenotype markers across multiple solid tumors^41^, *C1QC* and *SPP1* had the highest co-expression in one population. In summary, this analysis confirmed that neuroblastoma TAMs can adopt similar phenotypes to those found in other solid tumors.

**Extended Data Fig. 9:**
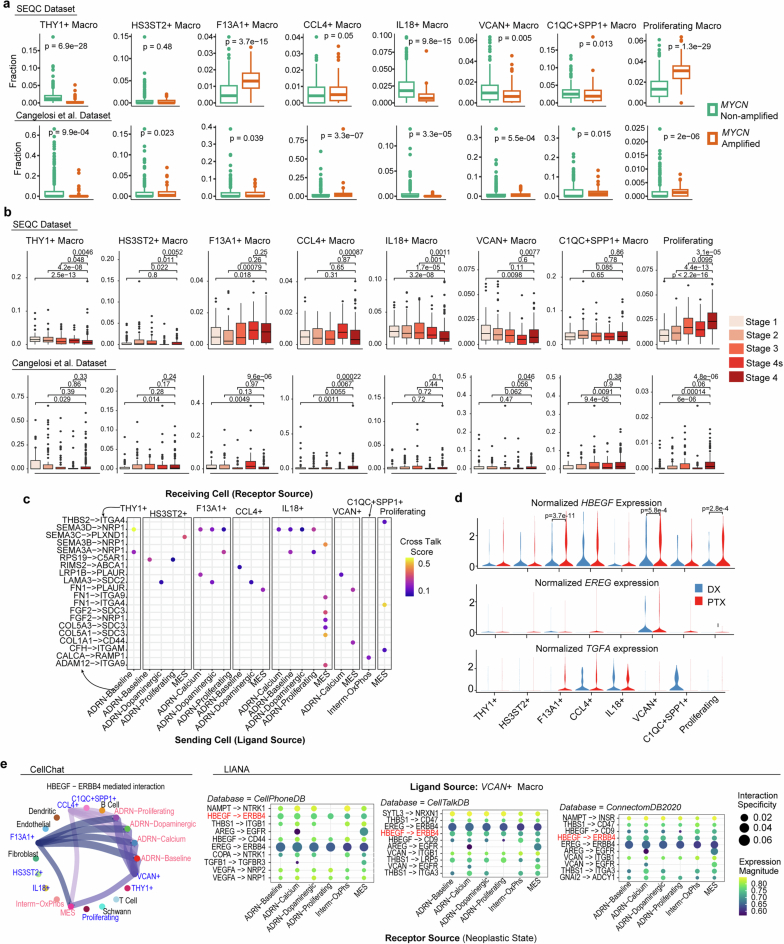
Clinical correlation and transcriptomic validation of macrophage populations. **a**) Comparison of macrophage subset proportions between *MYCN* amplified and non-amplified neuroblastoma patients using CIBERSORTx deconvolution of bulk RNA-seq data (SECQ and Cangelosi et al. datasets). *MYCN*-amplified, non-amplified: n = 96 and 401 (SEQC); n = 84 and 333 (Cangelosi et al.). **b)** Comparison of macrophage subset proportions between disease stages in bulk RNA-seq data as in (**a**). Stage 1, 2, 3, 4s, 4: n = 120, 78, 62, 52, 181 (SEQC); n = 59, 70, 56, 49, 184 (Cangelosi et al.). Significance was assessed in (**a-b**) using a one-sided Wilcoxon signed-rank test. **c**) Dot plot showing predicted ligand-receptor interactions between neoplastic cell states and macrophage subsets. Ligands were from neoplastic cells and receptors were from macrophage subsets. **d**) Normalized gene expression of ERBB4 ligands (*HB-EGF*, *EREG, TGFA*) in different macrophage subsets from DX and PTX samples, upregulated post-therapy using logistic regression with no multiple comparison adjustment (Methods) **e**) Cell-cell interaction analysis using CellChat and LIANA. Left, HB-EGF-ERBB4-mediated interactions among neoplastic cell states and non-neoplastic cells by CellChat. Macrophage subsets are colored in blue and neoplastic populations are colored in red. Right, Top 10 ligand-receptor pairs identified between *VCAN*+ macrophages (ligand source) and neoplastic cell states (receptor source) by LIANA using three representative signaling pathway databases. HB-EGF-ERBB4 interaction is highlighted in red. Source data

Importantly, we observed significant shifts in macrophage states between the paired diagnostic and post-therapy samples. The *IL18*^+^ population was the only state that was reduced after therapy, whereas all other states except the proliferating and *THY1*^+^ state were expanded (Fig. 4d and Extended Data Fig. 8d,e). A predominance of *IL18*^+^ macrophages was linked to better treatment responses and fewer adverse clinical events (Extended Data Fig. 8f,g). Deconvolution of bulk gene expression data showed that *F13A1*^+^, *CCL4*^+^ and proliferating macrophages were more abundant in patients with *MYCN* amplification, whereas *THY1*^+^ and *IL18*^+^ macrophages were fewer (Extended Data Fig. 9a). Additionally, proliferating macrophages were enriched in patients with advanced-stage disease (Extended Data Fig. 9b). Collectively, diverse macrophage subsets coexist and correlate with genetic and clinical features. Longitudinally, pro-tumorigenic states with immunosuppressive, angiogenic or metabolic potential were expanded, whereas pro-inflammatory populations were concomitantly reduced.

### Macrophages differentially interact with neoplastic cell states

Next, we explored how these macrophage subtypes interact with neoplastic cells by performing a cell–cell interaction analysis using CytoTalk^42^. We identified numerous bidirectional ligand–receptor interactions (Fig. 4e and Extended Data Fig. 9c). Many of these interactions involved proteins that facilitate cell adhesion, cell migration and angiogenesis, including the ligands VCAN (with the receptors ITGB1 and EGFR), THBS1 (with the receptors ITGB1, LRP5, ITGA3, CD47 and ITG2B), VEGFA (with the receptor GPC) and SEMA3A (with the receptor NRP1). We found that interactions between epidermal growth factor family (ErbB) receptors (ERBB4 and EGFR) and multiple ligands (HB-EGF, TGFA, EREG, AREG and ICAM1) constituted the most frequently enriched signaling pathway. Notably, these interactions were preferentially predicted between *VCAN*^+^ macrophages and all neoplastic populations (Fig. 4e and Extended Data Fig. 9d,e). The *THY1*^+^ macrophages were involved in interactions related to collagen and integrin signaling, whereas the *HS3ST2*^+^ macrophages expressed ligands related to lipid metabolism (for example, APOE, LRP5 and LPL), interacting with multiple neoplastic cell states (Fig. 4e and Extended Data Fig. 9c).

**Extended Data Fig. 10:**
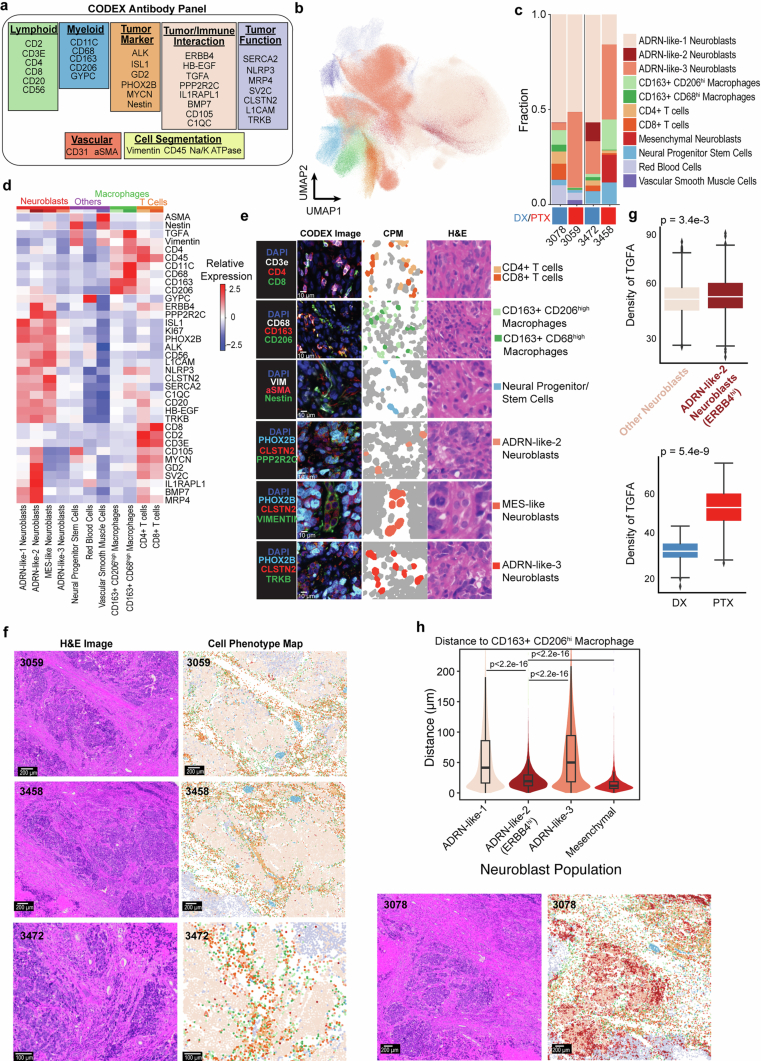
Annotation and protein expression in CODEX spatial imaging. **a**) List of CODEX panel markers for different cell types. **b**) UMAP visualization of the ~841,000 cells in CODEX data. **c**) Stacked barplot of cell type proportions across samples in the CODEX dataset. **d)** Heatmap of normalized protein expression across cell types in CODEX data. **e)** Representative images of CODEX immunofluorescence, cell phenotype mask (CPM), and hematoxylin and eosin (H&E) stain for selected cell types. **f)** Hematoxylin and eosin **(**H&E) image (left) and cell phenotype mask (right) of representative images for each CODEX sample. **g)** Comparison of the density of TGFA protein quantified by CODEX between neighbors of ADRN-like-2 (ERBB4^hi^) neuroblasts and the neighbors of other neuroblasts (top), and between diagnosis and post-therapy samples (bottom). The density was defined as the mean expression of TGFA on cells within a 40 µm square, excluding the marker within the center cell. Significance was assessed using a two-sided Wilcoxon rank-sum test. The numbers of cells are: n=655,573 and 17,532 (top); n = 221,677 and 451,428 (bottom). **h**) Distance from each neuroblast cell to the nearest CD163^+^CD206^hi^ macrophage across samples, stratified by neuroblast population. Significance was assessed using a two-sided Wilcoxon rank-sum test. Outliers were truncated for visualization purposes. Source data

To validate the intercellular interactions predicted via snRNA-seq, we performed co-detection by indexing (CODEX) spatial proteomics using a 38-antibody panel on whole-slide formalin-fixed, paraffin-embedded (FFPE) samples from two diagnostic–post-therapy pairs included in the single-cell transcriptomic atlas (Supplementary Table 11 and Extended Data Fig. 10a). After single-cell segmentation and clustering, we resolved the major cell lineages, including neuroblasts, macrophages and T cells, and discerned multiple distinct neuroblasts and macrophage subsets (Extended Data Fig. 10b–f). We found three subsets of adrenergic neuroblasts: an ISL1-high population (ADRN-like-1), a PPP2R2C-high population (ADRN-like-2) and a population with minimal PHOX2B expression (ADRN-like-3). We also identified a mesenchymal neuroblastic population expressing vimentin (Extended Data Fig. 10d,e). Notably, the immunotherapy target GD2 was exclusively expressed on ADRN-like-2 neuroblasts. Finally, we found that all macrophages expressed CD163, but two populations were discriminated by the additional high expression of either CD206 or CD68 (Extended Data Fig. 10d), resembling *F13A1*^+^ and *C1QC*^+^*SPP1*^+^ macrophages in our transcriptomic data (Fig. 4b).

Focusing on the epidermal growth factor-related pathways, we first observed that the ERBB4 receptor was most highly expressed on ADRN-like-2 neuroblasts (Extended Data Fig. 10d). We then examined the ligand signal density on cells within a 40-µm radius surrounding each neuroblastic cell. The densities of the secreted ERBB4 ligands HB-EGF and TGFA were greater in the vicinity of ADRN-like-2 (ERBB4^hi^) cells compared with the other neuroblasts and both ligands were broadly enriched after therapy (Fig. 4f–h and Extended Data Fig. 10g). We also found that the ADRN-like-2 (ERBB4^hi^) population was proximal to both macrophage subsets and significantly closer to CD163^+^CD68^hi^ macrophages than the other neuroblasts (Fig. 4i and Extended Data Fig. 10h). These results demonstrate the ability to spatially resolve neoplastic and immune cells and support macrophage-induced ErbB signaling as a potential pro-tumorigenic mechanism driving adrenergic neoplastic cells.

### Murine model recapitulates tumor–macrophage interactions

**Fig. 5:**
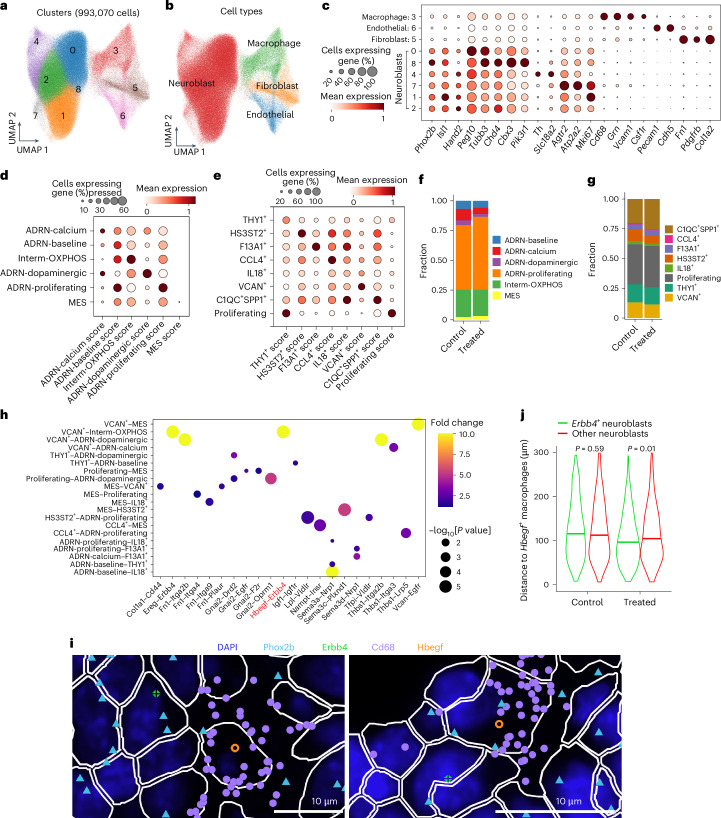
Spatial transcriptomic analysis of murine neuroblastoma. **a**,**b**, UMAP projection of Xenium transcriptomic data (993,070 cells) annotated by cell cluster (**a**) and major cell type (**b**). **c**, Dotplot showing the normalized expression levels of marker genes and the percentages of cells expressing them for each annotated cell type. Each row represents a cell cluster, with the average gene expression for each cluster normalized to a range between 0 and 1 across clusters. **d**,**e**, Dotplots showing the normalized signature scores for each neoplastic cell state (**d**) and macrophage subset (**e**). Each row represents a predicted cell subpopulation, with the average signature score for each cell state normalized to a range between 0 and 1 across cell types. **f**,**g**, Barplots displaying the proportions of projected neoplastic cell states (**f**) and macrophage subsets (**g**) for treated mice and controls. **h**, Spatial co-localization analysis of ligand–receptor interactions predicted between neoplastic cells and macrophage subsets based on snRNA-seq data. The Hbegf–Erbb4 interaction is highlighted in red. **i**, Representative image illustrating the spatial co-localization of *Erbb4*^+^ neuroblasts and *Hbegf*^+^ macrophages. The dots represent individual transcripts. **j**, Comparison of spatial distances between *Hbegf*^+^ macrophages and *Erbb4*^+^ neuroblasts versus other neuroblasts. Significance was assessed by two-sided (left) and one-sided (right) Wilcoxon rank-sum test. Source data

To further investigate the predicted interactions between macrophages and neuroblasts in vivo, we utilized Xenium spatial transcriptomics to study tumors from the well-validated immunocompetent TH-MYCN mouse model^43^, genetically engineered to overexpress *MYCN* in the murine neural crest, resulting in spontaneous tumors. We profiled treatment-naive and cyclophosphamide-treated mice with a panel of 5,000 genes and captured all of the major cell types identified in our multiomic cohort, including cells resembling all neoplastic and macrophage phenotypes (Fig. 5a–g). We then applied a spatial ligand–receptor analysis (Supplementary Methods), which validated a broad range of the interactions predicted by snRNA-seq. This included the HB-EGF–ERBB4 interaction, which occurred predominantly between *VCAN*^+^ macrophages and Interm-OXPHOS neoplastic cells in the mouse model, consistent with the predicted interactions (Fig. 4e and Fig. 5h). We also observed that *Erbb4*^+^ neuroblasts are spatially closer to *Hbegf*^+^ macrophages than other neuroblasts in the cyclophosphamide-treated, but not treatment-naive, mice (Fig. 5i,j). Overall, these results demonstrate that the key microenvironmental phenotypes and cellular interactions in human neuroblastoma are also present in a well-validated mouse model, providing a basis for the preclinical testing of therapeutic strategies that module the tumor-immune microenvironment.

### Macrophage-secreted HB-EGF promotes tumor survival

**Fig. 6:**
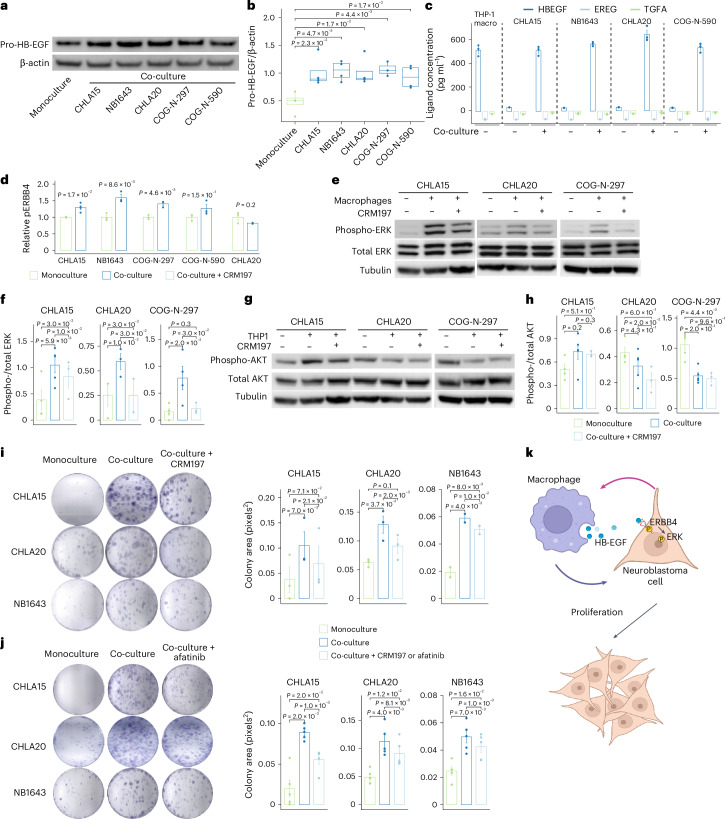
Macrophage-secreted HB-EGF activates ERK signaling and promotes proliferation. **a**, Representative western blot of cell-surface HB-EGF (pro-HB-EGF) from THP-1 macrophages in monoculture and co-culture with neuroblastoma cell lines. **b**, Quantification of the results from **a** across all replicates, normalized to β-actin (*n* = 3 for COG-N-297 and *n* = 4 for the others). Central lines indicate median values, the box edges mark the 25th and 75th percentiles and the whiskers extend 1.5 times the interquartile range. **c**, Ligand concentrations in the media of THP-1 macrophage monoculture and co-culture with neuroblastoma cell lines, measured by ELISA (*n* = 3). **d**, Phosphorylated ERBB4 (pERBB4) levels in neuroblastoma cells after co-culture with THP-1 macrophages, measured by ELISA (*n* = 4 for CHLA15 and *n* = 3 for the others). Significance in **b** and **d** was calculated using a Welch’s two-sided *t*-test. **e**, Representative western blots showing ERK activation in neuroblastoma cells with and without macrophage co-culture and the HB-EGF inhibitor CRM197. **f**, Quantification of the results from **e** across all replicates, normalized to total ERK (*n* = 5, 3 and 4, from left to right). **g**, Representative western blots showing AKT phosphorylation in neuroblastoma cells with and without macrophage co-culture and the HB-EGF inhibitor CRM197. **h**, Quantification of the results from **g** across replicates, normalized to total AKT (*n* = 3 for the treatment group and *n* = 5 for the others). **i**, Representative images (left) and quantification (right) of the area of colony formation for neuroblastoma cells co-cultured with THP-1 macrophages with or without treatment with CRM197 (from left to right: *n* = 3, 3 and 2 per condition). **j**, Representative images (left) and quantification (right) of the area of colony formation for neuroblastoma cells co-cultured with THP-1 macrophages with or without treatment with the pan-ERBB inhibitor afatinib (*n* = 4). The experiments in **i** and **j** were repeated two to four times with two to four samples per condition and the averaged values across samples are shown. Significance was calculated using a one-sided paired *t*-test (**f**, **i** and **j**) or two-sided paired *t*-test (**h**). The error bars in **a**–**c**, **f** and **h**–**j** represent means ± s.d. **k**, Neuroblastoma cells stimulate HB-EGF secretion from THP-1-derived macrophages, which reciprocally induce the phosphorylation of ERBB4 on neuroblastoma cells. Activation of ERBB4 stimulates proliferation via the ERK pathway. Panel **k** was created with BioRender.com. Source data

Given these ligand–receptor interactions, we hypothesized that pro-tumorigenic macrophages contribute to therapeutic resistance via activation of ErbB receptor tyrosine kinases in adrenergic neoplastic states. We nominated several ligands of the ERBB4 growth factor receptor, including HB-EGF, EREG and TGFA. Moreover, HB-EGF and EREG consistently interacted with ERBB4 in all neoplastic states except the MES state (Fig. 4e). We found that *HBEGF*, but not *EREG* or *TGFA*, was expressed more after therapy across the snRNA-seq dataset within multiple macrophage subsets (Extended Data Fig. 9e). Therefore, we aimed to examine the coordinated roles of HB-EGF and the ErbB pathway in vitro by co-culturing five neuroblastoma cell lines with macrophages differentiated from the THP-1 monocyte cell line (THP-1 macrophages). First, we found that the surface expression of HB-EGF on THP-1 macrophages was increased when co-cultured with neuroblastoma cells in all five cell lines (Fig. 6a,b). Furthermore, an enzyme-linked immunosorbent assay (ELISA) performed with cell culture supernatant confirmed the exclusive secretion of HB-EGF ligand by THP-1 macrophages in the co-culture and the absence of EREG and TGFA expressions (Fig. 6c), further nominating HB-EGF as the active ligand. In parallel, we noticed significant phosphorylation increase of the ERBB4 receptor on neuroblastoma cells when co-cultured with macrophages, compared with monoculture, in three of the five cell lines examined (Fig. 6d). Taken together, these findings implicate that HB-EGF/ERBB4 signaling mediates the neuroblast and macrophage interaction.

ErbB signaling has been demonstrated to regulate cell proliferation, differentiation and apoptosis through the PI3K–AKT and RAS–RAF–MEK–ERK pathways^44^. Therefore, we aimed to determine which downstream signaling pathways are induced by ErbB activation. We performed western blots of total and phosphorylated AKT and ERK proteins after co-culture and monoculture using three neuroblastoma cell lines. We found that ERK phosphorylation was increased whereas AKT phosphorylation was unchanged or decreased in all three cell lines. Moreover, in the presence of the HB-EGF inhibitor CRM197, ERK phosphorylation was significantly reduced whereas AKT phosphorylation remained unchanged, suggesting that ERK activation is indeed induced by HB-EGF secreted from co-cultured macrophages (Fig. 6e–h).

We hypothesized that the activated ErbB pathway contributes to tumor cell survival. To test this, we treated three neuroblastoma cell lines co-cultured with THP-1 macrophages either with CRM197 or the ERBB tyrosine kinase inhibitor afatinib. We quantified the resulting growth effect using a colony formation assay before and after pharmacological inhibition. We first confirmed that neuroblastoma cell growth was increased with macrophage co-culture compared with monoculture in all three cell lines tested. Next, we found that neuroblastoma cell growth was consistently reduced by either CRM197 or afatinib treatment (Fig. 6i,j). In summary, we conclude that neuroblastoma cells induce HB-EGF in TAMs, which in turn activates the ERBB4 receptor in tumor cells, stimulates downstream ERK signaling and promotes tumor cell proliferation (Fig. 6k).

**Fig. 7:**
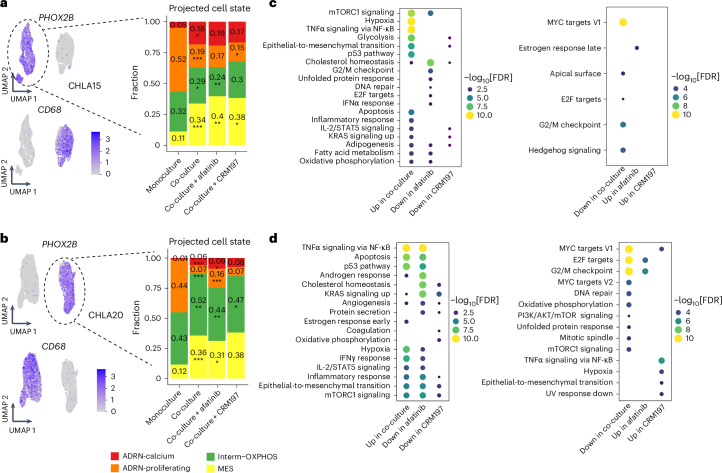
Transcriptomic analysis of mono- and co-cultured macrophages and neuroblasts. **a**, Left, UMAP plots showing cells from monoculture and co-culture with THP-1 macrophages, colored by the normalized expression levels of *PHOX2B* and *CD68* for the CHLA15 cell line. Right, barplots depicting the proportions of projected neoplastic cell states across experimental conditions. **b**, UMAP and neoplastic cell state inference of co-culture experiments with the CHLA20 cell line as in **a**. The *P* values in **a** and **b** were calculated using a two-sided proportion test, comparing either co-culture versus monoculture or co-culture with treatment versus co-culture without treatment. No multiple comparison adjustment was made. **c**, Differential pathway analysis of CHLA15 cells using the hallmark pathways from the Molecular Signatures Database. Pathways in the co-culture condition were compared versus the monoculture condition and pathways in the co-culture with treatment condition were compared versus the co-culture without treatment condition. **d**, Pathway analysis of co-cultured CHLA20 cells as in **c**. The *P* values in **c** and **d** were calculated based on Fisher’s exact test using enrichR and corrected using the Benjamini–Hochberg procedure. *P < 0.1; **P < 0.01; ***P < 0.001. FDR, false discovery rate; IFN, interferon; IL-2, interleukin 2; mTORC1, mammalian target of rapamycin complex 1; TNF, tumor necrosis factor. Source data

To assess the broad impact of tumor–macrophage interactions on neoplastic cell phenotypes, we performed scRNA-seq on the neuroblastoma cell lines CHLA15 (diagnostic) and CHLA20 (after therapy) co-cultured with THP-1 macrophages. Co-culture induced notable changes in neuroblast cell states. In both cell lines, the ADRN-calcium, Interm-OXPHOS and MES states expanded upon co-culture, whereas the ADRN-calcium state retracted (Fig. 7a,b), mirroring the cell state shifts that occur after standard therapy (Fig. 2e). Subsequently, treatment with afatinib consistently led to a decrease in the Interm-OXPHOS state. A pathway analysis of the tumor cells revealed that co-culture upregulated multiple pathways, including epithelial-to-mesenchymal transition, tumor necrosis factor alpha signaling and inflammatory response, and downregulated cell cycle pathways (Fig. 7c,d and Supplementary Table 12). Treatment with afatinib or CRM197 reversed many of these pathways compared with the untreated co-culture condition. For example, epithelial-to-mesenchymal transition was suppressed by CRM197 in both cell lines after its initial upregulation upon co-culture (Fig. 7c,d). This experiment suggests that macrophage interaction induces similar neoplastic state shifts that occur during standard therapy and implicates macrophage-driven ErbB signaling in modulating various neoplastic cellular processes.

## Discussion

We present a single-cell multiomic analysis of patient-matched longitudinal high-risk neuroblastoma. Through high-resolution transcriptomic and epigenomic profiling of paired untreated and induction chemotherapy-treated specimens, we uncovered diverse tumor and immune phenotypes and revealed profound shifts in the tumor microenvironment during standard therapy.

We identified an adrenergic-to-mesenchymal phenotypic spectrum of neoplastic cells. Moreover, we found that the proportion of proliferating adrenergic cells decreased after therapy, whereas more differentiated neuronal-like cells expanded. Mesenchymal neoplastic cells, resembling mixed developmental lineages, showed a slight increase after therapy. A higher mesenchymal state at diagnosis did not predict worse survival, but correlated with poorer chemotherapy response. In contrast, a larger fraction of proliferating and metabolically active neoplastic cells at diagnosis predicted a worse clinical outcome, highlighting the need for combinatorial therapies. Our analysis also uncovered important regulators, such as MAZ and CTCF, for the high-risk proliferating state to maintain its persistent transcriptomic program.

Beyond neoplastic cells, we identified a substantial increase in the macrophage population after therapy shifting towards pro-tumorigenic phenotypes. Particularly, we found that HB-EGF was upregulated in macrophages and interacted with all neoplastic states. HB-EGF is a critical factor in multiorgan development and tissue remodeling^45^ and has been implicated in tissue response to injury and metastatic progression^46–48^. In neuroblastoma, the ErbB tyrosine kinases, such as EGFR and ERBB4, have been reported to promote cell growth and prevent apoptosis in preclinical models via the MAPK–ERK and PI3K–AKT^49–51^ pathways. Using multiple neuroblastoma cell lines, we found that tumor cells induce macrophage HB-EGF secretion, which in turn promotes neuroblast proliferation through activation of the ERK pathway. Of note, our analysis also identified HB-EGF–EGFR signaling between mesenchymal neoplastic cells and macrophages; however, our experiment could not rule out the contribution of EGFR for ERK activation. Future studies are expected to clarify this mechanism and to assess differential responses of neoplastic states to HB-EGF signaling.

In summary, our study sheds light on the molecular mechanisms of therapeutic resistance in high-risk neuroblastoma and provides a valuable resource for further analytical inquiry.

## Methods

### Human biospecimens and ethical approval

Primary patient samples were obtained from the Children’s Hospital of Philadelphia Center for Childhood Cancer Research Biobank. Biospecimens were obtained with informed consent from parents according to the Declaration of Helsinki and Institutional Review Board approval. All patient data were deidentified and written informed consent was obtained to publish the indirect identifiers in the present manuscript. Patient sample information and relevant clinical metadata are provided in Supplementary Table 1.

### snRNA-seq

Single-nucleus suspensions immediately underwent library preparation following the 10x Genomics protocol using a Chromium Controller with Chromium Single Cell 3′ Reagent Kit V3 or V3.1, per the manufacturer’s instructions. Library quality was assessed using an Agilent 2100 Bioanalyzer with a High Sensitivity DNA chip (5067-4626; Agilent Technologies). Indexed libraries were pooled and sequenced on an Illumina NovaSeq 6000 using the sequencing parameters 28:8:0:87 (read1:i5:i7:read2, bp) with an average sequencing depth of 50,000 read pairs per nucleus.

### snATAC-seq

Single-nucleus suspensions immediately underwent library preparation following the 10x Genomics protocol using a Chromium Controller with Chromium Next GEM Single Cell ATAC Reagent kit V1 or V1.1, per the manufacturer’s user manual. Library quality was assessed using an Agilent 2100 Bioanalyzer with a High Sensitivity DNA chip (5067-4626; Agilent Technologies). Indexed libraries were pooled and sequenced on an Illumina NovaSeq 6000 using the sequencing parameters 49:8:16:49 (read1:i5:i7:read2, bp) with an average sequencing depth of 50,000 read pairs per nucleus.

### CODEX antibody staining

CODEX staining was performed using a Sample Kit for PhenoCycler-Fusion (7000017; Akoya) according to Akoya’s PhenoCycler-Fusion user guide, with modifications to include a photobleaching step and overnight incubation with antibodies at 4 °C. FFPE samples were sectioned at 5-μm thickness and mounted onto charged slides (3800080; Leica) by the Pathology Core at the Children’s Hospital of Philadelphia. Sample slides were baked overnight at 65 °C for 3 h and allowed to cool to room temperature. They were then deparaffinized in HistoChoice clearing agent (H103-4L; VWR) twice and rehydrated in a graded series of ethanol concentrations (twice in 100, 90, 70, 50 and 30% and twice in ddH_2_O). Antigen retrieval was performed in 1× citrate buffer (C9999; Sigma–Aldrich) in a pressure cooker for 20 min. After equilibrating to room temperature, sample slides were washed twice with ddH_2_O and once with 1× Dulbecco's phosphate-buffered saline (DPBS) before being submerged in a petri dish containing 4.5% H_2_O_2_ and 20 mM NaOH in 1× DPBS (bleaching solution) for photobleaching. The petri dish was sandwiched between two broad-spectrum LED light sources for 45 min at 4 °C. After 45 min, sample slides were transferred to a new petri dish with freshly made bleaching solution and photobleached for another 45 min at 4 °C. Sample slides were washed three times in 1× DPBS and then twice in hydration buffer. Sample slides were equilibrated in staining buffer for 30 min and incubated in the antibodies (Supplementary Table 9) diluted in staining buffer plus N Blocker, G Blocker, J Blocker and S Blocker overnight at 4 °C. After antibody incubation, sample slides were washed twice in Staining Buffer and fixed for 10 min in 1.6% paraformaldehyde (15710; Electron Microscopy Sciences) in storage buffer. Sample slides were washed three times in 1× DPBS and incubated in ice-cold methanol for 5 min. After incubation in methanol, sample slides were washed three times in 1× DPBS and incubated in a final fixative solution (1 ml 1× DPBS + 20 µl Akoya’s final fixation reagent) for 20 min at room temperature. The sample slides were then washed three times in 1× DPBS and stored in storage buffer before imaging.

### CODEX imaging

CODEX reporters were prepared according to Akoya’s PhenoCycler-Fusion user guide and added to a black 96-well plate. The PhenoCycler-Fusion experimental template was set up for a CODEX run using Akoya’s PhenoCycler Experiment Designer software according to Akoya’s PhenoCycler-Fusion user guide. Details on the order of fluorescent CODEX barcodes and microscope exposure times are provided in Supplementary Table 9. The PhenoCycler-Fusion experimental run was performed using Akoya’s Fusion 1.0.8 software according to Akoya’s PhenoImager Fusion user guide. Images were taken and preprocessed (stitching, registration and background subtraction) with Akoya’s PhenoImager Fusion microscope using the default settings. Final images were evaluated, then selected samples were reimaged with adjusted exposure times based on manual review. After imaging, slides were stained with hematoxylin and eosin. Akoya’s Fusion 1.0.8 software was used to image hematoxylin and eosin-stained slides at 20× resolution in brightfield on the PhenoCycler-Fusion system.

### Monocyte differentiation and co-culture with neuroblastoma cells

To obtain macrophages, 10^6^ THP-1 monocytes in 4 ml complete media were seeded in 0.4 μm polyethylene cell inserts for six-well plates (930-04-12; cellQART) and treated with 100 ng ml^−1^ phorbol 12-myristate 13-acetate (P1585; Sigma–Aldrich) in complete media for 72 h. After detaching the neuroblastoma cells with versene solution (0.02% EDTA in Hank’s Balanced Salt Solution), they were plated in six-well plates in complete media overnight and allowed to reach 70–75% confluence at the time of co-culture. Co-culture media was prepared by mixing RPMI 1640 media plus 1% fetal bovine serum (FBS) and Iscove's Modified Dulbecco's Medium (IMDM) media plus 1% insulin- transferrin-selenium (ITS) at a 1:1 ratio. THP-1 macrophages in the cell insert were washed twice with Dulbecco’s phosphate-buffered saline (DPBS) (14190144; Thermo Fisher Scientific) and transferred to neuroblastoma cell culture plates. Neuroblastoma cells and macrophages were co-cultured in 6 ml co-culture media. Neuroblastoma cells cultured without an insert and THP-1 macrophages in inserts within empty plates were used as control monocultures. To inhibit HB-EGF activity, 2 μg ml^−1^ CRM197 (23218; Cayman) was added to the co-culture media. After 48 h, monocultured and co-cultured macrophages and neuroblastoma cells with or without CRM197 were harvested for further analysis.

### ERBB ligand profiling

THP-1-derived macrophages were plated and differentiated on inserts for six-well plates for 72 h. Neuroblastoma cells were plated on six-well plates in complete media. Then, neuroblastoma cells and THP-1 macrophages were co-cultured with 4 ml co-culture media (0.5% FBS-supplemented RPMI:IMDM mixture) for 48 h. Cell culture supernatants were collected, centrifuged at 500*g* for 5 min and filtered with a 0.22-µm polyethersulfone membrane. Supernatants were aliquoted into microcentrifuge tubes and stored at −80 °C for ELISA. The secreted form of ERBB receptor ligands was assessed by performing ELISAs for HB-EGF (Quantikine (DHBEG0; R&D Systems)), TGFA (Quantikine (DTGA00; R&D Systems)) and EREG (Epiregulin BioAssay ELISA kit (382783; USBiological Life Sciences)) according to the manufacturers’ protocols. Absorbance at 450 nm was measured using a FLUOstar Omega-BMG LABTECH' microplate reader. The absorbance signal from co-culture media was utilized for background correction. The standard curve was constructed by plotting the standards and used to calculate the ligand concentration in supernatant. Three biological replicates were used for each cell line and treatment condition.

### Colony formation assay

THP-1 cells were plated onto 0.4-µm polyethylene clear cell inserts for 24-well plates (9320412; cellQART) at 2 × 10^5^ cells per well in 500 µl complete media supplemented with 100 ng ml^−1^ phorbol 12-myristate 13-acetate and incubated for 72 h. Neuroblastoma cells were plated onto 24-well plates at a density of 1,000 cells per well (CHLA15 and NB1643) or 500 cells per well (CHLA20) in 1 ml co-culture media (2% FBS-supplemented RPMI and IMDM mixture plus1% insulin-transferrin-selenium). After washing the THP-1 macrophages on the inserts with DPBS twice, they were transferred to neuroblastoma plates with the addition of 500 µl co-culture media. Macrophages and neuroblastoma cells were co-cultured for 7 d, refreshing the media on day 4. Macrophages on inserts were discarded and neuroblastoma cells were fixed with 4% paraformaldehyde (15710-S; Electron Microscopy Sciences) for 20 min at room temperature. After permeabilization with 0.3% Triton X-100 (T8532; Sigma–Aldrich) in DPBS for 10 min, cells were stained with 0.5% crystal violet (V5265; Sigma–Aldrich) for 3 h at room temperature. Plates were air-dried, followed by the removal of excess stain with Milli-Q water. To examine the role of the ErbB pathway on colony formation, 100 nM afatinib (S1011; Selleck Chemicals) or 4 µg ml^−1^ CRM197 was added to the co-culture media. Neuroblastoma cells without an insert served as monoculture controls. Images of colonies were obtained by scanning the whole plate with an at the highest resolution and analyzed with ImageJ as previously described^52^. Briefly, the following steps were applied to obtain the total colony area and average colony size: (1) enhance the local contrast; (2) make binary; (3) apply Gaussian blur with a two-pixel radius; (4) make binary; (5) watershed; (6) select the target well in the plate; (6) set the measurements area and area fraction; and (7) analyze the particles.

### snRNA-seq data processing and integration

Raw reads were aligned to the Genome Reference Consortium Human Build 38 patch release 13 (GRCh38.p13) assembly and quantified among the genes using Cell Ranger version 3.1.0. High-quality cells were maintained if their unique molecular identifier count was between 2,000 and 40,000 and they expressed between 1,000 and 10,000 genes and <10% of unique molecular identifiers mapped to mitochondrial genes. The filtered cells were normalized with log normalization using Seurat version 3 (ref. ^53^). The doublets identified with DoubletFinder^54^ with default parameters were also removed. Reads from ambient RNA were removed from the raw counts using decontX^55^ and the decontaminated matrices were rounded up to the nearest integer and renormalized with log normalization using Seurat version 3.

The normalized expression matrices were integrated using reciprocal principal component analysis (PCA) implemented in Seurat version 3. Specifically, the Seurat object with all cells was first split by patient identifier and the top 2,000 highly variable genes were identified using the SelectIntegrationFeatures function in Seurat. We excluded genes that were highly patient specific from the highly variable genes by aggregating pseudo-bulk count matrices across patients. We then filtered out genes with a Gini index value of >0.8. Genes expressed in <0.2% of cells within all patients were further removed from the highly variable gene list. Cells were subsequently integrated using the FindIntegrationAnchors function in Seurat, with the parameter k.anchor set to 10. The integrated data were then scaled for PCA. Uniform manifold approximation and projection (UMAP) embeddings were computed using the first 50 PCA dimensions for visualization. Louvain clustering was run using the first 50 principal components with the resolution parameter set to 0.2. The cell types of clusters were manually assigned using marker genes.

### Integration of neoplastic cells in snRNA-seq data

Putative neoplastic cells were first pooled and processed using the standard Seurat pipeline, as implemented above with minor modifications. Briefly, the top 2,500 highly variable genes were selected using the FindVariableFeatures function. We also excluded genes that were highly patient specific from the highly variable genes by aggregating pseudo-bulk count matrices across patients and then filtered out genes with a Gini index value of >0.8. The data were scaled to compute PCA embeddings. UMAP embeddings and Louvain clustering with a resolution of 0.2 were conducted based on the first 30 principal components. The clusters mostly contained cells from a single patient, except cluster 33 (1,804 cells), which contained a mixture of cells from different patients. This cluster of cells was therefore suspected to be non-neoplastic and removed from subsequent analysis. We then integrated all of the malignant cells using Harmony^56^ along with the patient identifier. The UMAP embeddings and Louvain clustering were computed on the first 20 components of the Harmony-derived dimensional reduction with a resolution of 0.2.

### Integration and annotation of macrophage subsets in snRNA-seq data

Macrophages were extracted from the full snRNA-seq data object and processed using the standard Seurat pipeline, as described above, and reintegrated with Harmony using the same protocol as for the malignant cells. Cells were clustered in Seurat based on the first 20 components of the Harmony-derived dimensional reduction using a resolution of 0.4. Cell clusters with >5% of cells previously predicted to be malignant by artificial neural network classifier were filtered out for downstream analysis. The remainder were reprocessed using the same Seurat pipeline and Harmony integration. Briefly, the top 1,000 highly variable genes were used to scale the data and calculate PCA embeddings. Harmony integration was then performed on the first 20 principal components along each patient identifier. Subsequently, all macrophages were clustered and UMAP embeddings were calculated based on the first 20 components of the Harmony-derived dimensional reduction with a resolution of 0.4. Differentially expressed genes were calculated for each population using the FindAllMarkers function with the parameters max.cells.per.ident = 500, min.pct = 0.05 and min.diff.pct = 0.05. Each macrophage was manually annotated based on significant differentially expressed genes^41^. Cells identified as dendritic cells (cluster 6; Extended Data Fig. 6a) were removed before downstream analysis. No discrete monocyte cluster was observed, so the remaining cells were subsequently annotated as TAM subsets.

### snATAC-seq data processing, integration and cell type annotation

snATAC-seq data for each sample were preprocessed using Cell Ranger ATAC version 1.1.0 (10x Genomics) to generate FASTQ files, which were then processed using the process module of scATAC-pro^57^ (version 1.5.1) with the default parameters. We aligned the raw reads to the GRCh38.p13 assembly using the Burrows–Wheeler Aligner (version 0.7.17)^58^. Peaks were called using MACS2 (ref. ^59^). We defined high-quality cells to have between 5,000 and 100,000 total fragments, <15% mitochondrial reads and a >25% fraction of reads in peaks. The peak-by-cell count matrix was constructed and used for downstream analyses. To integrate data from all patients, we first merged the peaks from different samples if two peaks were within 500 base pairs of each other using the mergePeaks module of scATAC-pro. The peak-by-cell count matrix was then reconstructed based on the merged peaks using the reConstMtx module of scATAC-pro. We pooled matrices from all samples and loaded them into Seurat with an extra ChromatinAssay added. The data were then integrated using Signac^60^ as follows. The Seurat object was split by patient identifier and each patient subset was then normalized using the RunTFIDF function. Top features were identified using FindTopFeatures with the min.cutoff parameter set to 1% of the number of cells present in the subset and a singular value decomposition was computed using the RunSVD function. Data were then integrated using the FindIntegrationAnchors function with the parameters reduction = rlsi, anchor.features = 10000, k.anchor = 30 and dims = 2:50, followed by the IntegrateEmbedding function with the parameters k.anchor = 30 and dims = 2:50. A UMAP embedding was constructed and the cells were clustered with Louvain clustering (at a resolution of 0.4) using the integrated latent semantic indexing reduction and dimensions 2–50.

Cells in the integrated snATAC-seq were annotated using the Seurat label transfer pipeline. Briefly, the integrated and annotated snRNA-seq data were used as the reference. Gene activity scores were calculated on the snATAC-seq data using the GeneActivity function in Signac. Subsequently, we normalized the gene activity matrix using Seurat log normalization. We employed the FindTransferAnchors function with the parameters reduction = cca and k.anchor = 20, followed by the TransferData function with the parameters dims = 2:50 and k.weight = 50 in Seurat to predict the cell types for each cell in the snATAC-seq data. Each cell cluster was then annotated as the cell type most frequently predicted within the cluster.

### Neoplastic cell state identification in snATAC-seq data

To understand the chromatin state of each neoplastic cell state identified via snRNA-seq, we identified the putative malignant cells in snATAC-seq as follows. All snATAC-seq cells were initially pooled and processed using the Seurat/Signac pipeline as described but without integration. After clustering and visualization on a UMAP projection, all cells within patient-specific clusters (>90% cells from a single patient) previously annotated as neuroblasts, fibroblasts or Schwann cells in the integrated snATAC-seq data were defined as putative neoplastic cells. Then, we reintegrated the putative neoplastic cells using the Signac pipeline, as described above, with the exception that Louvain clustering was performed using dimensions 2–30 of the integrated latent semantic indexing embedding and a resolution of 0.2. Lastly, to map individual cells in the snATAC-seq data to the transcriptionally defined cell states, we applied the Seurat label transfer pipeline. We employed the FindTransferAnchors function with the parameters reduction = cca and k.anchor = 15, followed by the TransferData function with the parameters dims = 2:30 and k.weight = 50. Cells with a maximum prediction score of <0.6 were removed from downstream analysis.

### Transcriptional regulatory network analysis

The transcriptional regulatory network for each neoplastic cell state was constructed as described previously^61,62^ with minor modifications. We first co-embedded the malignant cells in snATAC-seq and snRNA-seq data per sample using the Seurat multimodality co-embedding pipeline. Each snATAC- and snRNA-seq sample was processed separately, followed by application of the FindTransferAnchors function with the parameters reduction = cca and k.anchor = 30, followed by the TransferData function with default parameters. Then, we identified metacells using the R package hdWGCNA^63^ with the parameters k = 25, max_shared = 3, min_cells = 100, reduction = pca and ident.group = seurat_clusters. Metacells containing between 5 and 15 snRNA-seq cells were retained for further analysis. The gene-by-metacell expression matrix and peak-by-metacell accessibility matrix were calculated as the average normalized expression and normalized accessibility of all cells within the metacell, respectively. Metacells from different samples were then combined and the enhancer–promoter interactions were predicted using a linear regression model for each gene on metacells, with the gene expression in each metacell as the dependent variable and the accessibility of the peaks within ±500 kilobases of the gene promoter as the independent variables. Significant enhancer–promoter interactions were defined based on a peak regression coefficient of >0.2 and a Benjamini–Hochberg-adjusted *P* value of <0.01. Transcription factor–target gene pairs were defined if the transcription factor motif was present at the enhancer of a predicted enhancer–promoter interaction. To obtain robust networks, only enhancer–promoter interactions with differential accessibility across neoplastic states in the enhancer peak were included. However, since we only obtained a few differentially accessible peaks for the Interm-OXPHOS state, instead of using differentially accessible peaks to filter enhancer peaks, we utilized all peaks accessible in >20% of Interm-OXPHOS cells to filter enhancer peaks for this state. Transcription factors were incorporated into the network if they were either differentially expressed or had differential motif activity and were expressed in at least 20% of the cells within a given cell state. Lastly, the target genes in a network were restricted to the differentially expressed genes in each cell state.

### Survival analysis

For survival analysis, we used bulk RNA-seq data generated for 498 patients with clinical information in the Sequencing Quality Control project^31^ study and 419 patients with clinical information in a dataset published by Cangelosi et al.^32^ downloaded from the R2 database. Overall and event-free survival data were retrieved from the published clinical annotations and processed with the Surv function in the R survival package^64^. We conducted two types of survival analyses. For the first type of analysis, the signature score of a neoplastic cell state was dichotomized into high and low categories as greater than or less than the median. A Cox proportional hazards regression model was fit to the survival object to access the significance of the dichotomized signature using the coxph function in the R survival package. For the second type of analysis, patients were grouped into different neoplastic cell states based on maximum cell state signature scores. Subsequently, a Cox proportional hazards regression model was fit to the survival object to access the significance of the cell state assignment, with the ADRN-calcium state as the baseline. In both cases, MYCN amplification event, sex and age were included as additional covariates in the model. Kaplan–Meier survival curves were generated using the ggsurvplot function in the R ggsurvfit package.

### Cell–cell interaction analysis

We computed the crosstalk between subsets of the neoplastic and macrophage cells using our recently developed method, CytoTalk^42^. This algorithm predicts functionally significant ligand–receptor interactions in single-cell sequencing data by analyzing both intercellular and intracellular gene networks downstream of receptor activation. For each pair of neoplastic and macrophage states, we first randomly sampled 5,000 cells from the neoplastic subset and 2,000 cells from the macrophage subset for each combination. Subsequently, we executed CytoTalk for all cell state pairs, restricting the analysis to genes expressed in at least 10% (default) of either cell state. The crosstalk scores were visualized as a dotplot. To evaluate the robustness of ErbB signaling, we employed two additional approaches, CellChat^65^ and LIANA^66^, to analyze interactions between all neoplastic and macrophage states, as well as other normal cell types. Each population was randomly downsampled to 5,000 cells when more than 5,000 cells were observed. For CellChat default parameters were applied, whereas for LIANA we set the parameter resource = all.

### CODEX data processing

Cell segmentation was conducted via Mesmer^67^ for each image. To generate the necessary input of a two-channel tag image file format (TIFF), we used DAPI for the nuclear channel and a fused channel of CD45, vimentin and NaK-ATPase for the membrane channel. The mean pixel intensity was extracted from each cell segmentation mask, yielding a cell-by-protein matrix that was carried forward for analysis in Scanpy^68^. Cells with a very low or high raw DAPI intensity (<10 or >250 on a UINT8 scale) were removed. Each image was manually cropped to exclude large areas of artifact including tissue folding and detachment, debris and edge artifact. Each sample was then internally normalized using the centered log ratio across all features, which is recommended for the analysis of protein expression, as in CITE-seq data. Samples were then merged into one object and integrated using Harmony^58^. Subsequently, scaling, PCA, UMAP and Leiden clustering were performed on this combined object. Each cluster was manually annotated based on the top differentially expressed proteins. CODEX data were visualized using QuPath version 0.4 and Napari version 0.4.18.

### Xenium spatial transcriptomics on TH-MYCN mice

A Xenium spatial transcriptomic experiment was performed on six TH-MYCN^+/+^ mice (Supplementary Methods). FFPE mouse tissue sections (5 µm) were prepared on Xenium slides using Xenium Prime Sample Preparation Reagents. Slides were deparaffinized, rehydrated and decrosslinked, followed by hybridization with Xenium 5K Mouse PTP Priming Oligos and probes targeting messenger RNA. Probes were ligated, amplified and washed, with subsequent antibody staining for cell segmentation. Autofluorescence quenching and nuclei staining were performed and slides the were stored in phosphate-buffered saline with tween-20 (PBS-T) at 4 °C. The Xenium Analyzer (version 3.1) was used for imaging, decoding, segmentation and cell assignment, following the manufacturer’s protocols.

### Statistics and reproducibility

No statistical method was used to predetermine sample size. All available longitudinal specimens at the Children’s Hospital of Philadelphia meeting the inclusion criteria were profiled and all data meeting the standard quality control threshold were included. The investigators were not blinded to allocation during genomics profiling and the assessment of patient data. Randomization and blinding were used for all of the in vitro experiments. A one-sided Wilcoxon signed-rank test for paired samples was used to compare the percentages of cell type proportions between patient-matched samples. As the Wilcoxon test is non-parametric, we did not formally test for normality of the data. Statistical analysis of sequencing and imaging data was conducted in R version 4.2. Analysis of in vitro data was conducted using the Welch’s one-sided paired or unpaired *t*-test in R, as indicated in the corresponding figure captions. The dose-response inhibition tool in GraphPad Prism was used to calculate drug half-maximum inhibitory concentration values. All representative images were replicated independently at least twice to ensure reproducibility. In all cases, box plots indicate median values, hinges mark the 25th and 75th percentiles and whiskers extend 1.5 times the interquartile range.

### Reporting summary

Further information on research design is available in the Nature Portfolio Reporting Summary linked to this article.

## Online content

Any methods, additional references, Nature Portfolio reporting summaries, source data, extended data, supplementary information, acknowledgements, peer review information; details of author contributions and competing interests; and statements of data and code availability are available at 10.1038/s41588-025-02158-6.

## Supplementary information

Supplementary Information Supplementary Methods.

Reporting Summary

Supplementary Tables Supplementary Tables 1–12.

## Source data

Source Data Fig. 1 Statistical source data.

Source Data Fig. 2 Statistical source data.

Source Data Fig. 3 Statistical source data.

Source Data Fig. 4 Statistical source data.

Source Data Fig. 5 Statistical source data.

Source Data Fig. 6 Statistical source data.

Source Data Fig. 6 Unprocessed gels or blots.

Source Data Fig. 7 Statistical source data.

Source Data Extended Data Fig. 1 Statistical source data.

Source Data Extended Data Fig. 2 Statistical source data.

Source Data Extended Data Fig. 3 Statistical source data.

Source Data Extended Data Fig. 4 Statistical source data.

Source Data Extended Data Fig. 5 Statistical source data.

Source Data Extended Data Fig. 6 Statistical source data.

Source Data Extended Data Fig. 7 Statistical source data.

Source Data Extended Data Fig. 8 Statistical source data.

Source Data Extended Data Fig. 9 Statistical source data.

Source Data Extended Data Fig. 10 Statistical source data.

## Data Availability

Data from this study have been deposited in the Human Tumor Atlas Network data portal (https://humantumoratlas.org/publications/hta4_2025_nature-genetics_wenbao-yu). For the snRNA-seq, snATAC-seq and WGS data, this includes sequencing reads and processed data (read alignments, gene-by-cell or peak-by-cell matrices and variant call files). We also deposited the processed snRNA-seq data in the CELLxGENE database at https://cellxgene.cziscience.com/collections/cee845e3-ec04-4781-9e2a-28734bb4f7ba for easy interactive exploration. For the CODEX data, this includes multi-channel images, segmentation masks and marker-by-cell matrices. For all data types, Seurat objects with annotations and dimensional reductions are provided. The linkage between Human Tumor Atlas Network patient IDs and sample IDs is provided in Supplementary Table 2. The processed Xenium transcriptomic and scRNA-seq data in the mono- and co-culture experiments have been deposited to the Zenodo repository at 10.5281/zenodo.14261274 (ref. ^69^). Source data are provided with this paper.

## Code availability

Source code created for this study is publicly available at https://github.com/tanlabcode/NBL_scMultiomics_Paper and ref. ^70^.
