# Supplementary material for: Longitudinal single-cell multiomic atlas of high-risk neuroblastoma reveals chemotherapy-induced tumor microenvironment rewiring
Source: Nat Genet. 2025 Apr 14;57(5):1142–54. doi: 10.1038/s41588-025-02158-6 (PMC12081299; doi:10.1038/s41588-025-02158-6)
Supplement: Supplementary file 1 — Supplementary Methods. [file 41588_2025_2158_MOESM1_ESM.pdf]

nature genetics

Supplementary information

https://doi.org/10.1038/s41588-025-02158-6

Longitudinal single-cell multiomic atlas of high-risk neuroblastoma reveals chemotherapy-induced tumor microenvironment rewiring

In the format provided by the authors and unedited

Supplemental Methods

Tumor Sample Preparation

Fresh frozen tumor samples were embedded in optimal cutting temperature (OCT) compound and cryosectioned into 40 µm tissue scrolls. Cryosections (4-6 scrolls) were dissociated using a 2-mL Dounce homogenizer (MilliporeSigma, D8938) with 1 mL ice-cold Nuclei Extraction Buffer (NEB, consisting of 1x PBS, 20mM Tris-HCl, 320mM sucrose, 5mM $CaCl_2$, 3mM $MgAc_2$, 0.1mM EDTA, 0.01% Digitonin and 0.1% TrionX-100). The tissue was homogenized 10 times with pestle A and 10-12 times with pestle B on ice. After ensuring that the tissue had been dissociated completely, an additional 500 uL NEB was added into each sample, then transferred from the homogenizer to a 1.7 mL Eppendorf tube with filtration using a 40 µm cell Strainer (Falcon, 352340). Filtered samples were incubated on ice for 5 minutes. Nuclei were centrifuged at 500-600g for 6 minutes at 4°C and the pellet was washed twice with 1 mL ice-cold Wash Buffer (1x PBS, 2% BSA, and 0.2U/uL RNAse Inhibitor). The washed nuclei pellet was resuspended in an appropriate volume of resuspension buffer (1x PBS and 0.04% BSA), filtered through a 40 µm cell strainer (Falcon, 352340). Nuclei were manually counted using a hemacytometer before proceeding to library preparation protocols.

Cell lines

Neuroblastoma (NBL) cell lines, NB1643, CHLA15, CHLA20, COG-N-297 and COG-N-590 were requested from the COG/ALSF Childhood Cancer Repository (www.CCcells.org). THP-1 (Cat # TIB-202) cell line was purchased from American Type Culture Collection (ATCC). All above cell lines were authenticated using STR profiling by the Penn Genomics and Sequencing Core at University of Pennsylvania. Mycoplasma test was done and no contamination was detected.

Whole genome sequencing (WGS)

Human genomic DNA (gDNA) was extracted from isolated nuclei using the Quick-DNA Microprep Plus Kit (Zymo Research, D4074). Libraries were generated from 1-100 ng of gDNA using the Illumina DNA Prep, (M) Tagmentation kit (Illumina, 20018705) according to the manufacturer's instructions. Bead-linked transposomes in tagmentation buffer were added to each sample at 55°C for 15 minutes in a thermal cycler to tagment gDNA and add adapter sequences. After completion of the reaction, tagmentation stop buffer was added to stop the tagmentation reaction. Post-tagmentation cleanup was then performed to remove tagmented and adapter-tagged DNA from beads. This step was followed by the addition of i7 and i5 index adapters to amplify tagmented DNA. The number of PCR cycles was chosen according to directions in the user guide. The i7 and i5 indices were provided in the Nextera DNA CD Indexes kit (Illumina, 20018707). Amplified libraries were then purified using a double-sided bead purification method as outlined in the user guide. The average fragment size of purified libraries was confirmed using the Agilent 2100 Bioanalyzer with the High Sensitivity DNA kit (Agilent Technologies, 5067-4626) and library concentrations were measured using the KAPA library quantification kit (KAPA, KK4835). DNA libraries were pooled and sequenced on an Illumina NovaSeq 6000 using 150x150 bp paired-end reads.

CODEX antibody conjugation

Akoya antibodies were purchased pre-conjugated to their respective CODEX Barcode (**Supplemental Table 11**). All other antibodies were conjugated to their respective CODEX barcode (**Supplemental Table 11**) according to Akoya's PhenoCycler-Fusion user guide using the

antibody conjugation kit (Akoya, 7000009) following the manufacturer's protocol. Briefly, 50 µg of carrier-free antibodies were concentrated by centrifugation in 50kDa MWCO filters (EMD Millipore, UFC505096) and incubated in the antibody disulfide reduction master mix for 30 minutes. Buffer exchange of the antibodies was then performed to stop the reduction reaction by adding conjugation solution followed by centrifugation. Respective CODEX barcodes resuspended in nuclease free water and conjugation solution were added to the concentrated antibody and incubated for 2 hours at room temperature. Conjugated antibodies were purified by 3 buffer exchanges with purification solution. Antibody storage buffer (100 µL) was added to collect the concentrated purified antibodies. Successful conjugation was confirmed using the Agilent 2100 Bioanalyzer with the Agilent Protein High Sensitivity kit (Agilent Technologies, 5067-1575), following the manufacturer's instructions.

Cell culture

Neuroblastoma cells were grown and maintained in Iscove's modified Dulbecco's medium (IMDM) (Thermo Fisher, #12440053) supplemented with 10% fetal bovine serum (FBS) (Cytiva, SH30071.03H), 1% of insulin-transferrin-selenium (ITS) (ThermoFisher, #41400045), and 1% of penicillin-streptomycin (P/S) (ThermoFisher, #15140122) at 37°C and 5% CO_2. THP-1 monocytes were maintained in Roswell Park Memorial Institute (RPMI) 1640 medium (ThermoFisher, #11875085), supplemented with 10% FBS and 1% penicillin-streptomycin at 37°C and 5% CO_2.

Cell viability assay

NB1643 and CHLA15 NBL cells were plated at a density of 10,000 cells/well in 100 µL of complete media in flat-bottom TC-treated 96-well plate (Falcon, 353072). The next day, drug

solutions prepared using complete media were used to determine the IC50 values. DMSO was used as a vehicle control. The media was replaced with 100 µL of treatment media (100 µL/well in 10% FBS and 1% ITS supplemented IMDM media) and incubated for 72 hours. Cell viability was assessed using CellTiter Glo 2.0 (Promega, G9241) reagents as described in the manufacturer's protocol and the GloMax Explorer (Promega) plate reader. Wells containing media but no cells were used to correct for background luminescence signal. Cell viability was normalized to control treated cells. The IC50 for afatinib (MedChemEx, HY-10261) was determined using concentrations ranging from 0-10µM. IC50 value was calculated using the GraphPad Prism 9 dose response-inhibition tool.

Protein extraction

NBL cells were gently washed with ice-cold PBS once and incubated on ice with 1x cell lysis buffer (Cell Sig. #9803) supplemented with 1 mM phenylmethylsulfonyl fluoride (PMSF) (Sigma Aldrich, P7626) and 1% protease and phosphatase inhibitor (PPI) cocktail (Sigma Aldrich, PPC1010) for 5 minutes. NBL cells were lysed with a syringe and frequent vortexing for 5 minutes on ice. After centrifugation at 4°C and maximum speed for 10 minutes, the supernatant was transferred into a new microcentrifuge tube and stored at -80°C for further analysis. THP-1 macrophages were collected with ice-cold DPBS using a cell scraper. After centrifugation, cell pellets from three inserts per group were combined into the same microcentrifuge tube and lysed on ice with the same buffer described above. Protein concentration was determined using the Pierce BCA protein assay (ThermoFisher, #232225) following the manufacturer's protocol.

Immunoblotting

Protein from NBL cell lysates (20-30 μg) and THP-1 macrophages (10-15 μg) were separated on NuPage 4-12% BisTris gel and transferred to polyvinylidene difluroride (PVDF) (ThermoFisher, IB24001) membranes using the Iblot2 system (ThermoFisher). Primary antibodies used in the study include: AKT (Cell Sig., #9272), p-AKT (Cell Sig., #4060), p44/p42 MAPK8 (ERK1/2) (Cell Sig., #9102), p-p44/p42 (p-ERK1/2) (Cell Sig., #4377), HBEGF (Invitrogen, MA535148), α-tubulin (Cell Sig., #2125) and β-actin (Invitrogen, MA1140). Blots were incubated in a blocking solution at room temperature (RT) for 1 hour, consisting of 5% BSA TBS-T (Cell Sig., #9997) for AKT and ERK1/2 phospho-antibodies or 5% non-fat dry milk (NFDM) (LabScientific, 20170808) in TBS-T for other antibodies. Primary antibodies p-AKT, AKT, p-ERK1/2, ERK1/2, and HBEGF (1:1000) and β-actin (1:10000) were diluted in the blocking solution of 5% BSA TBS-T while α-tubulin (1:1000) were prepared in 5% -NFDM TBS-T solution . Primary antibodies were added to the blot and incubated overnight at 4°C. After washing with TBS-T, blots were incubated in secondary antibodies at RT for 1 hour. Secondary antibodies used were anti-rabbit IgG-HRP linked (Cell Sig., #7074) and anti-mouse IgG-HRP-linked (Sigma Aldrich, #A5906), and diluted at a ratio of 1:5000 and 1:10000 in 5% -NFDM TBS-T, respectively. After adding the chemiluminescence substrate (Thermo-Fisher, # PI34577), signal from targeted proteins was detected using the G:BOX Chemi XX6 instrument (Syngene). β-actin and α-tubulin were used as the loading controls. Signal density of the target protein was quantified using ImageJ and normalized to signal density of loading controls. Phospho-protein signals (p-AKT and p-ERK1/2) were normalized to their respective total protein signal (AKT and ERK1/2).

ERBB4 phosphorylation profiling

The Phosphorylation level of ERBB4 in mono-cultured and macrophage co-cultured NBL cells were determined using the PathScan Phospho-HER4/ErbB4(pan-Tyr) Sandwich ELISA kit (Cell Sig., #13125) according to the manufacturer's protocol with minor modifications. Briefly, NBL cells in 6-well plates were harvested 48-hours after monoculture or co-culture and immediately lysed with 140 µL/well of cell lysis buffer supplemented with 1 mM PMSF and 2% PPI. Then, 50 µL of CHLA15 cell lysates were diluted with 50 µL of sample diluent buffer due to high basal level of ERBB4 phosphorylation. Next, 100 µL of undiluted or diluted cell lysates were loaded into the antibody-coated microwells and followed by the rest of protocol. Absorbance at 450 nm was measured by the FLUOstar Omega-BMG LabTech microplate reader. The rest of the cell lysate was used to measure the protein concentration using a BCA protein assay (ThermoFisher, #232225). Absorbance from the PathScan ELISA was normalized to protein concentration. Four biological replicates were used for each cell line and treatment condition.

Co-culture of NBL cell lines with THP-1 macrophages for scRNA-seq profiling

THP-1 cells were plated on 6-well inserts and treated with PMA to induce differentiation to macrophages. Following differentiation, the THP-1 macrophages were co-cultured with CHLA15 or CHLA20 neuroblastoma cells for 48 hours in serum-free co-culture media. HB-EGF activity was inhibited by adding 4 µg/mL CRM197 to the co-culture media. ERBB activity was inhibited by adding afatinib at specific concentrations based on the 72-hour IC50 values to the co-culture media: 1µM for CHLA15 (IC_{50} = 1.134µm) and 2µM for CHLA20 (IC_{50} = 4µM). After 48 hours of mono- or co-culture with or without inhibitors, macrophages and neuroblastoma cells were collected separately via accutase (Sigma-Aldrich, #A6964) treatment, and live cells were enriched with Dead Cell Removal Kit (Miltenyi Biotec, #130-090-101). After confirming that the cell

viability was greater than 70% by the trypan-blue exclusion assay, samples were grouped into the following experimental conditions: 1) mono-cultured THP-1 macrophages, 2) mono-cultured NBL cells, 3) co-cultured THP-1 macrophages and NBL cells, 4) CRM197-treated macrophages and NBL cells, 5) afatinib treated co-cultured macrophages and NBL cells.

Single-cell RNA-seq of mono- and co-cultured neuroblastoma cells and THP-1 macrophages

Cells were stained with Human TruStain FcX and TotalSeq-B anti-human hashtag antibodies (BioLegend) as per the manufacturer's instructions. Pooled single cell suspensions underwent library preparation following the 10x Genomics protocol using Chromium X with the Chromium Single Cell 3′ Reagent Kit V3.1 (dual index) and Chromium Single Cell 3′ Feature Barcode Library Kit as per manufacturer's instructions. Fragment size was determined using the Bioanalyzer Agilent 2100 with the High Sensitivity DNA chip (Agilent Technologies, 5067-4626). Indexed libraries were pooled and sequenced on an Illumina NovaSeq 6000 using sequencing parameters 28:10:10:90 (read1:i5:i7:read2, bp) with an average sequencing depth of 50,000 read pairs per cell.

TH-MYCN mouse model

Animal studies followed protocols approved by the Children's Hospital of Philadelphia Institutional Animal Care and Use Committee. 129x1/SvJ mice transgenic for the TH-MYCN construct were originally obtained from Bill Weiss (University of California, San Francisco). TH-MYCN hemizygous mice[1] were bred, and offspring were genotyped from tail-snip-isolated DNA using qPCR. In this model, MYCN expression is targeted to the murine neural crest under the tyrosine hydroxylase promoter, recapitulating the hallmark features of human neuroblastoma. Tumors are fully penetrant and arise at autochthonous sites in an immunocompetent host with

lethality by day 60 of life. Only TH-MYCN$^{+/+}$ mice were used in this study. Mice were monitored for tumors by palpation by a single experienced animal technician and randomized to a treatment arm when tumors were small to medium in size (~0.8 grams tumor by necropsy in n=3 control mice): vehicle (PBS) or 20 mg/kg dose of cyclophosphamide by intraperitoneal injection (IP) three times (Monday/Wednesday/Friday) for 2 weeks. The six mice we used were aged 44,51,52, 52, 52, and 55 days, respectively. Mice were weighed and assessed for tumor growth and symptoms at least three times weekly. Mice were euthanized for pre-defined humane endpoints related to overall health or tumor burden (hunching, immobility, hindlimb paresis, weight loss, respiratory distress). The tumor was obtained at necropsy and bisected, with a portion flash-frozen and a separate portion formalin-fixed and paraffin-embedded for subsequent studies.

Xenium spatial transcriptomics

Formalin fixed and paraffin embedded (FFPE) mouse tissue blocks were sectioned into 5 μm sections within the 10.45 mm x 22.45 mm capture area on Xenium slides. Slides were processed using Xenium Prime Sample Preparation Reagents (10x Genomics, PN-1000720) as per the manufacturer's instructions. After baking the slides at 60°C for 30 minutes in a thermal cycler, slides were deparaffinized twice in xylene for 10 minutes. Slides were then rehydrated in a series of different ethanol concentrations: twice in 100% ethanol for 3 minutes, twice in 96% ethanol for 3 minutes, once in 70% ethanol for 3 minutes, and 20 seconds in nuclease free water. Decrosslinking buffer was added to slides assembled in the Xenium cassette v2 and incubated in a thermal cycler at 80°C for 30 minutes followed by three PBS-T (1X PBS and 0.05% Tween-20) washes. Xenium 5K Mouse PTP Priming Oligos (10x Genomics, PN- 2001226) were thawed, briefly centrifuged, preheated to 95°C for 2 minutes, and cooled on ice for 1 minute. Priming

hybridization mix was prepared as per the protocol, and slides were incubated with the mix at 50°C for 1.5 hours followed by post priming hybridization wash at 50°C for 30 minutes in a thermal cycler. Slides were then treated with RNase mix at 37°C for 20 minutes followed by polishing reaction mix at 37°C for 1 hour. Xenium 5K Mouse PTP Panel Probes (10x Genomics, 2001227) were thawed, briefly centrifuged, preheated to 95°C for 2 minutes, and cooled on ice for 1 minute. The probes were hybridized to targeted mRNA at 50°C overnight followed by a post-hybridization wash at 35°C for 15 minutes. Hybridized probes were ligated at 42°C for 30 minutes and amplification enhancement was performed at 4°C for 2 hours in a thermal cycler followed by a post-amplification enhancement wash. After the post-amplification enhancement wash, ligated probes were amplified at 30°C for 1.5 hours followed by three washes in TE buffer. Slides were then washed in a series of ethanol concentrations: once in 70% ethanol, twice in 100% ethanol, and once in 70% ethanol for 2 minutes in each. Tissue slides were blocked for 1 hour at room temperature in Xenium Block and Stain buffer followed by staining with cell segmentation antibodies overnight at 4°C using Xenium Cell Segmentation Staining Reagents (10x Genomics, PN-1000661). Antibody staining was followed by stain enhancement at room temperature for 20 minutes and two PBS-T washes. Slides were then incubated in diluted reducing agent B for 10 minutes and dehydrated in 70% ethanol for 1 minute and then 2X in 100% ethanol for 1 minute. Autofluorescence quenching was done in the dark for 10 minutes at room temperature as per the protocol. After washing the slide three times in 100% ethanol for 2 minutes, slides were dried at 37°C for 5 minutes in a thermal cycler in the dark. Tissue slides were rehydrated in 1X PBS for 1 minute followed by incubation in PBS-T for 2 minutes at room temperature in dark. Xenium Nuclei Staining Buffer was used to stain nuclei and samples were washed three times in PBS-T in the dark. Tissue slides can be stored for a week in PBS-T at 4°C in the dark. The readiness test was

initiated on the Xenium Analyzer instrument prior to loading consumables and tissue slides. Xenium Decoding Consumables v2, PN-1000726; Xenium Reagent Bottles, PN-1000730; and Xenium Prime 5K Decoding Reagents, PN-1000740 from 10x Genomics were then prepared and loaded on the Xenium analyzer instrument as per the manufacturer's instructions. The bottom of the tissue slide in Xenium cassette was cleaned with 70% isopropanol and slides in the cassette without the lid were loaded on the instrument. Imaging, decoding, segmentation, and cell assignment was performed by Xenium Analyzer version 3.1.

Whole genome sequencing (WGS) data processing

For each sample, one region was selected and deeply sequenced by whole genome sequencing. The raw reads were mapped to the Genome Reference Consortium Human Build 38 patch release 13 (GRCh38.p13) using the Burrows-Wheeler Aligner (BWA)[2]. The aligned BAM files were sorted and indexed using samtools[3]. Genomic coverage rates were computed using the samtools *depth* function.

H&E image registration

40x H&Es were registered to the 20x CODEX imaging space using wsireg (https://github.com/NHPatterson/wsireg) with affine and rigid transformations using the default parameters. The DAPI and NaKATPase channels in CODEX image were used as input to label the nucleus and membrane, respectively matching hematoxylin and eosin.

Identification of malignant cells in snRNA-seq using a neural network classifier

To annotate high-confidence malignant cells as training data for an artificial neural network (ANN)-based classifier, we first applied Clonalscope[4], a non-parametric clustering method for identifying cancer clones using single-cell RNA-seq data and matched bulk whole-genome sequencing data. By incorporating copy number variation (CNV) information from paired WGS data as a prior, ClonalScope increases the accuracy of malignant cell detection. Using this approach, we identified 16 samples including ~83,000 cells in which the snRNA-seq-based CNV profiles were highly concordant with the CNV profiles identified by the WGS data (**Extended Data Fig. 2a-b**). In these 16 datasets, *de novo* clustering was able to accurately separate malignant and non-malignant cells based on the mean CNV profiles between the snRNA-seq and WGS data. Therefore, we manually identified the high-confidence malignant cells for each of these 16 datasets in order to train an artificial neural network (ANN) based classifier for distinguishing neoplastic and non-neoplastic cells across the entire dataset.

We used a fully connected feedforward neural network with one hidden layer. Cross-entropy was used as the loss function for the binary classification for N cells:

$$L = \sum_{i=1}^{N} [y_i * \log(\hat{y}_i) + (1 - y_i) * \log(1 - \hat{y}_i)]$$

where y_i is the true value $\in \{0,1\}$ and $\hat{y}_i$is the softmax probability for $y_i = 0$ or 1. We tested the different numbers of hidden nodes (64, 128, 256, 512, 1024, 2048) using 6-fold cross validation. Briefly, we divided the ~83,000 high confidence malignant cells in the 16 datasets into 6 groups. Each group was held out, and the classifier with a given number of hidden nodes was trained on the other 5 groups to predict the malignancy status on the held-out group. For each parameter choice, this procedure was performed 6 times and the average loss of the 6 repetitions was computed. We used all predicted malignant cells to compute AUCs and ROC curves for each

configuration. Based on the AUC metric, we chose 1024 hidden nodes in the classifier and retrained the final model with all cells in the 16 datasets (**Extended Data Fig. 2d**).

InferCNV analysis

To further corroborate malignant cells predicted by our ANN-based method, we conducted inferCNV[5] analysis to evaluate if known copy number variations (CNVs) in NBL could be detected in putative malignant cells predicted by our ANN-based method. The normalized gene-by-cell expression matrix for each patient was used as the input with the following parameters settings: *cutoff = 0.1* and *min_cells_per_gene = 5*. Immune cells predicted to be non-malignant were utilized as normal reference cells.

Projection of neoplastic cells onto fetal adrenal medulla scRNA-seq reference

To characterize the developmental trajectory of neuroblastoma tumor cells, we performed reference mapping of our snRNA-seq data onto recently published scRNA-seq data (Jansky *et al.*)[6] of fetal adrenal gland collected from healthy human embryos. First, the adrenal medullary cells in this dataset were subset since they constitute the tissue of origin for NBL tumor cells, and a UMAP reduction was recomputed using the top 20 principal components in Seurat. Then, we projected cells from our snRNA-seq data onto the embryonic data using the *FindTransferAnchors* and *MapQuery* functions in Seurat v4 with the principal component analysis as the reference reduction with the top 50 dimensions. This process automatically assigned each cell in the NBL dataset to the closest matching healthy embryonic counterpart.

Differential expression and pathway enrichment analysis of malignant cells

Calculation of differentially expressed genes across clusters, neoplastic cell states and timepoints was performed using the *FindAllMarkers* function in Seurat with parameters "*log2fc.threshold* = 0.25 *test.use* = *'LR', latent.vars* = *c*(*'nCount_RNA', 'percent.mt'*), *max.cells.per.ident* = 1000." Pathway enrichment analysis was conducted using significant differentially expressed genes (p value < 0.001 and pct1 - pct2 > 0.05) as input to enrichR[7]. The following four pathway collections were used: KEGG[8], Gene Ontology[9] Biological Processes, Molecular Signatures Database cancer hallmark gene sets[10] and REACTOME[11].

Differential peak accessibility analysis

To obtain the differentially accessible peaks (DAPs) across neoplastic cell states, we utilized edgeR[12] on pseudobulk state-based chromatin accessibility profiles. Briefly, we first constructed a pseudobulk peak-by-state matrix for each sample by aggregating all counts associated with the peak for cells in the given neoplastic state. Peaks that were accessible in less than 1% cells of every sample were filtered out from downstream analysis. Subsequently, we applied edgeR to the pseudobulk data, comparing each neoplastic state with the rest of neoplastic states. Differentially accessible peaks were defined based on $p<0.05$ and $logFC>0.5$. These peaks were used for downstream analysis.

Differential transcription factor motif activity analysis

Transcription factors (TFs) with differential motif activity across neoplastic cell states were identified as follows. We first applied chromVAR[13] to identify differentially accessible motifs across the putative malignance cells in the snATAC-seq data. This was implemented through the Signac functions *AddMotifs* and *RunChomVAR*, with the motif database *human_pwms_v2* in the

R chormVARmotifs package. Then, we calculated differentially accessible motifs using the *FindAllMarkers* function with parameters "*max.cells.per.ident*=500*, fc.name=avg_diff*" on the output of the chromVAR analysis. The TFs with differential motif activity were defined based on an output of avg_dff > 0.5, pct1 > 0.1 and p < 0.01.

Calculation of single-cell-based signature scores

Single-cell signature scores for neoplastic and macrophage phenotypes were computed using the *AddModuleScore* function in Seurat with manually curated signature genes (**Supplementary Table 10**). The signatures of cell cycle S and G2M phases were calculated using the *CellCycleScoring* function in Seurat. Bulk RNA-seq signature scores based on neoplastic cell states were calculated as the weighted average of the expression of upregulated genes (**Supplementary Table 5**) for that state. The weight of the gene was based on the results of the *FindAllMarkers* function in Seurat and defined as the expression magnitude fold change multiplied by the difference in the percent of cells expressing the gene between the state of interest versus all other states, i.e. (avg_log2FC * (pct1 -pct2).

Deconvolution of bulk gene expression datasets

We used CIBERSORTx[14] to deconvolute the bulk gene expression data from the SEQC and Cangelosi et al. datasets. Neoplastic cell states, macrophage subsets, and other normal cell types from our snRNA-Seq data were used as the reference. Each cell population in the reference data was downsampled to 10,000 cells if more than 10,000 cells were available, followed by

renormalized based on relative counts. CIBERSORTx was then run with "S-mode" batch correction.

scRNA-seq sample demultiplexing and data analysis of *in vitro* co-cultured cells

Raw reads were aligned to the Genome Reference Consortium Human Build 38 patch release 13 (GRCh38.p13) assembly and preprocessed using CellRanger v7.1.0. Pooled sequencing data were then demultiplexed using the R package deMULTIplex2[15] with default parameters, followed by the removal of doublets using the R package DoubletFinder with an expected doublet rate 0.04. High-quality cells were retained based on the following criteria: UMI count >2,000, expressing 500-10,000 genes, and <5% of UMIs mapped to mitochondrial genes. The filtered dataset was normalized with log-normalization, and 2,000 highly variable genes were selected for a principal component analysis (PCA). UMAP embeddings were computed using the first 20 PCA dimensions for visualization. To annotate malignant cell states, we projected *PHOX2B*+ cell clusters onto our annotated patient snRNA-seq data using the *FindTransferAnchors* and *MapQuery* functions in Seurat. Subsequently, differential gene expression analysis was conducted using the *FindMarkers* function in Seurat with logistic regression ('LR') as the testing method and the projected cell state as a covariate.

Xenium spatial transcriptomics data analysis

Preprocessed Xenium data from the Xenium Onboard Analysis software (Xenium Analyzer) was further processed using the Squidpy[16] software in the Python Scanpy[17] framework. Cells with fewer than 100 transcripts and genes expressed in fewer than 50 cells were filtered out. The remaining cells were log-normalized, and a PCA was conducted using all available genes. A

UMAP projection was subsequently constructed for visualization using default parameters. To remove batch effect, we applied Harmony[18] integration, using the mouse identifier as the batch variable. Cells were clustered with the Louvain algorithm via the *scanpy.tl.louvain* function with resolution = 0.8. Cells in cluster 2 were removed due to a very low average number of transcripts per cell compared to other clusters. A second round of PCA analysis, UMAP construction, Harmony integration and clustering were performed on the remaining cells. The resulting cell clusters were then manually annotated based on marker gene expression.

Neuroblasts and macrophages were further annotated using our neoplastic and macrophage snRNA-seq atlas as references. This was achieved through the Seurat label transfer pipeline. Specifically, we reanalyzed the annotated neoplastic and macrophage snRNA-seq data in a manner similar to previous analyses but restricting the analysis to 4823 homologues human genes present in the Xenium mouse gene panel. Xenium cells for each mouse sample were then integrated with the reference data using the *FindTransferAnchors* function with '*reduction = rpca.*' The cell state of each Xenium cell was subsequently predicted using the *TransferData* function in Seurat with default parameters.

A ligand-receptor (LR) interaction analysis was performed as previously described[19–21]. Briefly, for each sample, we identified all proximal pairs of neuroblast and macrophage subsets, defined as cell centroids within 50 μm of each other, using the *squidpy.gr.spatial_neighbors* function in Squidpy. For each LR pair, we calculated the product of the log-normalized expression of the ligand in the ligand source cells and the receptor in the receptor source cells, defining the LR score as the sum of these products across all proximal cell pairs. The ligand-receptor pairs tested were output from our cell-cell-interaction analysis of human snRNA-seq data. The

enrichment significance was determined using empirical shuffling: we randomly selected the same number of non-proximal cell pairs that were separated by greater than 50 μm and calculated the LR score. This process was repeated 100 times to generate a null distribution of LR scores. A p-value was then determined using a one-tailed Z-test. The fold change was calculated by comparing the LR score of the proximal pairs to the average LR score of the non-proximal permutations. For each LR pair, the minimum p-value and maximum fold change across samples were reported.

References

1. Weiss, W. A., Aldape, K., Mohapatra, G., Feuerstein, B. G. & Bishop, J. M. Targeted expression of MYCN causes neuroblastoma in transgenic mice. *EMBO J.* **16**, 2985–2995 (1997).
2. Li, H. & Durbin, R. Fast and accurate short read alignment with Burrows–Wheeler transform. *Bioinformatics* **25**, 1754–1760 (2009).
3. Li, H. *et al.* The Sequence Alignment/Map format and SAMtools. *Bioinformatics* **25**, 2078–2079 (2009).
4. Wu, C.-Y. *et al.* Cancer subclone detection based on DNA copy number in single cell and spatial omic sequencing data. *bioRxiv* (2022) doi:10.1101/2022.07.05.498882.
5. Kenny, P. A. InferCNV, a python web app for copy number inference from discrete gene-level amplification signals noted in clinical tumor profiling reports. *F1000Res.* **8**, 807 (2019).
6. Jansky, S. *et al.* Single-cell transcriptomic analyses provide insights into the developmental origins of neuroblastoma. *Nat. Genet.* **53**, 683–693 (2021).
7. Kuleshov, M. V. *et al.* Enrichr: a comprehensive gene set enrichment analysis web server 2016 update. *Nucleic Acids Res.* **44**, W90-7 (2016).
8. Kanehisa, M., Furumichi, M., Sato, Y., Kawashima, M. & Ishiguro-Watanabe, M. KEGG for taxonomy-based analysis of pathways and genomes. *Nucleic Acids Res.* **51**, D587–D592 (2023).
9. Ashburner, M. *et al.* Gene ontology: tool for the unification of biology. The Gene Ontology Consortium. *Nat. Genet.* **25**, 25–29 (2000).
10. Liberzon, A. *et al.* The Molecular Signatures Database (MSigDB) hallmark gene set collection. *Cell Syst.* **1**, 417–425 (2015).

11. Jassal, B. *et al.* The reactome pathway knowledgebase. *Nucleic Acids Res.* **48**, D498–D503 (2020).

12. Robinson, M. D., McCarthy, D. J. & Smyth, G. K. edgeR: a Bioconductor package for differential expression analysis of digital gene expression data. *Bioinformatics* **26**, 139–140 (2010).

13. Schep, A. N., Wu, B., Buenrostro, J. D. & Greenleaf, W. J. chromVAR: inferring transcription-factor-associated accessibility from single-cell epigenomic data. *Nat. Methods* **14**, 975–978 (2017).

14. Newman, A. M. *et al.* Determining cell type abundance and expression from bulk tissues with digital cytometry. *Nat. Biotechnol.* **37**, 773–782 (2019).

15. Zhu, Q., Conrad, D. N. & Gartner, Z. J. deMULTIplex2: robust sample demultiplexing for scRNA-seq. *Genome Biol.* **25**, 37 (2024).

16. Palla, G. *et al.* Squidpy: a scalable framework for spatial omics analysis. *Nat. Methods* **19**, 171–178 (2022).

17. Wolf, F. A., Angerer, P. & Theis, F. J. SCANPY: large-scale single-cell gene expression data analysis. *Genome Biol.* **19**, (2018).

18. Korsunsky, I. *et al.* Fast, sensitive and accurate integration of single-cell data with Harmony. *Nat. Methods* **16**, 1289–1296 (2019).

19. Zhang, M. *et al.* Molecularly defined and spatially resolved cell atlas of the whole mouse brain. *Nature* **624**, 343–354 (2023).

20. Fang, R. *et al.* Conservation and divergence of cortical cell organization in human and mouse revealed by MERFISH. *Science* **377**, 56–62 (2022).

21. Sussman, J. H. *et al.* A longitudinal single-cell and spatial multiomic atlas of pediatric high-grade glioma. *bioRxivorg* (2024) doi:10.1101/2024.03.06.583588.
