# Supplementary material for: Longitudinal single-cell multiomic atlas of high-risk neuroblastoma reveals chemotherapy-induced tumor microenvironment rewiring
Source: Nat Genet. 2025 Apr 14;57(5):1142–54. doi: 10.1038/s41588-025-02158-6 (PMC12081299; doi:10.1038/s41588-025-02158-6)
Supplement: Supplementary file 2 — Reporting Summary [file 41588_2025_2158_MOESM2_ESM.pdf]

nature portfolio

Corresponding author(s): Kai Tan

Last updated by author(s): 02/03/2025

Reporting Summary

Nature Portfolio wishes to improve the reproducibility of the work that we publish. This form provides structure for consistency and transparency in reporting. For further information on Nature Portfolio policies, see our Editorial Policies and the Editorial Policy Checklist.

Statistics

For all statistical analyses, confirm that the following items are present in the figure legend, table legend, main text, or Methods section.

n/a	Confirmed	
☐	☒	The exact sample size (*n*) for each experimental group/condition, given as a discrete number and unit of measurement
☐	☒	A statement on whether measurements were taken from distinct samples or whether the same sample was measured repeatedly
☐	☒	The statistical test(s) used AND whether they are one- or two-sided<br>*Only common tests should be described solely by name; describe more complex techniques in the Methods section.*
☐	☒	A description of all covariates tested
☐	☒	A description of any assumptions or corrections, such as tests of normality and adjustment for multiple comparisons
☐	☒	A full description of the statistical parameters including central tendency (e.g. means) or other basic estimates (e.g. regression coefficient) AND variation (e.g. standard deviation) or associated estimates of uncertainty (e.g. confidence intervals)
☐	☒	For null hypothesis testing, the test statistic (e.g. *F*, *t*, *r*) with confidence intervals, effect sizes, degrees of freedom and *P* value noted<br>*Give P values as exact values whenever suitable.*
☒	☐	For Bayesian analysis, information on the choice of priors and Markov chain Monte Carlo settings
☐	☒	For hierarchical and complex designs, identification of the appropriate level for tests and full reporting of outcomes
☐	☒	Estimates of effect sizes (e.g. Cohen's *d*, Pearson's *r*), indicating how they were calculated

Our web collection on statistics for biologists contains articles on many of the points above.

Software and code

Policy information about availability of computer code

Data collection

No software was used for data collection

Data analysis

Publicly available tools were used for data analysis, statistics and visualization, with specific tools and versions described in the methods section, and listed below.

Software:
R (v. 4.2.3)
Python (v.3.10.4)
GraphPad Prism 9

R packages:
Seurat (v4.0.5)
harmony (v0.1.1)
survival (v3.5.5)
ggsurvfit (v0.3.1)
edgeR (v.3.40.2)
InferCNV v1.6.0
chromVAR (v1.12.0)
Signac (v1.12.0)
enrichR (v3.2)

Sequence Analysis
cellranger v3.1.0 --patient cohort
cellranger v7.1.0-- in vitro experiment
cellranger-atac v1.1.0
scATAC-pro v1.5.1
chromVARmotifs (v0.2.0)
Clonalscope (v1.0.1)

CODEX and Xenium data analysis
Akoya's-Fusion (v1.0.8)
Mesmer(v0.12.9)
QuPath (v0.4)
Napari (v0.4.18)
Scanpy (v1.10.3)
Xenium Analyzer (v 3.1)
Squipdy (v 1.6.1)

Custom Codes
https://github.com/tanlabcode/NBL_scMultiomics_Paper

For manuscripts utilizing custom algorithms or software that are central to the research but not yet described in published literature, software must be made available to editors and reviewers. We strongly encourage code deposition in a community repository (e.g. GitHub). See the Nature Portfolio guidelines for submitting code & software for further information.

Data

Policy information about availability of data

All manuscripts must include a data availability statement. This statement should provide the following information, where applicable:

- Accession codes, unique identifiers, or web links for publicly available datasets
- A description of any restrictions on data availability
- For clinical datasets or third party data, please ensure that the statement adheres to our policy

Data from this study have been deposited at the Human Tumor Atlas Network (HTAN) data portal: https://humantumoratlas.org/publications/hta4_2025_nature-genetics_wenbao-yu. For the snRNA-seq, snATAC-seq and WGS data this includes sequencing reads and processed data including read alignments, gene-by-cell or peak-by-cell matrices, and variant call files. We also deposited the processed snRNA-Seq data to the CELLxGENE database at https://cellxgene.cziscience.com/collections/cee845e3-ec04-4781-9e2a-28734bb4f7ba for easy interactive exploration. For the CODEX data, this includes multi-channel images, segmentation masks, and marker-by-cell matrix. For all data types, Seurat objects with annotations and dimensional reductions are provided for each data type. The linkage between HTAN patient IDs and sample IDs is provided in Supplementary Table 2. The processed Xenium transcriptomic and scRNA-seq data in the mono- and co-culture experiments have been deposited to Zenodo repository: https://doi.org/10.5281/zenodo.14261274.

Research involving human participants, their data, or biological material

Policy information about studies with human participants or human data. See also policy information about sex, gender (identity/presentation), and sexual orientation and race, ethnicity and racism.

Reporting on sex and gender	All patients in the cohort were male or female according to biological sex when available, and no information on gender identity was collected.
Reporting on race, ethnicity, or other socially relevant groupings	Race or ethnicity is reported when available, but is not relevant to the methods or findings of the present study. In our computational models, each patient is considered as variable feature which accounts for confounding factors that may be specific to a particular patient. However, the cohort size is not large enough in order to conduct subgroup analyses based on race.
Population characteristics	22 patients with pediatric high-risk neuroblastoma were enrolled in the study. The cohort's age ranged from 6 months to 13 years and had a male-to-female ratio of 0.57. The tumor specimens were resected from multiple anatomic locations, detailed in Supplementary Table 1.
Recruitment	Primary samples were obtained from patients with high-risk neuroblastoma banked at the Children's Hospital of Philadelphia (CHOP) Childhood Cancer Research (CCCR) Registry. The patient selection was built based on specimen availability.
Ethics oversight	Biorepositories were obtained with parent informed consent according to the Declaration of Helsinki and Institutional Review Board approval from the Children's Hospital of Philadelphia

Note that full information on the approval of the study protocol must also be provided in the manuscript.

Field-specific reporting

Please select the one below that is the best fit for your research. If you are not sure, read the appropriate sections before making your selection.

☒ Life sciences ☐ Behavioural & social sciences ☐ Ecological, evolutionary & environmental sciences

For a reference copy of the document with all sections, see nature.com/documents/nr-reporting-summary-flat.pdf

Life sciences study design

All studies must disclose on these points even when the disclosure is negative.

Sample size	No sample size calculation was performed. The number of patient samples was primarily determined by the availability of tumor samples in the biobank at the Children's Hospital of Philadelphia (CHOP) Childhood Cancer Research (CCCR) Registry. Sample size was limited majorly due to the rarity of neuroblastoma.
Data exclusions	All sample with reasonable sample quality as assessed histologically and that passed standard QC filters after processing were included in the analysis. Sample QC for scRNA-seq and scATAC-seq is summarized in Extended Data Figure 1.
Replication	Two regions (replicates) per sample were used for each patient to perform snRNA-seq data. No replication was performed for WGS, snATAC-seq and CODEX experiments due to lack of materials. The in vitro experiments were successfully done on 2-5 replicates (the detailed n for each experiment was given in the legend of figure6). The in vivo experiment was successfully performed on 3 mice per condition.
Randomization	Randomization was not applicable because this is retrospective study for clinical and genomic analyses. Therefore, patients were already determined to have particular disease subtypes and/or treatments and could not be randomized into groups solely for the purpose of this study.
Blinding	Blinding was not applicable because this is retrospective study for clinical and genomic analyses. Therefore, patient metadata was already available and known by researchers a priori in order to select appropriate samples for our cohort design. All sequencing and sample preparations were blinded and randomized.

Reporting for specific materials, systems and methods

We require information from authors about some types of materials, experimental systems and methods used in many studies. Here, indicate whether each material, system or method listed is relevant to your study. If you are not sure if a list item applies to your research, read the appropriate section before selecting a response.

Materials & experimental systems

n/a	Involved in the study	
☐	☒	Antibodies
☐	☒	Eukaryotic cell lines
☒	☐	Palaeontology and archaeology
☐	☒	Animals and other organisms
☐	☒	Clinical data
☒	☐	Dual use research of concern
☒	☐	Plants

Methods

n/a	Involved in the study	
☒	☐	ChIP-seq
☒	☐	Flow cytometry
☒	☐	MRI-based neuroimaging

Antibodies

Antibodies used

Antigen,Clone(s),Vendor,Catalogue Number,Dilution
Na/K-ATPase,EP1845Y,Abcam,ab167390,1:50
ERBB4,182803,R&D Systems,MAB1131,1:50
ALK,POLYCLONAL,Thermo Fisher Scientific,51-3900,1:50
NCAM-1/ CD56,POLYCLONAL,Novus Biologicals,AF2408,1:50
CD8,C8/144B,Akoya Biosciences,ab256584,1:200
aSMA,POLYCLONAL,Abcam,ab5694,1:50
HBEGF,POLYCLONAL,R&D Systems,AF-259-NA,1:29
MRP4,EPR20403,Abcam,ab235624,1:63
NLRP3,768319,Novus Biologicals,MAB7578,1:50
CD206,5C11,Novus Biologicals,H00004360-M02,1:50
Ki67,B56,Akoya Biosciences,4250019,1:200
GYPC,POLYCLONAL,Thermo Fisher Scientific,PA5-80680,1:50
TrKB,POLYCLONAL,Novus Biologicals,AF1494,1:50
TGFA,EPR15346,Abcam,ab224266,1:50
CD4,EPR6855,Akoya Biosciences,4550112,1:200
CD68,KP1,Akoya Biosciences,4550113,1:200
SERCA2,EPR9392,Abcam,ab238426,1:50
ISL1,EPR10362,Abcam,ab238919,1:50
CD45,D9M81,Akoya Biosciences,4550121,1:200
CD11c,EP1347Y,Abcam,ab216655,1:50
GD2,14G2a,Biolegend,357302,1:83
L1CAM,L1-OV198.5,Biolegend,371602,1:50
C1QC,EPR2984Y,Abcam,ab247391,1:50

CD105,EPR19911-220,Abcam,ab252345,1:50
CLSTN2,POLYCLONAL,Novus Biologicals,AF5480,1:50
CD3E,EP449E,Akoya Biosciences,4550119,1:200
PPP2R2C,6D1,Novus Biologicals,H00005522-M01,1:50
PHOX2B,EPR14423,Abcam,ab216456,1:50
BMP7,164311,R&D Systems,MAB3541,1:50
CD31,EP3095,Akoya Biosciences,4250009,1:200
MYCN,D4B2Y,Cell Signaling Technology,69006SF,1:50
CD20,L26,Akoya Biosciences,4450018,1:200
CD163,EDHu-1,Novus Biologicals,NB110-40686,1:50
Vimentin,RV202,Novus Biologicals,NBP1-97672,1:50
Nestin,196908,Novus Biologicals,MAB1259,1:50
IL1RAPL1,MM0353-3R16,Novus Biologicals,NBP2-11648,1:50
CD2,EPR6451,Abcam,ab131276,1:50
SV2C,3D8,Novus Biologicals,H00022987-M01,1:50

Akt, Not Applicable, Cell Signaling Tech (CST), 9272, 1:1000
Phospho-Akt (Ser473) (p-AKT), (D9E) XP, Cell Signaling Tech (CST), 4060, 1:1000
p44/p42 MAPK (Erk1/2), Not Applicable, Cell Signaling Tech., 9102, 1:1000
Phospho-p44/42 MAPK (Erk1/2) (Thr202/Tyr204), 197G2, Cell Signaling Tech.,4377, 1:1000
α-Tubulin, 11H10, Cell Signaling Tech., 2125, 1:1000
HBEGF, ARC0663, Invitrogen, MA5-35148, 1:1000
Beta-Actin, 15G5A11/E2, Invitrogen, MA1140, 1:10000
Anti-rabbit IgG-HRP linked, Not Applicable, Cell Signaling Tech., 7074, 1:5000
Anti-mouse IgG-HRP-linked, Not Applicable, Millipore Sigma, A5906, 1:10000

Validation

All conjugated primary antibodies commercially available from Akoya Biosciences have been extensively validated for different Human FFPE tissue samples on Phenocycler fusion (CODEX) by the manufacturer. Antibody dilution was used as recommended by Akoya Biosciences. Further information on the validation and titration of these antibodies can be found or requested on the manufacturer's website.
Antibodies acquired from other manufacturers were conjugated in house and titrated on Phenocycler fusion to determine the appropriate antibody dilutions for Human Neuroblastoma FFPE tissue samples. Validation using immunohistochemistry (IHC) for these antibodies has been described on their respective manufacturer's website as below:
Anti-human Sodium Potassium ATPase antibody, ab167390 (https://www.abcam.com/en-us/products/primary-antibodies/sodium-potassium-atpase-antibody-ep1845y-bsa-and-azide-free-ab167390).
Potassium ATPase antibody has also been tested for CODEX in the below paper,
https://www.sciencedirect.com/science/article/pii/S0092867420308709#app2.
Anti-human ErbB4/Her4 antibody, MAB1131 (https://www.rndsystems.com/products/human-erbb4-her4-antibody-182803_mab1131).
Anti-human ALK Polyclonal antibody, 51-3900 (https://www.thermofisher.com/antibody/product/ALK-Antibody-Polyclonal/51-3900).
Anti-human NCAM-1/CD56 antibody, AF2408 (https://www.novusbio.com/products/ncam-1-cd56-antibody_af2408).
Anti-human alpha smooth muscle Actin antibody, ab5694 (https://www.abcam.com/en-us/products/primary-antibodies/alpha-smooth-muscle-actin-antibody-ab5694). Anti-human alpha smooth muscle Actin antibody has also been tested for CODEX in the below paper,
https://www.sciencedirect.com/science/article/pii/S0092867420308709#app2.
Anti-human HB-EGF antibody, AF-259-NA (https://www.rndsystems.com/products/human-hb-egf-antibody_af-259-na).
Anti-human MRP4 antibody, ab235624 (https://www.abcam.com/en-us/products/primary-antibodies/mrp4-antibody-epr20403-bsa-and-azide-free-ab235624).
Anti-human NLRP3/NALP3 antibody, MAB7578 (https://www.novusbio.com/products/nlrp3-nalp3-antibody-768319_mab7578#PublicationSection).
Anti-human MMR/CD206/Mannose Receptor antibody, H00004360-M02 (https://www.novusbio.com/products/mmr-cd206-mannose-receptor-antibody-5c11_h00004360-m02).
Anti-human GYPC Polyclonal antibody, PA5-80680 (https://www.thermofisher.com/antibody/product/GYPC-Antibody-Polyclonal/PA5-80680).
Anti-human TrkB antibody, AF1494 (https://www.rndsystems.com/products/human-mouse-rat-trkb-antibody_af1494).
Anti-human TGF alpha antibody, ab224266 (https://www.abcam.com/en-us/products/primary-antibodies/tgf-alpha-antibody-epr15346-bsa-and-azide-free-ab224266?srsltid=AfmBOorGXiBeh0prkP-rJgdyA4n82zilVZ2hjvDewlfUrmch2l50aayr).
Anti- human SERCA2 ATPase antibody, ab238426 (https://www.abcam.com/en-us/products/primary-antibodies/serca2-atpase-antibody-epr9392-bsa-and-azide-free-ab238426?srsltid=AfmBOorA9v7g9pYj_MQatadg2TPo7hxeUTlO7zOj9z-dQq-twIRwfOLT).
Anti-Islet 1 (ISL1) antibody, ab238919 (https://www.abcam.com/en-us/products/primary-antibodies/islet-1-antibody-epr10362-bsa-and-azide-free-ab238919?srsltid=AfmBOoru2DH_2ZKu-V3fBiT6qo3v1aab5MHeGukwoM9DdxFbu4sLBhLI).
Anti-human CD11c antibody, ab216655 (https://www.abcam.com/en-us/products/primary-antibodies/cd11c-antibody-ep1347y-bsa-and-azide-free-ab216655?srsltid=AfmBOopbRbqbaHOfqGhlOftT37ypW5SWuVg2nsnN3-b2qzy0jI3Upy0I). Anti-human alpha smooth muscle Actin antibody, ab5694 (https://www.abcam.com/en-us/products/primary-antibodies/alpha-smooth-muscle-actin-antibody-ab5694). Anti-human CD11c antibody has also been tested for CODEX in the below paper,
https://www.sciencedirect.com/science/article/pii/S0092867420308709#app2.
Anti-human Ganglioside GD2 antibody, 357302 has been tested on Phenocyler fusion in house and has been shown to co-label with PHOX2B.
Anti-human L1CAM antibody, 371602 (https://www.biolegend.com/fr-fr/products/purified-anti-human-cd171-l1cam-antibody-13167).
Anti-human C1QC antibody, ab247391 (https://www.abcam.com/en-us/products/primary-antibodies/c1qc-antibody-epr2984y-bsa-and-azide-free-ab247391?srsltid=AfmBOopLRh8fH89VLkFFwHjeH0m6x_cKWIOQAxFsYLxAm5K9-ayl01Qm).
Anti-human CD105 antibody, ab252345 (https://www.abcam.com/en-us/products/primary-antibodies/cd105-antibody-epr19911-220-ab252345?srsltid=AfmBOop-v8-29jTnkqMsGGDqgDytKhmdjtby4j0XBwmKAV_RAHYD7cf1).
Anti- human Calsyntenin-2 (CLSTN2) antibody, AF5480 (https://www.novusbio.com/products/calsyntenin-2-antibody_af5480?srsltid=AfmBOoqPhLsvygT4MHF91F9QJhXo5mqOn0Oluj0_2Nmt9W4gz5jLauPc).

Anti-human PPP2R2C Monoclonal antibody, H00005522-M01 (https://www.thermofisher.com/antibody/product/PPP2R2C-Antibody-clone-6D1-Monoclonal/H00005522-M01).
Anti-human PHOX2B antibody, ab216456 (https://www.abcam.com/en-us/products/primary-antibodies/phox2b-antibody-epr14423-bsa-and-azide-free-ab216456?srsltid=AfmBOooCV2yvCfVuAaP6z4bG3C7AxqL3bS2t9i7U3xuUktmY5DuiCJr2).
Anti-human BMP-7 antibody, MAB3541 (https://www.rndsystems.com/products/human-bmp-7-antibody-164311_mab3541).
Anti-human N-Myc antibody, 69006SF (https://www.cellsignal.com/products/primary-antibodies/n-myc-d4b2y-rabbit-mab-bsa-and-azide-free/69006?srsltid=AfmBOoqi9Bgh0DIaZ17eAe2JqFe2s-Ghjp77l5H6HJWdu_GjwqNhvCQ4).
Anti-human CD163 antibody, NB110-40686 (https://www.novusbio.com/products/cd163-antibody-edhu-1_nb110-40686?srsltid=AfmBOopNKfF2tzf6rO6VtWluC68ggNsGwn9V4ZtKGFReiRHyTbAKmt6g). Anti-human CD163 antibody has also been tested for CODEX in the below paper,
https://www.sciencedirect.com/science/article/pii/S0092867420308709#app2.
Anti-human Vimentin antibody, NBP1-97672 (https://www.novusbio.com/products/vimentin-antibody-rv202_nbp1-97672?srsltid=AfmBOoq2goGXvk04dqThrZ99VAHLqdtX0ySJ2tZvPv8DfBcZqdwA1JYO). The same clone RV202 has been validated on CODEX in the below paper, Reference: https://www.sciencedirect.com/science/article/pii/S0092867420308709#app2.
Anti-human Nestin antibody, MAB1259 (https://www.rndsystems.com/products/human-nestin-antibody-196908_mab1259).
Anti-human IL1RAPL1 antibody, NBP2-11648PE has been tested on Phenocyler fusion in house and has been shown to co-label with PHOX2B.
Anti-human CD2 antibody, ab131276 (https://www.abcam.com/en-us/products/primary-antibodies/cd2-antibody-epr6451-ab131276?srsltid=AfmBOop9p874apaVyj-Veg0slT7rAmX0B0Gsw20Ae-zdzXEko3Kg7r2T#).
Anti-human SV2C antibody, H00022987-M01 (https://www.novusbio.com/products/sv2c-antibody-3d8_h00022987-m01?srsltid=AfmBOork7csmRBm43OBGM7X0UnDULC3GmPJPDcVA-TiM-xZ3xm_lpwsa).

Akt, 9272, https://www.cellsignal.com/products/primary-antibodies/akt-antibody/9272
Phospho-Akt (Ser473) (p-AKT), 4060, https://www.cellsignal.com/products/primary-antibodies/phospho-akt-ser473-d9e-xp-rabbit-mab/4060
p44/p42 MAPK (Erk1/2), 9102, https://www.cellsignal.com/products/primary-antibodies/p44-42-mapk-erk1-2-antibody/9102
Phospho-p44/42 MAPK (Erk1/2) (Thr202/Tyr204), 4377, https://www.cellsignal.com/products/primary-antibodies/phospho-p44-42-mapk-erk1-2-thr202-tyr204-197g2-rabbit-mab/4377
α-Tubulin, 2125, https://www.cellsignal.com/products/primary-antibodies/a-tubulin-11h10-rabbit-mab/2125
HBEGF, MA5-35148, https://www.thermofisher.com/antibody/product/HBEGF-Antibody-clone-ARC0663-Recombinant-Monoclonal/MA5-35148
Beta-Actin, MA1140, https://www.thermofisher.com/antibody/product/beta-Actin-Antibody-clone-15G5A11-E2-Monoclonal/MA1-140
Anti-rabbit IgG-HRP linked, 7074, https://www.cellsignal.com/products/secondary-antibodies/anti-rabbit-igg-hrp-linked-antibody/7074
Anti-mouse IgG-HRP-linked, A5906, https://www.sigmaaldrich.com/US/en/product/sigma/a5906?msockid=39d4b736592f618a38dda470589f6008

Eukaryotic cell lines

Policy information about cell lines and Sex and Gender in Research

Cell line source(s)	Neuroblastoma (NBL) cell lines, NB1643, CHLA15, CHLA20, COG-N-297 and COG-N-590 were requested from the COG/ALSF Childhood Cancer Repository (www.CCcells.org). THP-1 (Cat # TIB-202) cell line was purchased from American Type Culture Collection (ATCC).
Authentication	All above cell lines were authenticated using STR profiling by the Penn Genomics and Sequencing Core at University of Pennsylvania.
Mycoplasma contamination	Mycoplasma test was done and no contamination was detected
Commonly misidentified lines (See ICLAC register)	No commonly misidentified cell lines were used.

Animals and other research organisms

Policy information about studies involving animals; ARRIVE guidelines recommended for reporting animal research, and Sex and Gender in Research

Laboratory animals

129x1/SvJ mice transgenic for the TH-MYCN construct were originally obtained from Bill Weiss (University of California, San Francisco). The six mice we used were aged 44,51,52, 52, 52,and 55 days, respectively.

TH-MYCN hemizygous mice were bred, and offspring were genotyped from tail-snip-isolated DNA using qPCR. Tumors are fully penetrant and arise at autochthonous sites in an immunocompetent host with lethality by day 60 of life.

Mice were monitored for tumors by palpation by a single experienced animal technician and randomized to a treatment arm when tumors were small to medium in size (~0.8 grams tumor by necropsy in n=3 control mice): vehicle (PBS) or 20 mg/kg dose of cyclophosphamide by intraperitoneal injection (IP) three times (Monday/Wednesday/Friday) for 2 weeks. Mice were weighed and assessed for tumor growth and symptoms at least three times weekly. Mice were euthanized for pre-defined humane endpoints related to overall health or tumor burden (hunching, immobility, hindlimb paresis, weight loss, respiratory distress).

For all experiments, mice were maintained at three to four mice per cage under humidity and temperature-controlled conditions with a light/dark cycle that is set at 12-hours. Animals were maintained under microisolator tops in a HEPA-filtered rack. Animals were fed autoclaved Purina mouse chow and water ad libitum. Handling was performed with universal sterile precautions and

	experienced personnel will perform all procedures. Mice were sacrificed, and tissues harvested, consistent with the recommendation of the Panel of Euthanasia of the American Veterinary Medical Association. Animal sacrifice is performed by administration of isoflurane or CO2 sedation followed by cervical dislocation. This methodology is consistent with the recommendations of the Panel on Euthanasia of the American Veterinary Medical Association.
Wild animals	No wild animals were used in this study.
Reporting on sex	The use of male or female mice was randomly selected for each patient sample.
Field-collected samples	No filed-collected samples were used in this study.
Ethics oversight	Mice were purchased from the Jackson Laboratory to establish a breeding colony at the Children's Hospital of Philadelphia (CHOP) and mouse bred in the colony were used for experiments. All animals were housed in the laboratory animal facility (LAF) vivarium in the Colket Translational Research Building (CTRB) at CHOP. The LAF is accredited by the American Association for Accreditation of Laboratory Animal Care (AAALAC), registered with the USDA and complies with the Public Health Service Policy on Humane care and Use of Laboratory Animals (Section: A3442-01). Additionally, the LAFs activities involving animals comply with the Guide for the Care and use of Laboratory Animals. Animal work in our laboratory was reviewed and approved by our Institutional Animal Care and Utilization Committee (21-000232, most recent re-approval 5/07/2024).

Note that full information on the approval of the study protocol must also be provided in the manuscript.

Clinical data

Policy information about clinical studies

All manuscripts should comply with the ICMJE guidelines for publication of clinical research and a completed CONSORT checklist must be included with all submissions.

Clinical trial registration	No study protocol was provided for the current study because this is profiled study for clinical and genomic analyses of surgical patients at the Children's Hospital of Philadelphia.
Study protocol	No study protocol was provided for the current study because this is profiling study for clinical and genomic analyses of surgical patients at the Children's Hospital of Philadelphia.
Data collection	Patients received standard therapy and excess tissue samples were utilized for genomic profiling. No additional interventions were performed and this study did not alter the clinical care of the patients in any way.
Outcomes	No pre-determined primary and secondary outcomes because this is profiling study for clinical and genomic analyses.

Plants

Seed stocks	N/A
Novel plant genotypes	N/A
Authentication	N/A
