# Supplementary material for: Longitudinal single-cell multiomic atlas of high-risk neuroblastoma reveals chemotherapy-induced tumor microenvironment rewiring
Source: Nat Genet. 2025 Apr 14;57(5):1142–54. doi: 10.1038/s41588-025-02158-6 (PMC12081299; doi:10.1038/s41588-025-02158-6)
Supplement: Supplementary file 10 — Unprocessed gels or blots. [file 41588_2025_2158_MOESM10_ESM.pdf]

Figure 6a: Pro-HB-EGF expression in THP1 macrophages

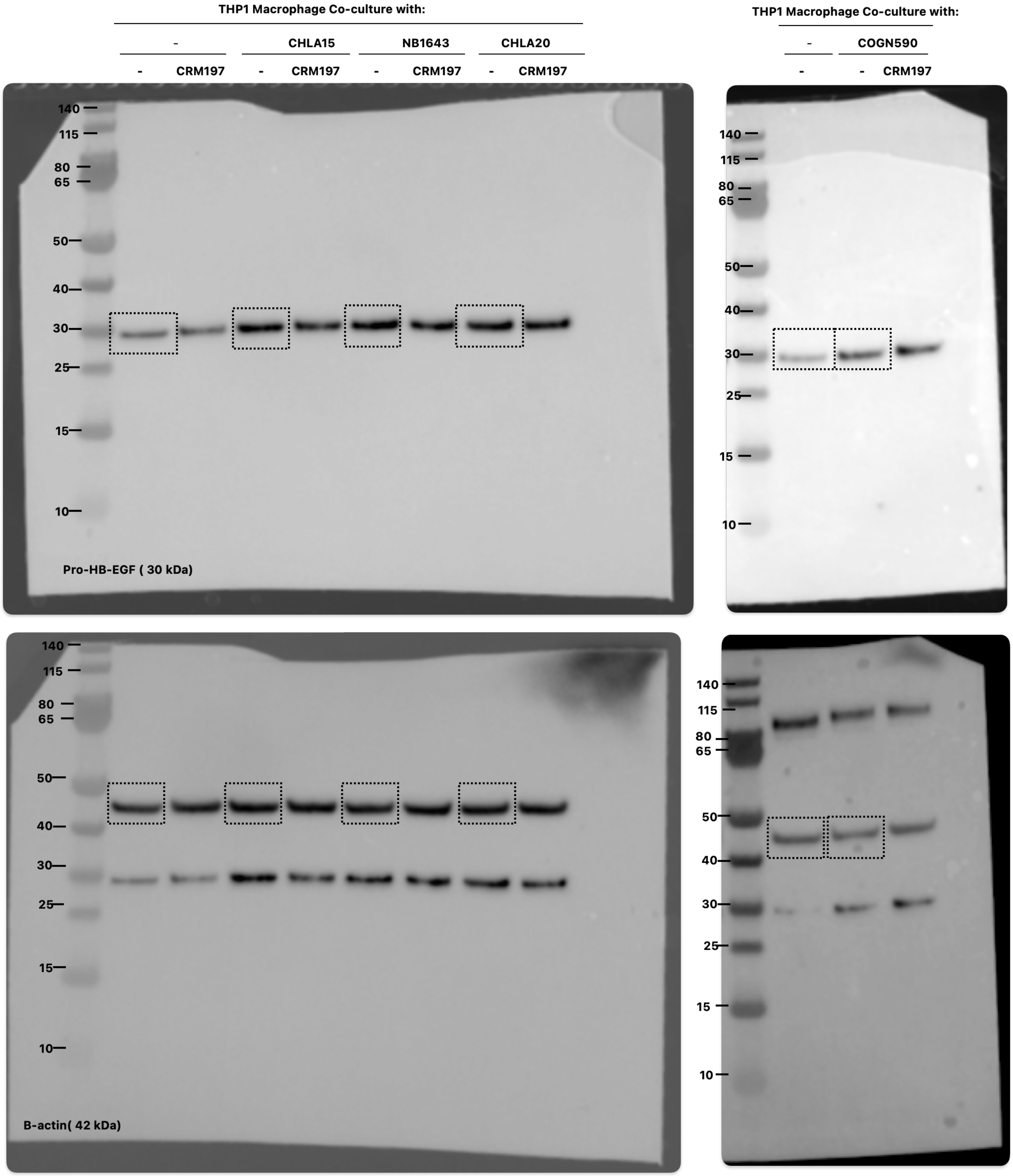

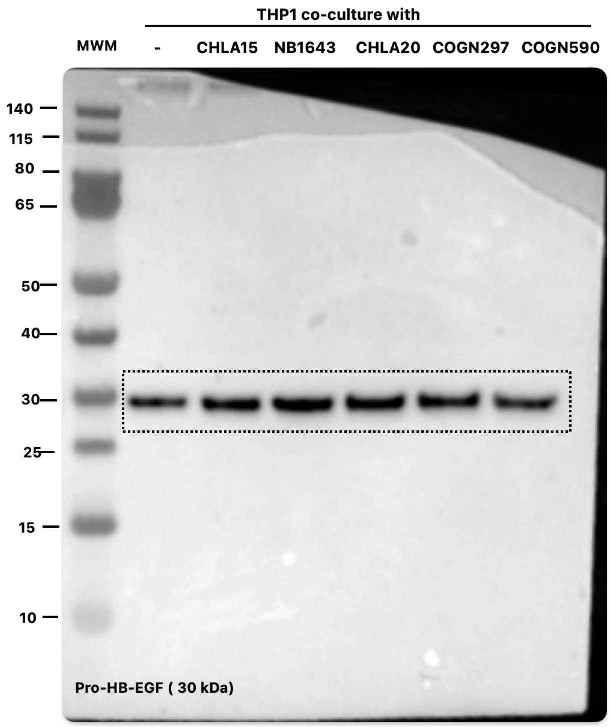
THP1 co-culture with
MWM
-
CHLA15
NB1643
CHLA20
COGN297
COGN590
140
115
80
65
50
40
30
25
15
10
Pro-HB-EGF (30 kDa)

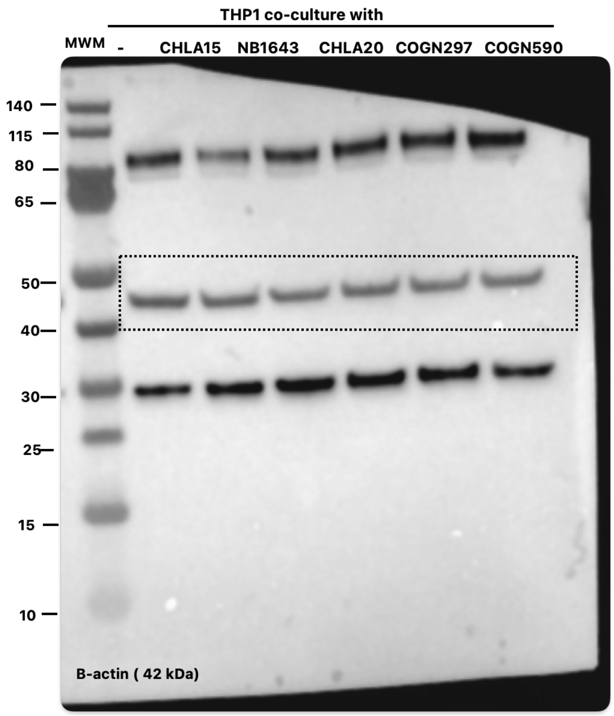
THP1 co-culture with
MWM
-
CHLA15
NB1643
CHLA20
COGN297
COGN590
140
115
80
65
50
40
30
25
15
10
B-actin (42 kDa)

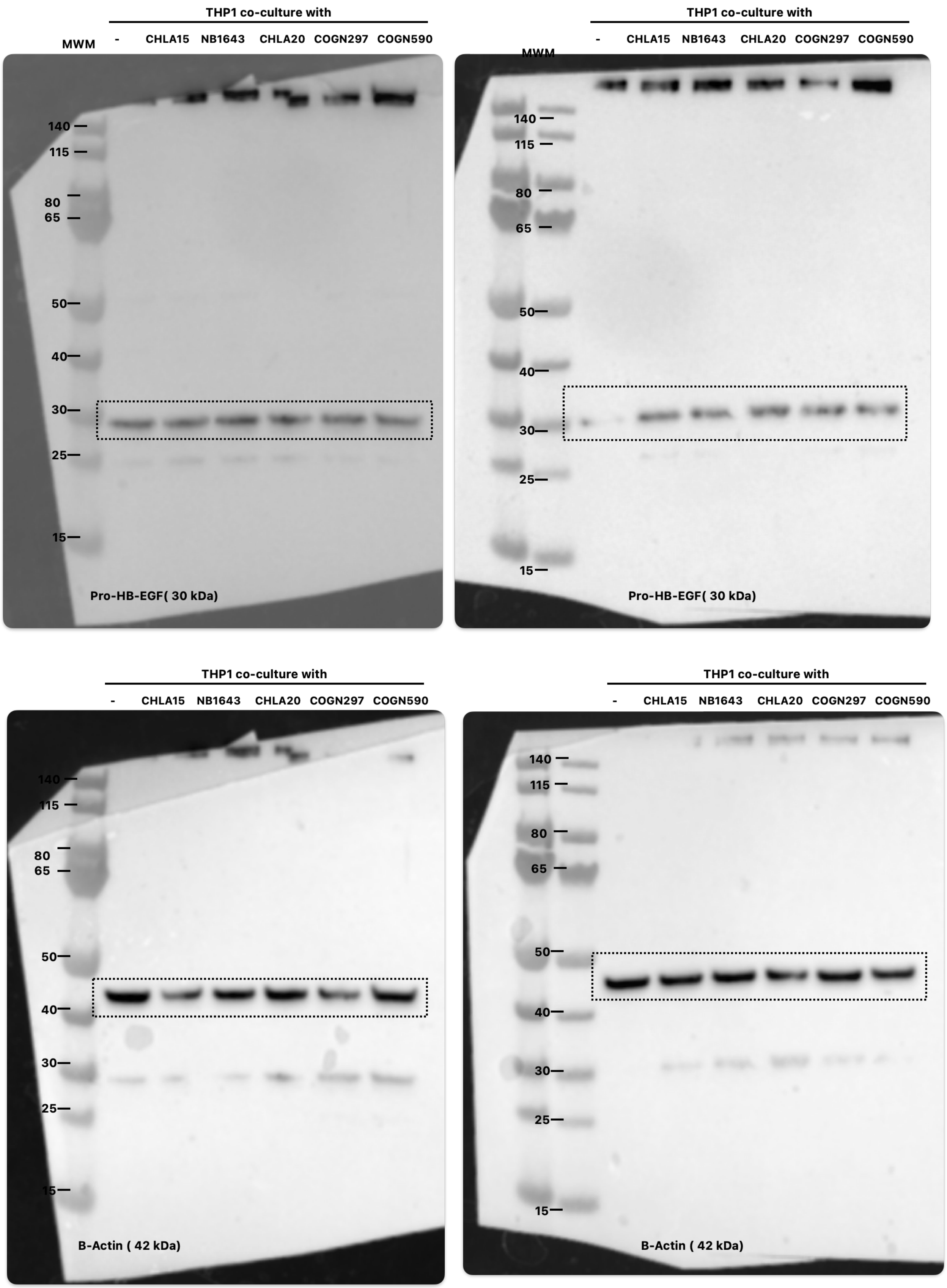
THP1 co-culture with
MWM
-
CHLA15
NB1643
CHLA20
COGN297
COGN590
140
115
80
65
50
40
30
25
15
Pro-HB-EGF(30 kDa)
THP1 co-culture with
MWM
-
CHLA15
NB1643
CHLA20
COGN297
COGN590
140
115
80
65
50
40
30
25
15
Pro-HB-EGF(30 kDa)
THP1 co-culture with
-
CHLA15
NB1643
CHLA20
COGN297
COGN590
140
115
80
65
50
40
30
25
15
B-Actin (42 kDa)
THP1 co-culture with
-
CHLA15
NB1643
CHLA20
COGN297
COGN590
140
115
80
65
50
40
30
25
15
B-Actin (42 kDa)

Figure 6e: ERK phosphorylation in THP1 macrophage co-cultured NBL cells:

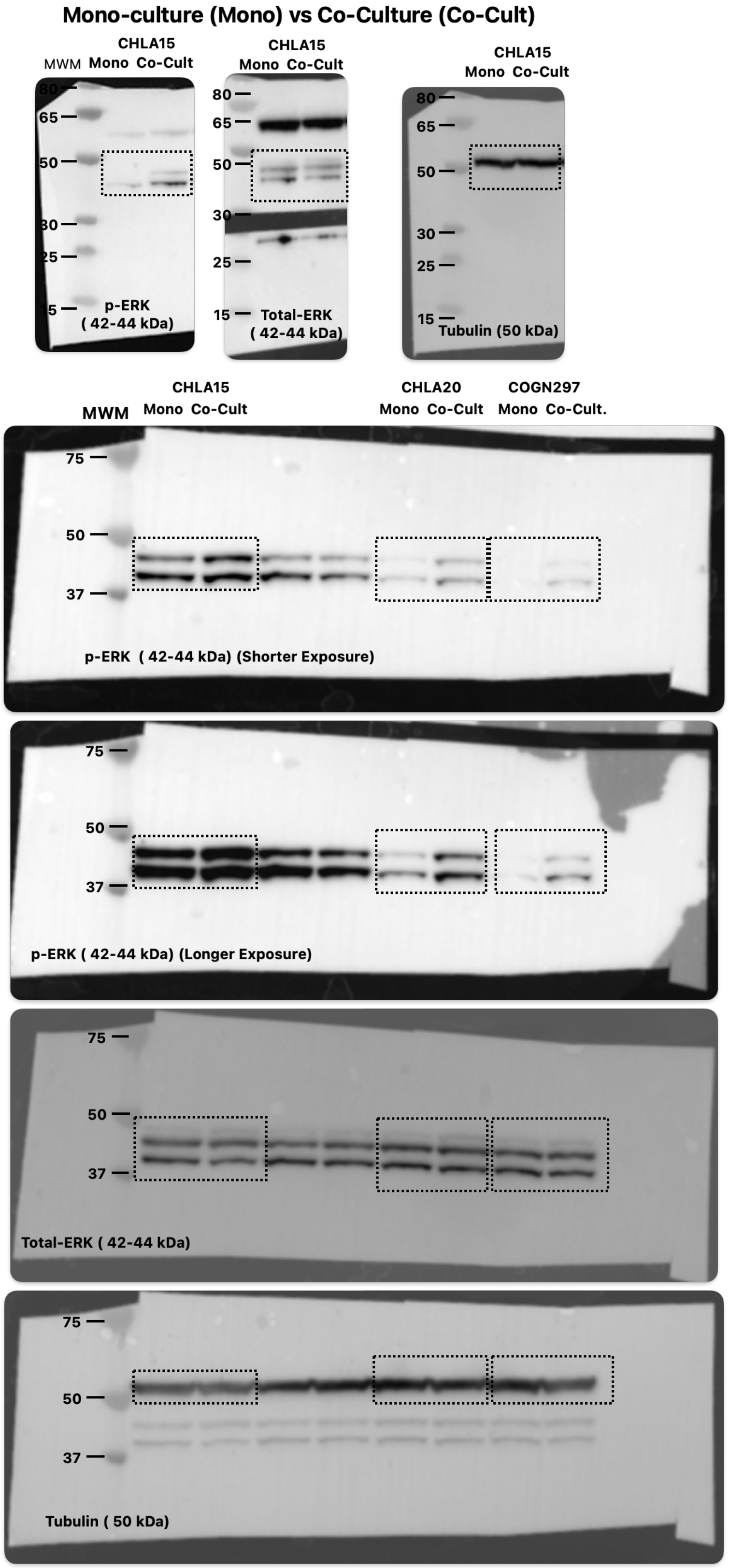

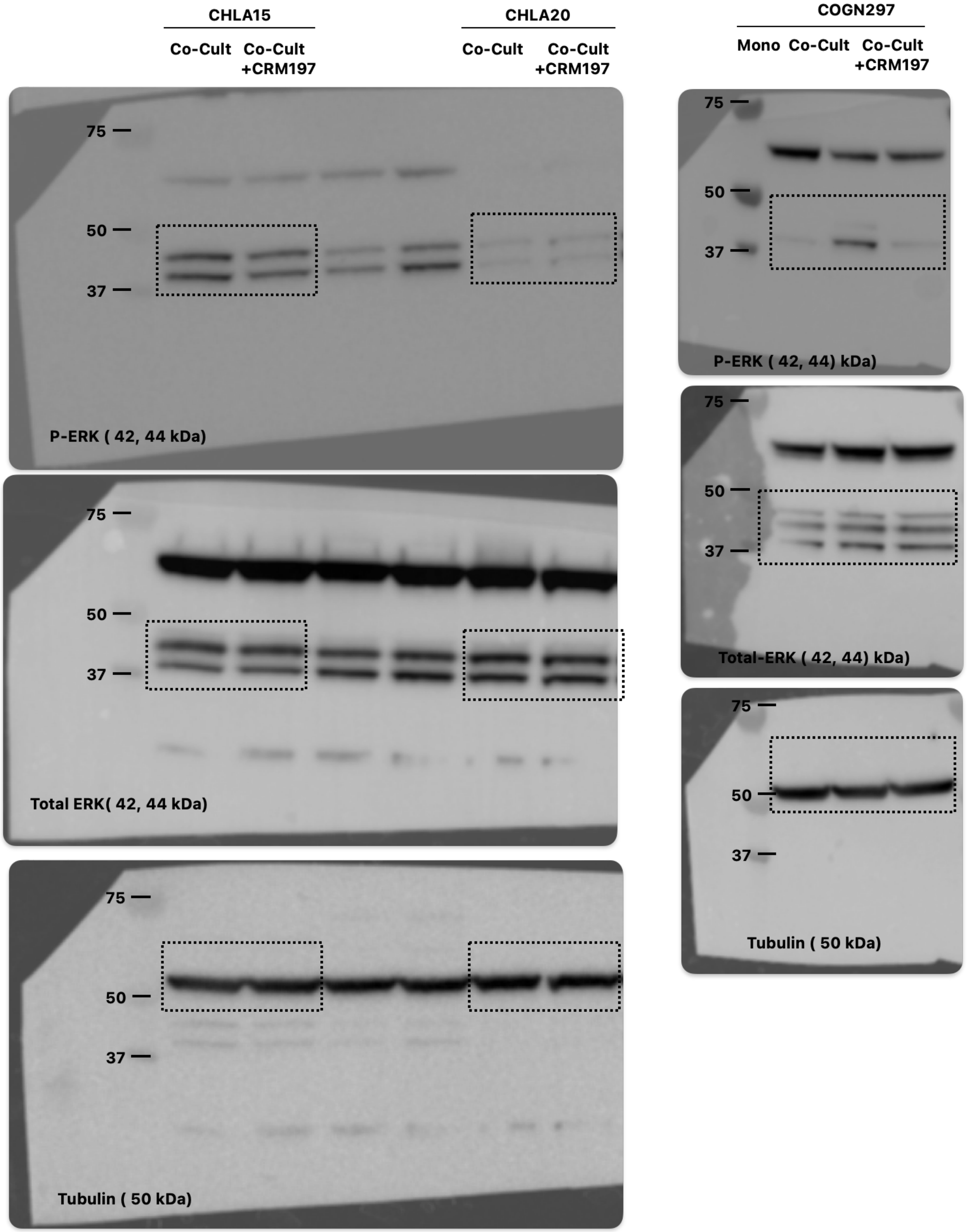
CHLA15
Co-Cult
Co-Cult +CRM197
CHLA20
Co-Cult
Co-Cult +CRM197
COGN297
Mono
Co-Cult
Co-Cult +CRM197
75
50
37
P-ERK (42, 44 kDa)
Total ERK(42, 44 kDa)
Tubulin (50 kDa)
P-ERK (42, 44) kDa)
Total-ERK (42, 44) kDa)
Tubulin (50 kDa)

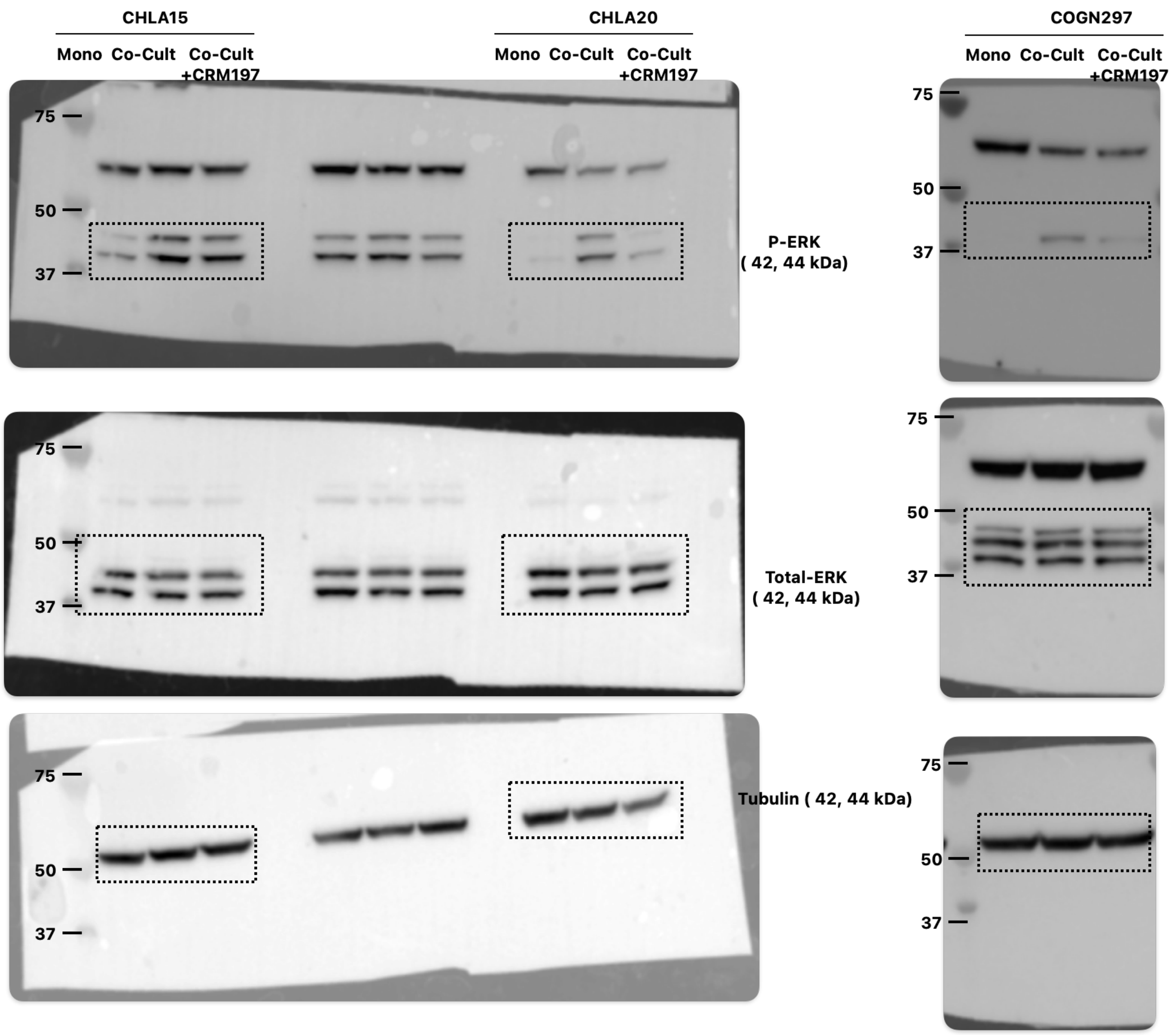
CHLA15
Mono
Co-Cult
Co-Cult +CRM197
CHLA20
Mono
Co-Cult
Co-Cult +CRM197
COGN297
Mono
Co-Cult
Co-Cult +CRM197
75
50
37
P-ERK (42, 44 kDa)
Total-ERK (42, 44 kDa)
Tubulin (42, 44 kDa)

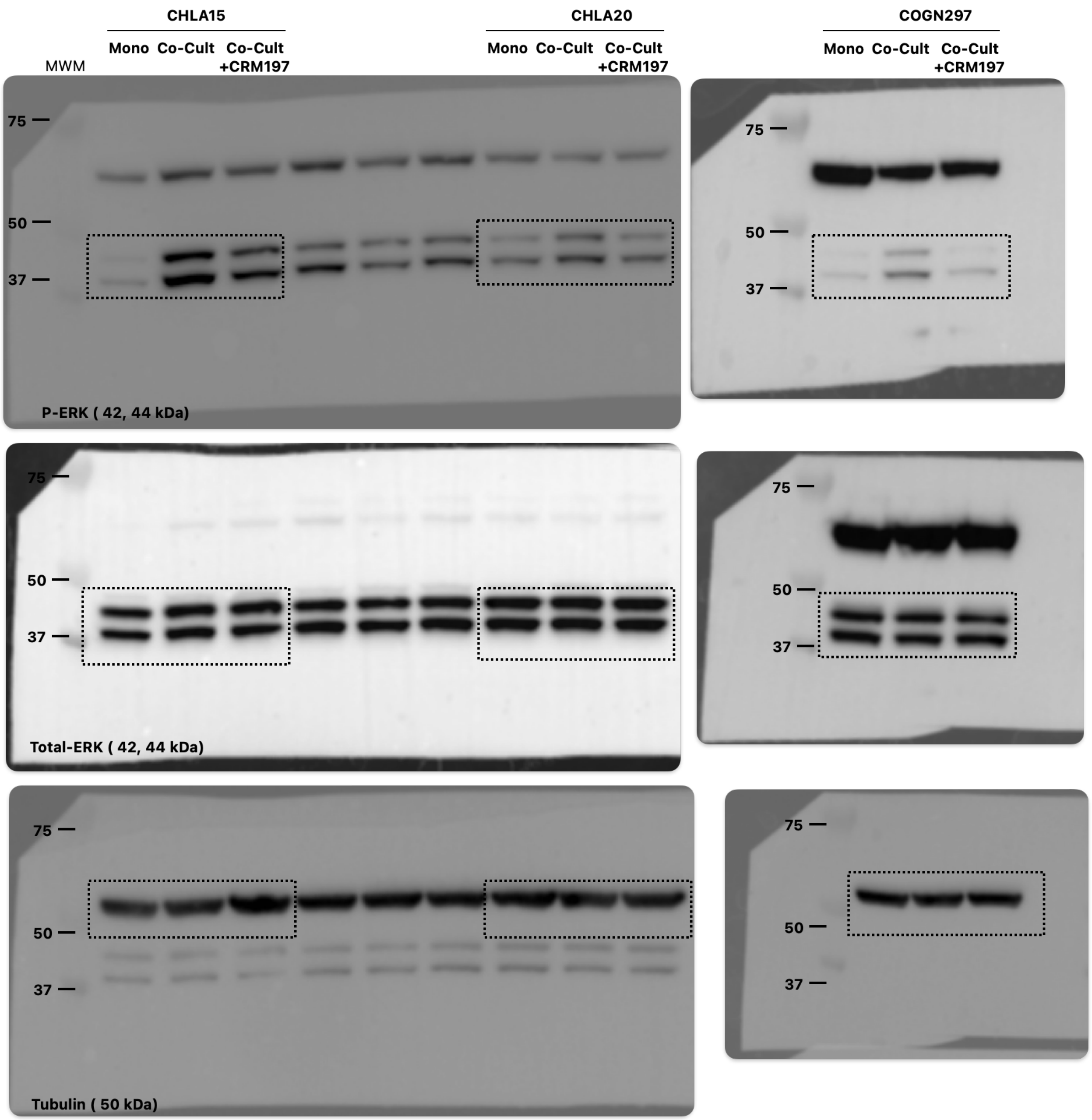
CHLA15
CHLA20
COGN297
Mono
Co-Cult
Co-Cult +CRM197
MWM
75
50
37
P-ERK (42, 44 kDa)
Total-ERK (42, 44 kDa)
Tubulin (50 kDa)

Figure 6g: AKT phosphorylation in THP1 macrophage co-cultured NBL cells:

Mono-culture (Mono) vs Co-Culture (Co-Cult)

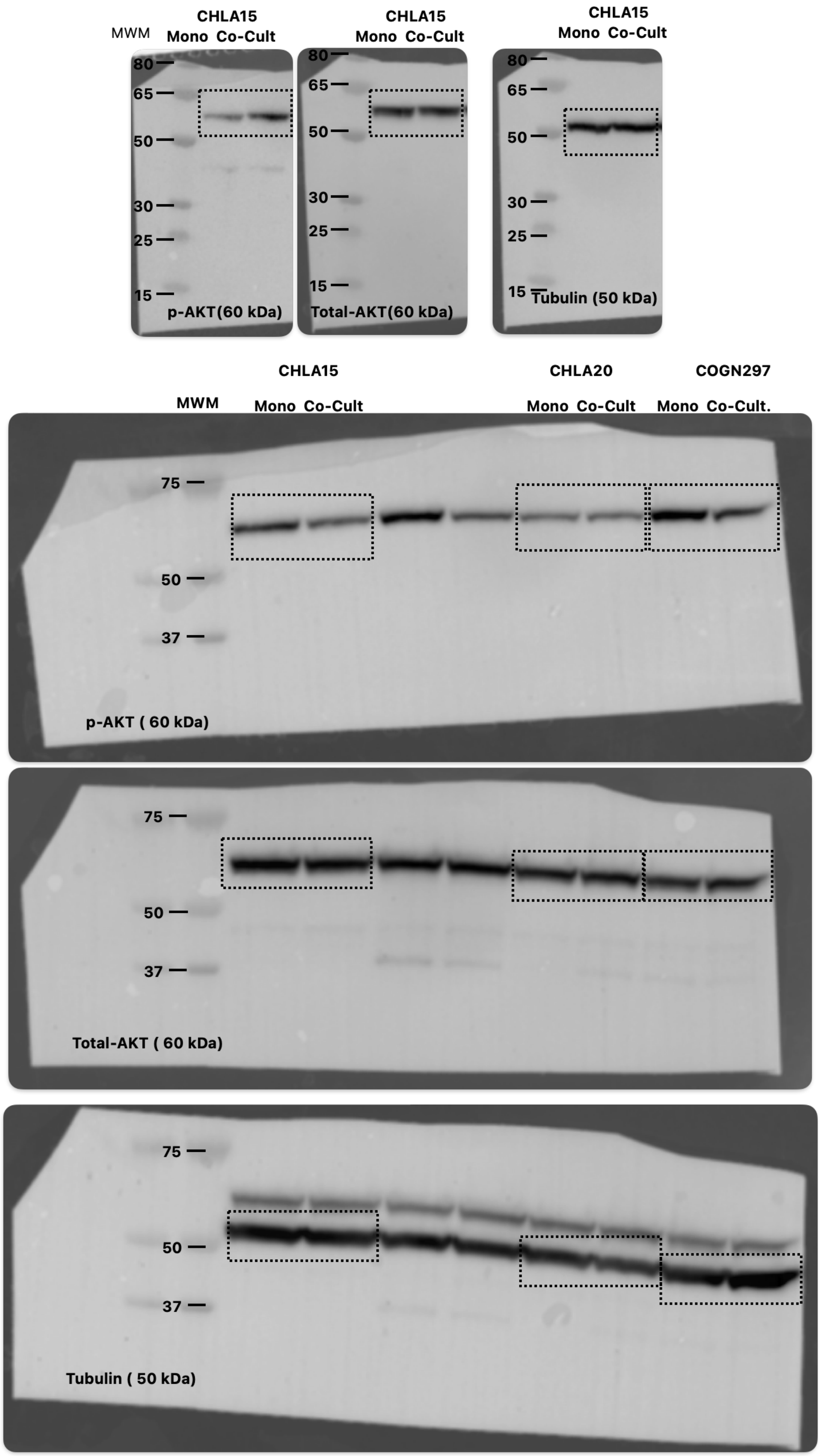

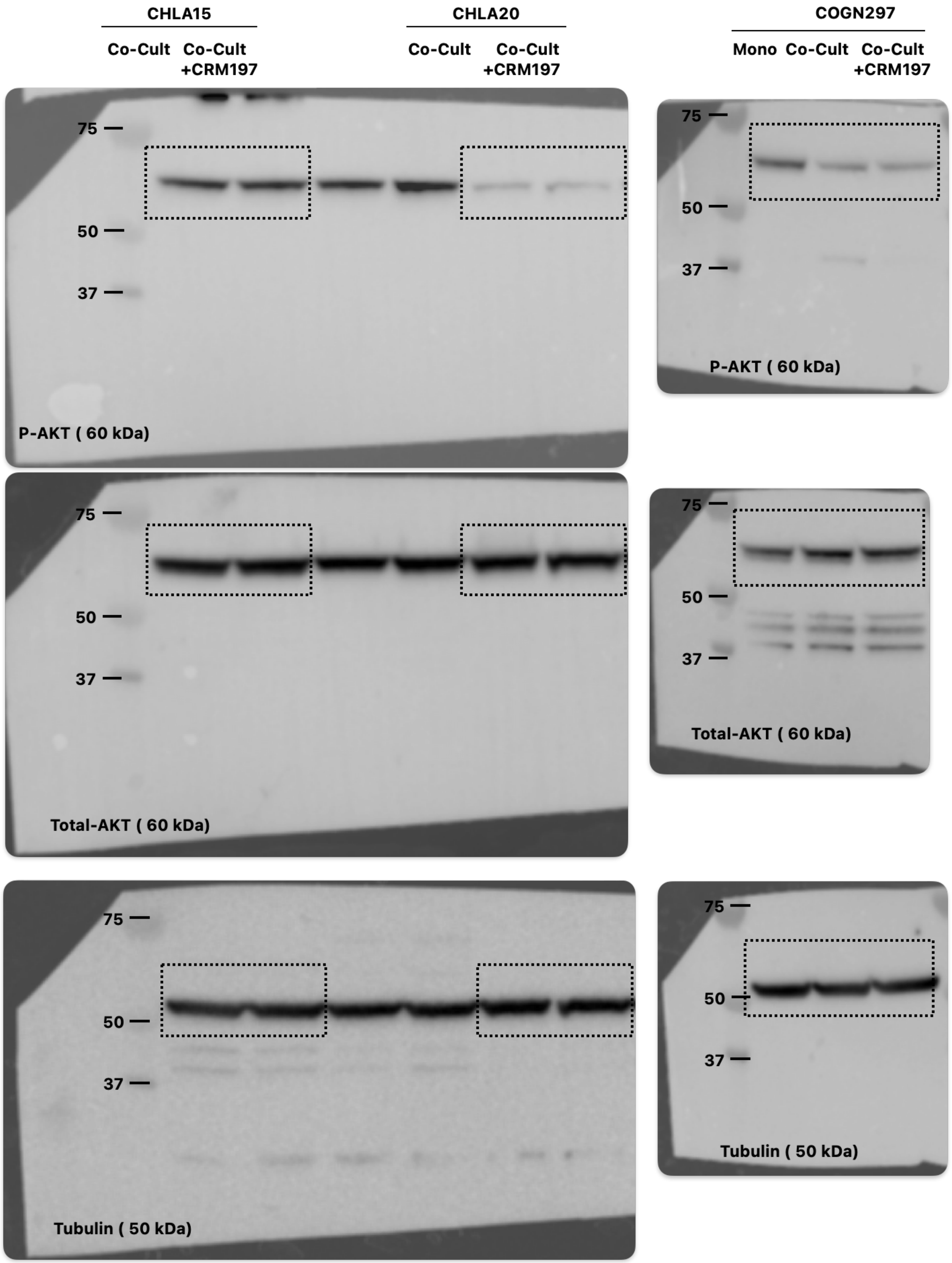
CHLA15
Co-Cult
Co-Cult +CRM197
CHLA20
Co-Cult
Co-Cult +CRM197
COGN297
Mono
Co-Cult
Co-Cult +CRM197
75
50
37
P-AKT (60 kDa)
Total-AKT (60 kDa)
Tubulin (50 kDa)

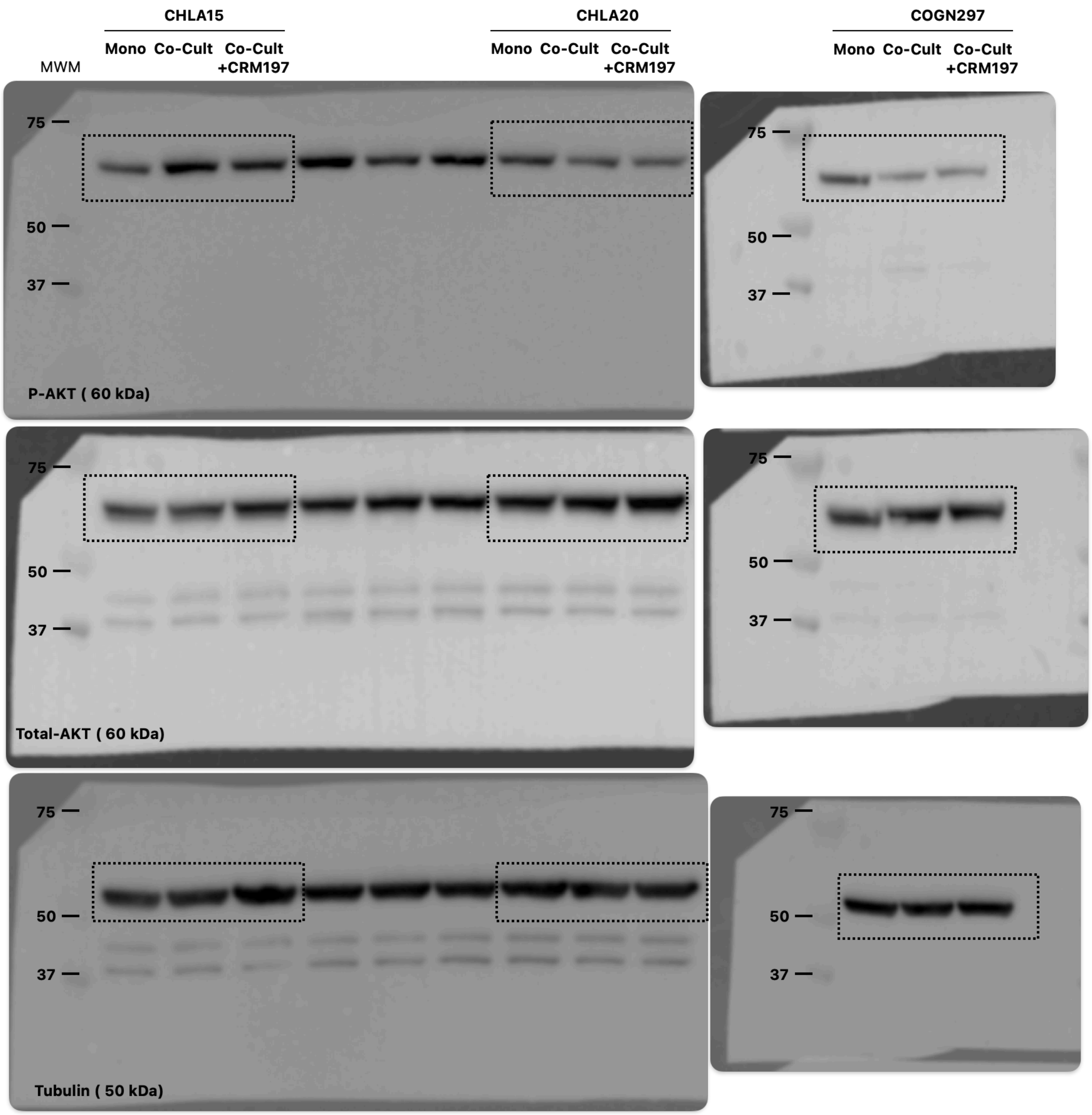
CHLA15
CHLA20
COGN297
Mono
Co-Cult
Co-Cult +CRM197
MWM
75
50
37
P-AKT (60 kDa)
Total-AKT (60 kDa)
Tubulin (50 kDa)

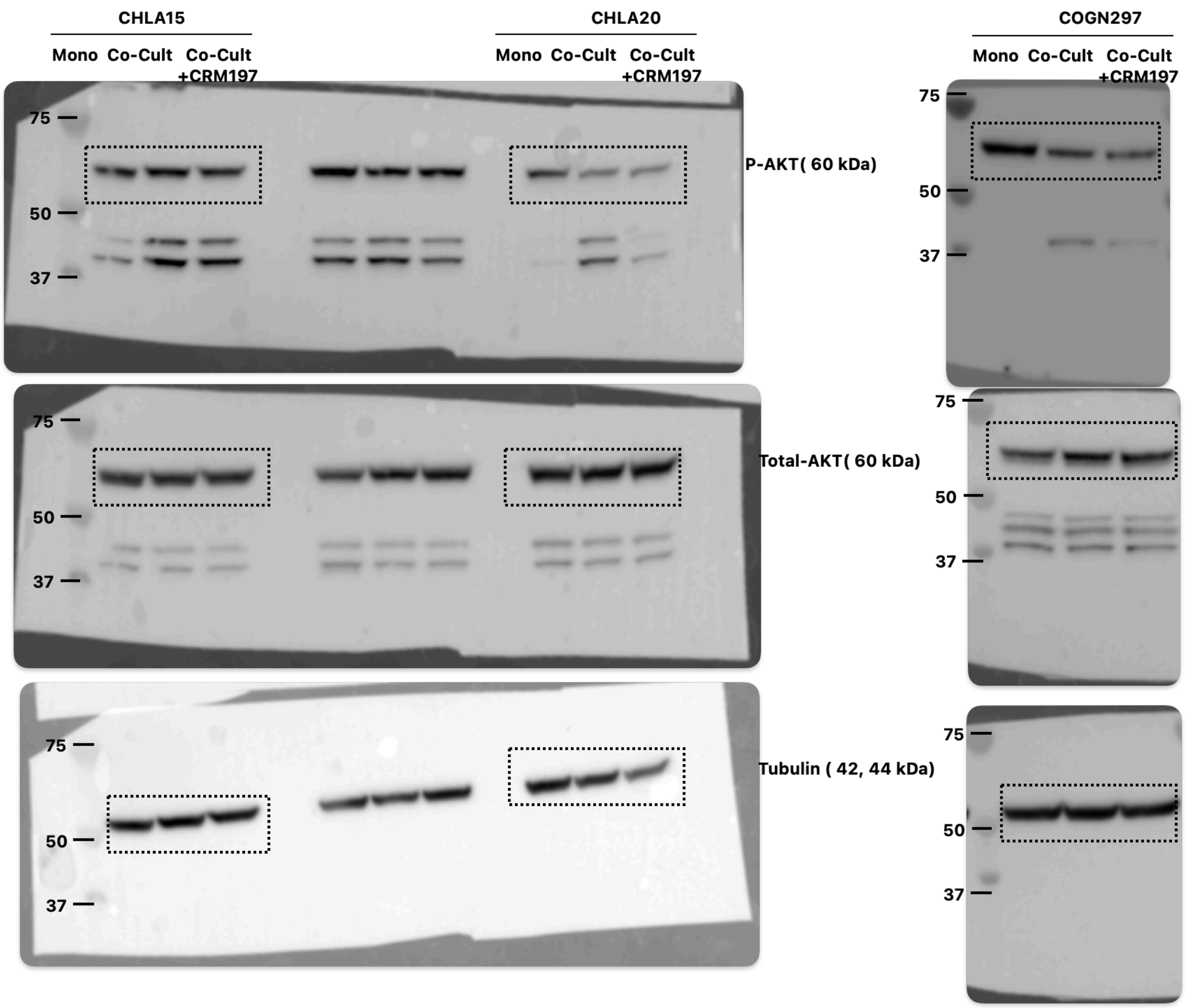
CHLA15
CHLA20
COGN297
Mono
Co-Cult
Co-Cult +CRM197
P-AKT(60 kDa)
Total-AKT(60 kDa)
Tubulin (42, 44 kDa)
75
50
37
